# Analysis on conservation of disulphide bonds and their structural features in homologous protein domain families

**DOI:** 10.1186/1472-6807-8-55

**Published:** 2008-12-26

**Authors:** Ratna R Thangudu, Malini Manoharan, N Srinivasan, Frédéric Cadet, R Sowdhamini, Bernard Offmann

**Affiliations:** 1 Laboratoire de Biochimie et Génétique Moléculaire, Université de La Réunion, BP 7151, 15 avenue René Cassin, 97715 Saint Denis Messag Cedex 09, La Réunion, France; 2 National Centre for Biological Sciences, GKVK Campus, Bangalore, India; 3 Molecular Biophysics Unit, Indian Institute of Science, Bangalore 560 012, India; 4 National Center for Biotechnology Information, U.S. National Library of Medicine, 8600 Rockville Pike, Bethesda, MD 20894, USA

## Abstract

**Background:**

Disulphide bridges are well known to play key roles in stability, folding and functions of proteins. Introduction or deletion of disulphides by site-directed mutagenesis have produced varying effects on stability and folding depending upon the protein and location of disulphide in the 3-D structure. Given the lack of complete understanding it is worthwhile to learn from an analysis of extent of conservation of disulphides in homologous proteins. We have also addressed the question of what structural interactions replaces a disulphide in a homologue in another homologue.

**Results:**

Using a dataset involving 34,752 pairwise comparisons of homologous protein domains corresponding to 300 protein domain families of known 3-D structures, we provide a comprehensive analysis of extent of conservation of disulphide bridges and their structural features. We report that only 54% of all the disulphide bonds compared between the homologous pairs are conserved, even if, a small fraction of the non-conserved disulphides do include cytoplasmic proteins. Also, only about one fourth of the distinct disulphides are conserved in all the members in protein families. We note that while conservation of disulphide is common in many families, disulphide bond mutations are quite prevalent. Interestingly, we note that there is no clear relationship between sequence identity between two homologous proteins and disulphide bond conservation. Our analysis on structural features at the sites where cysteines forming disulphide in one homologue are replaced by non-Cys residues show that the elimination of a disulphide in a homologue need not always result in stabilizing interactions between equivalent residues.

**Conclusion:**

We observe that in the homologous proteins, disulphide bonds are conserved only to a modest extent. Very interestingly, we note that extent of conservation of disulphide in homologous proteins is unrelated to the overall sequence identity between homologues. The non-conserved disulphides are often associated with variable structural features that were recruited to be associated with differentiation or specialisation of protein function.

## Background

Cysteine residues assume important role in proteins through a wide range of functions such as disulphide bond formation, metal binding, electron donation, hydrolysis and redox catalysis. Disulphide bond formation is one of the most important post-translational modification events of a protein in the biological cell. Disulphide bond stabilization of a protein is considered to be entropy driven through destabilization of the unfolded state and may also contribute enthalpically through favourable local interactions like compacting the clusters of hydrophobic residues. Knowledge of disulphide bond connectivity is influential in protein folding experiments and in 3-D structure prediction.

Since Richardson's and Thornton's extensive and detailed analysis on disulphide bonds in 1981, several studies have been reported on the oxidation state of cysteines and the conservation, connectivity and structure of disulphide bonds [1,2]. Several computational methods have been developed to predict or model cysteine sidechains that might be involved in disulphide formation [3-17] and also to identify their connectivity patterns in multiple disulphide bond containing proteins [18-24]. Tools are available to model disulphide bonds in proteins by estimating the local stereochemical compatibility to accommodate a disulphide bond [25,26].

Disulphide bonds are generally believed to be conserved among related proteins [1,27] and the cystine connectivity pattern may be used as a diagnostic to identify proteins of similar 3-D structure. An inverse approach starting with clearly related proteins aims to identify cystine connectivity pattern using sequence alignments[28]. Mas and co-workers have derived relationship amongst even non-homologous proteins belonging to different superfamilies [29]. They explained the antagonistic properties of potato carboxypeptidase inhibitor against growth factors by comparing its structural features with epidermal growth factor, derived through their disulphide bridge topology even when their connectivity differs [29]. The conservation of disulphide bond connectivity patterns, enable the identification of remote homologues even when the most popular sequence search methods may fail to do so. Such approaches are complicated by observations of topologically equivalent disulphide bonds in non-homologues and also by non-equivalent number of disulphide bonds in close homologues [29].

Many studies examined the role of disulphide bonds in the protein structure and function, some through mutagenesis experiments [30-38], while a few others studied the same in evolutionary perspective [39]. Thornton observed that in protein superfamilies the conservation rules appear less stringent [2] from the analysis of limited data. Non-conservation in such cases is usually associated with loss of both the cysteines involved in the disulphide bond [1,2]. Amino acids linked by a specific role mutate in a coordinated manner if the geometry of the contacts are the same in all the proteins [40]. Kreisberg and co-workers employed multiple sequence alignments to map patterns of correlated cysteine mutations to identify positions of protein disulphide bonds [41].

The high levels of structural and functional similarity among several distantly homologous proteins overwhelm protein classification methods that rely on simple sequence identity. The SCOP [42] database contains several families with mean pairwise sequence identity among the members of the family well below 40%. Hence, there could be significant changes in structure or function due to the presence or absence of one or more disulphide bonds in a protein compared to its homologue. This necessitates a comprehensive study of disulphide bond variations in homologous protein domains.

It has been documented that ligand bound cysteines are more similar to disulphide bonded cysteine than free cysteines [7,8]. However, we have confined the present study to disulphide bonded cysteines as many of the disulphide bond containing proteins are extracellular or secreted whereas there are many intracellular proteins with cysteines liganded to metal ions (such as zinc coordinated cysteines in transcription factors which are nuclear proteins),

A systematic study of structural conservation of disulphide bonds in homologous protein domains should reduce the limitations of sequence comparison in principle. But this raises several interesting questions: to what extent sequence similarity guarantees the conservation of disulphide bonds in related proteins? What is the structural or functional consequence of non-conservation? Do the locations of non-conserved disulphide bonds exhibit any similarities? To address these questions, we attempted a large-scale analysis of conservation of disulphide bonds in the homologous families of SCOP [42]. In principle, it will be more definitive as well as interesting to uplift the entire analysis after including sequence homologues. However, it was imperative that we restrict the current analysis to structural entries within homologous families only so that we do not have to compensate for the quality of alignments nor make any assumptions on disulphide bond connectivity in the homologues of yet unknown structure. Understanding the consequences of such natural variations in native proteins should help in overcoming the unfavourable effects of mutational experiments, like disruption of native structure [43,44]. Implications of this analysis for prediction of disulphide connectivity and protein modelling are also discussed.

## Results

### Distribution of disulphide bonds in homologous protein families

Our dataset for analysis comprises a total of 300 homologous families from SCOP, each populated with at least two members and where at least one of them is annotated with a disulphide bond. A total of 3990 disulphide bonds are present in these 300 families. The distribution of disulphide bonds in different fold classes is depicted in Table 1. Disulphide bonds are common in all fold classes, although in varying degrees. However, only 22% of the families in SCOP contain annotated disulphide bonds. A closer look at these families might provide clues about their preferential distribution. Small proteins with less than 70 residues, by the SCOP definition, are usually rich in disulphide bonds which are known to contribute critically to their stability[45] since small proteins usually lack a strong hydrophobic core[46]. Disulphides are known to exist in most small proteins[47,48]. Indeed, a large majority (67%) of the small protein families in SCOP 1.67 have disulphide bonds (Table 1).

**Table 1:** Fold distribution of disulphide bond containing families under study in SCOP and number of disulphides and distinct disulphides the database features.

	**Number of disulphide bond containing families**		**Number of members per family in ANALYCYS**			**Number of disulphides**	
	
**SCOP^a ^Class**	**SCOP**	**ANALYCYS^b^**	**2**	**3–10**	**> 10**	**No of disulphides**	**No of distinct disulphides**
All α (550)^c^	65	38	12	17	9	524	125
All β (529)	167	89	19	46	24	961	256
α/β (593)	111	58	8	30	20	427	192
α+β (650)	106	59	16	30	13	640	141
Small proteins (162)	108	56	9	35	12	1438	262

Total (2630)	580	300	64	158	78	3990	976

^a ^Structural classification of proteins.

^b ^Database generated in the current analysis of disulphide conservation in SCOP families.

^C ^The numbers between parentheses represents the total number of families in the different SCOP class

On the other hand, all-α proteins showed least preference for these crosslinks, probably because of the steric hindrances involved in the formation of disulphides between cysteines present in different helices or same helix. The repertoire of cysteine conformations suggests preferences in disulphide distribution within beta sheets[49]. The distribution of disulphide bridges in terms of secondary structure highlights their preference for stabilizing strands and loops (see later).

### Analyzing disulphide conservation using relaxed criterion

In our study, a disulphide bond is considered strictly conserved in a multiple alignment if both the cysteines involved are placed at equivalent positions in all the sequences in the alignment. However, due to slight changes in the conformations, rigid body shifts and uncertainties in the structure-based alignments some times potentially equivalent cystines from homologues may not align perfectly. Hence, according to the relaxed criteria, an arbitrary shift of 4 residues on either side of the cystine positions is used for counting the distinct disulphide bonds.

A total of 34752 pairwise comparisons between members within each 300 families were analysed. The number of conserved disulphide bonds in these homologous pairs rose from 19500 to 26065 when the relaxation was applied. The increase, although marginal, indeed points out to the potential structural variation in homologous proteins. Only 54% of all the disulphide bonds compared between the homologous pairs are conserved.

#### A small fraction of the proteins with cytoplasmic localisation lack conserved disulphide bonds

Only 52 proteins in the current dataset, predicted to be cytoplasmic (see Additional files), were reported to be cytoplasmic after extensive literature searches. This amounts to a minor fraction (2%) of proteins in the entire dataset employed for the analysis. More importantly, only 38 proteins in the current dataset are reported to be cytoplasmic and do not share conservation of disulphide bonds (1.5%), suggesting that the dataset is not likely to be biased by protein families with diverse cellular localisation that can affect the low conservation of disulphide bonds. Most of such rare and peculiar examples are oxidoreductases like thioredoxins where disulphides are essential for their function.

**Figure 1:**
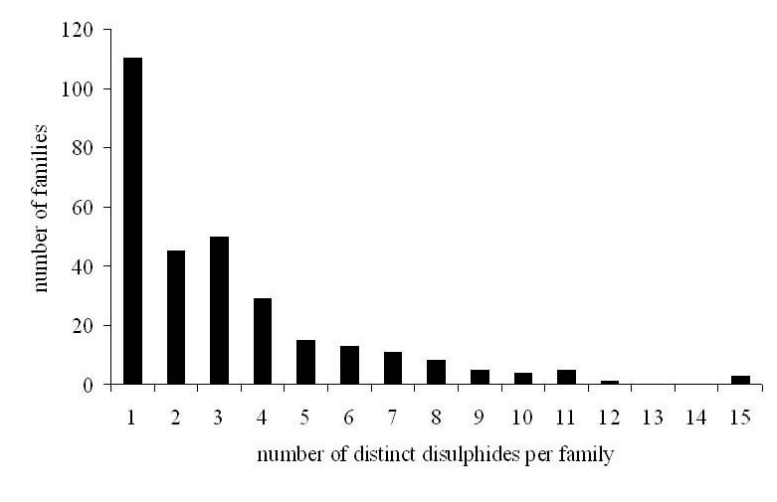
**Distribution of total number of distinct disulphides per family**.

In the family based multiple structural alignments, all the distinct disulphide bonds in a given family were identified and their conservation state were estimated (see Materials and Methods). Overall, a total of 976 distinct disulphides were featured across all 300 families in our dataset. The total number of distinct disulphides per family varies from 1 to 15 with an average of 3.33 disulphides (Figure. 1). About one third of the families (108) possess only a single distinct disulphide. The number of distinct disulphides in small proteins ranges from 1 to 15 but with an average of 4.53. A total of 14 families display more than 10 distinct disulphides. The highest number of distinct disulphides that was found in a family is 15 and was found in the family of sialidases/neuraminidases (SCOP code: b.68.1.1), the beta-glycanases family (SCOP family code: c.1.8.3), growth factor receptor domain (SCOP family code: g.3.9.1) and the transferrin family (SCOP family code: c.94.1.2). Since this classification is at individual domain level, we might not encounter highly disulphide-rich multi-domain systems, such as wheat-germ agglutinin or serum albumin-like folds.

**Figure 2:**
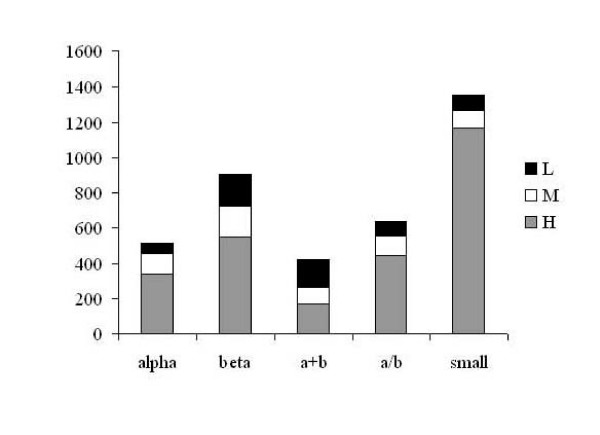
**Distribution of highly (H), medium (M) and low (L) conserved disulphides in SCOP database**.

We further classified the distinct disulphides for their conservation into three categories: high (H), medium (M) and low (L) conserved disulphide bonds. As shown in Figure. 2, their distribution in different fold classes, clearly establishes interesting facts. While the majority of the disulphide bonds in small proteins are highly conserved, re-establishing the fact that disulphide bonds play a strong role in their structural stabilization, they are less conserved in other fold classes. Hence, in general, conservation of disulphide bonds among homologues of globular proteins is only modest.

Further, analysis of the half-cystine containing regions was performed to investigate whether the associated local structures were topologically conserved (see Material and Methods) in homologous proteins along with the disulphide bond itself. Our analysis suggests that in about 70% of the disulphide bonds, at least one of the half-cystine is localized in structurally conserved region. This is rather interesting since disulphide bonds are reputed to occur preferentially in loop regions or connect regular secondary structures with coiled regions[7] (see later). The highly variable nature of loops/coils did not deter the conservation of local structures where the disulphides are found, strongly suggesting their role in structural stabilization. Similarly, in almost 80% of the disulphide bonds where the local structures of the participating half-cystines are in structurally variable regions, at least one half-cystine is in coil state.

#### Low-conserved disulphide bonds stabilise insertion regions specific to a subfamily

When the conservation status of the disulphides was analysed, we found that most of the highly conserved disulphide bonds have at least one half-cystine in topologically equivalent region. However, in poorly conserved disulphide bonds, more than 50% of their half-cystines lie in structurally variable regions. This suggests that a low-conserved disulphide bond in a family member is an adaptation and usually tends to constrain highly structurally variable regions of the family. For example, trypsin-like serine proteinases share a common serine proteinase fold, and often have a sequence identity below 30% with diverse functions. The poorly conserved disulphide bonds in this family appeared to stabilise insertion regions specific to a subfamily. The 12–218 (alignment positions) disulphide bond in Factor B (C1–C122) associates the insertion, N-terminal linker to the main body of the serine proteinase [50] and is highly conserved only in plasminogen and chymotrypsinogen[51] The helix-turn-helix insertion (T125a-D133) unique to factor B SP (1dle) is stabilised by constraining through the distinct 221–241 disulphide bond (C125-C125p)[50]. The 156–489 bridge is specific to snake venom serine proteinases, clamps the C-terminal tail to the 99-loop that surrounds the active site[52]. All these three poorly conserved distinct disulphide bonds of this family did not have any topologically equivalent alignment partners in non-conserved members and had at least one of the cysteine aligned with a gap. Another spectacular example is the melanoma inhibitory activity (MIA) protein. It is the first structure of a secreted protein with a Src homology 3 (SH3)-like domain with N- and C-terminal extensions of about 20 residues each adding additional structural elements, forming a previously undescribed fold. The 12–17 and 35–106 disulphide bonds, unique to this protein scaffold, stabilise loop regions and are not observed in classical SH3 domains. The structure also suggests a likely protein interaction site and suggests that, unlike conventional SH3 domains, MIA does not recognize polyproline helices and may have evolved with different functions[53].

### Disulphide conservation versus number of disulphide bonds per family

Totally conserved disulphides are expected to play an important role in the structure and/or function of homologous proteins. Also, it is of interest to analyze the structural and functional roles of single disulphides that are featured in some of the protein families. Here, we assess the possible relationship between extent of disulphide conservation and 'disulphide richness' in protein families.

**Figure 3:**
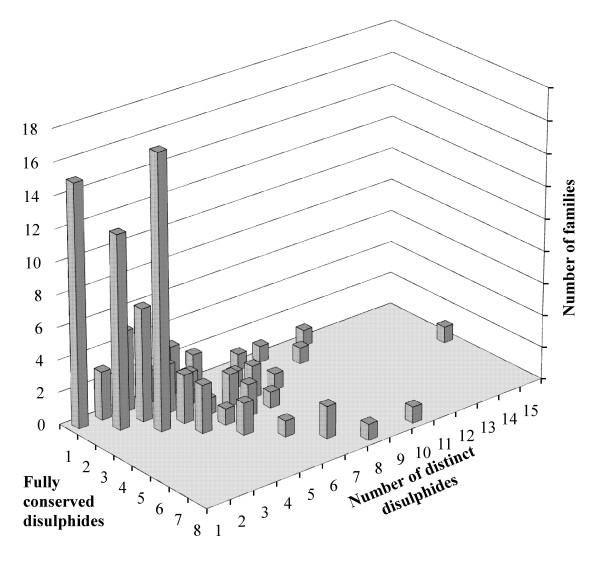
**Analysis of fully conserved disulphides in SCOP families**. Distribution of number of fully conserved disulphides against number of distinct disulphides per family.

As shown in the previous section, not all the disulphide bonds in a family are equally conserved. One fourth of the all the distinct disulphides (237 out of 948) are totally conserved (i.e. in all the family members). Interestingly, the topological state of a vast majority of these 237 fully conserved disulphides is also highly conserved. The topological equivalence of cysteine residues participating in disulphide bond in family of proteins inherently highlights the conservation of structural state (secondary structure) and also solvent accessibility. These fully conserved disulphides, as shown in Figure. 3, are distributed in as much as 33% (100 families) of the total number of families in our dataset thus indicating that full conservation of disulphides is not widespread but is not rare either. The maximum number of fully conserved disulphides that a family displays in our dataset was eight (Figure. 3) and was featured in osmotin thomatin-like protein family (b.25.1.1) and in glycosyl hydrolase family 7 catalytic core (b.29.1.10). In the former, all eight distinct disulphides are indeed fully conserved and the pairwise sequence similarities between all four individual members varied between 54.7% and 79.5%. In the latter, 8 out of 10 distinct disulphides that are featured in the family are fully conserved and the pairwise sequence similarity between all four members in the family ranged from 40.5% and 56.8%.

Further analysis of the content of fully conserved disulphides out of the total number of distinct disulphides per family, as illustrated in Figure. 3, shows that a total of 51 families are found to have all their disulphides conserved (diagonal on the plot). However, out of these 51 families, 50% have only 2 members in the data set and almost 90% of them have ≤ 4 members. It is noteworthy that the other 49 families also contain other disulphide bonds with variable conservation. The fraction of single disulphide bonded families where the disulphide is fully conserved is very less, i.e. 15 out of 111 families (Table 2).

**Table 2:** Characteristics of families possessing a single distinct disulphide that is fully conserved across all members.

**SCOP family code**	**SCOP family name**	**Number of proteins in the family**	**Range of sequence similarity**	**Solvent accessibility**
a.137.2.1	Methanol dehydrogenase subunit	4	65.2–73.7	Exposed
a.152.1.2	Hypothetical protein TM1620	2	100	Exposed
a.33.1.1	Ectatomin subunits	2	47.1	Buried or partially exposed
b.1.18.4	Class II viral fusion proteins C-terminal domain	2	29.3	Buried
b.12.1.2	Colipase-binding domain	4	53.2–83.1	Exposed
b.16.1.1	Ecotin, trypsin inhibitor	2	95.4	Exposed
b.18.1.2	Discoidin domain (FA58C, coagulation factor 5/8 C-terminal domain)	3	38.2–42	Partially exposed
b.2.2.1	Cellulose-binding domain family II	3	29.5	Buried or partially exposed
b.2.3.5	F17c-type adhesin	2	91.4	Partially exposed
b.29.1.5	Pentraxin (pentaxin)	2	51.5	Buried
b.61.2.1	Metalloprotease inhibitor	2	38	Partially exposed
b.78.1.1	alpha-D-mannose-specific plant lectins	5	48.1–85.2	Buried
c.69.1.24	Dipeptidyl peptidase IV/CD26, C-terminal domain	2	92.6	Buried
d.165.1.2	Shiga toxin, A-chain	2	57.3	Partially exposed
d.233.1.1	Inhibitor of vertebrate lysozyme, Ivy	2	19.7	Partially exposed

### Conservation status of disulphide bonds versus sequence identity in pairwise comparisons

**Figure 4:**
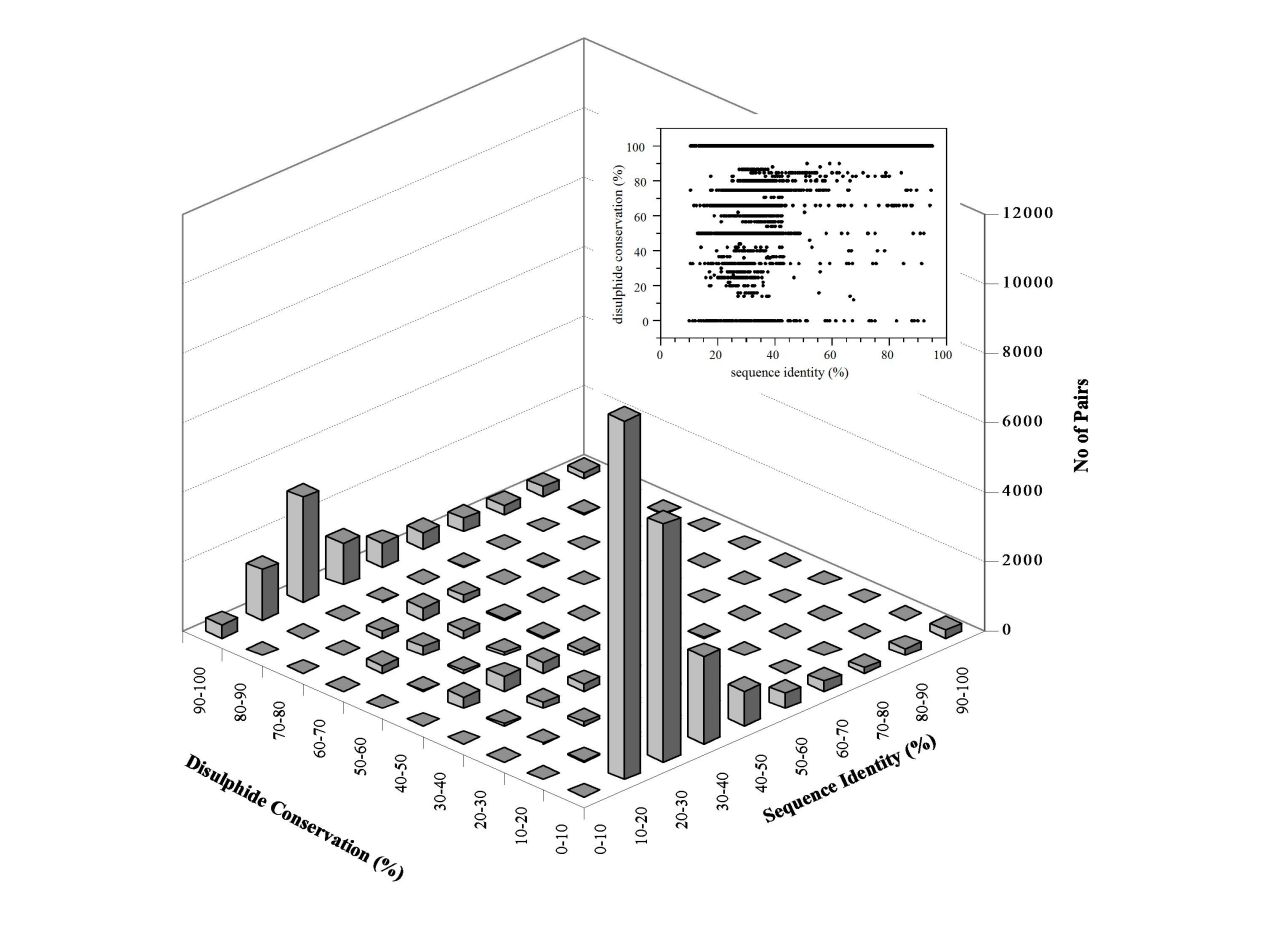
**Disulphide bond conservation plotted against sequence identity for all the pairwise comparisons among the members of disulphide bond containing SCOP structural families**. A total of 34,752 pairwise comparisons are made between members of the 300 families analysed. A two-dimensional scatter plot is also illustrated in the inset.

During the course of evolution, considerable changes in the amino acid sequences may occur in homologous proteins usually retaining the function. When not functionally relevant, disulphide bonds act as structural scaffolds in small proteins, impose geometrical restraints and reduce entropy during folding, or provide additional stability to flexible regions liable under extreme conditions of pH or temperature. Hence, one might argue that disulphide bonds are strongly conserved in homologous proteins sharing higher sequence similarity. To investigate this relationship, we have plotted pairwise sequence identity against pairwise disulphide bond conservation (Figure. 4).

As demonstrated in the previous sections, non-conservation of disulphides is a common feature though, given the importance of disulphide bonds, one would have expected their good conservation throughout the range of sequence identities. But, as shown in Figure. 4, the spread of the data points in the different disulphide conservation ranges namely beyond the 50% or even above the 75% sequence similarity mark suggests the opposite. This is interesting since the structurally and functionally important features (like disulphides) in closely related proteins may be expected to be well conserved. It should be noted here that care has been taken, when setting up the dataset, to remove engineered or mutant proteins and hence these protein entries with high sequence identity are natural homologues. On the other hand, the entire range of disulphide conservation is densely populated below the 40% sequence identity mark. A high fraction of disulphide conservation at lower sequence similarity points to the selective evolutionary pressure to preserve the fold, whereas non-conservation can be readily attributed to sequence divergence or recruitment of additional structural features. In either case, it clearly emerges that not all disulphide bonds are equally important even in closely related proteins. The highly conserved disulphide bonds are the ones that would play a crucial role in the folding process or function of the protein, while the rest would have protein specific roles.

Disulphide bonds are known to be present mainly in the extracellular proteins and rare in the cytoplasmic compartments of most organisms, due to the reductive nature of the cytosol [54,55]. In bacteria they are usually restricted to extra-cytoplasmic compartments with a more oxidative nature or secreted into the media, and in eukaryota the endoplasmatic reticulum or secreted into the external milieu.

Although rare, there are examples of disulfide bonds in intracellular proteins. They are found mainly in proteins that catalyze oxidation-reduction (redox) processes. For example, in the members of thoiredoxin family, thioredoxin and glutathione reductase, a disulfide bond forms during part of a catalytic cycle, and Hsp33 and OxyR, in which a disulfide bond forms as part of a redox-sensing mechanism [56-58]. These functionally important disulphide bonds are usually very highly conserved even as the family members share very low sequence identity (Figure 4 and above discussion). But such intracellular disulfide bonds are rare and generally transiently formed or marginally stable, rather than being essential for structural integrity.

However an exception to this rule is the unexpected discovery of three intra-chain disulfide bonds in archeal adenylosuccinate lyase (ASL) from *Pyrobaculum aerophilum*[59]. These disulfide bonds are poorly conserved and the absence of these disulfide bonds in ASLs from other species implies that the *P. aerophilum *enzyme acquired (or retained) its disulfide bonds during evolution to increase its stability to extreme environmental conditions. Similarly, the disulphide bond in C-terminal domain TATA-box binding protein from Archaeon *Pyrococcus woesei *(SCOP family code: d.129.1.1) is absent in other species.

The lack of correlation between disulphide conservation and sequence identity, however, raises another interesting question *i.e*. is there a relationship between the extent of conservation of disulphide bonds and disulphide richness? Richness or abundance of disulphides is calculated as the number of distinct disulphides compared between a pair of proteins divided by the average length of their sequences. When plotted, the dispersion of data points across the range of conservation clearly indicated there exists no correlation between conservation and the disulphide richness (data not shown). Hence, from Figure. 4, two distinct situations can be expected: (i) non-conservation of disulphide bonds in homologues with high sequence identity and (ii) high conservation of disulphides in homologues with low sequence identity. With respect to this, we have performed a thorough analysis of several families of proteins and collected literature to understand these two situations.

(i) non-conservation of disulphides in homologues with high sequence identity

In the current analysis, several cases of homologues with high sequence identity but with non-conservation of disulphide bonds have been observed. We specifically studied several such cases to understand the reasons for their absence/appearance. The reasons are presented below and in Table 3.

**Table 3:** Reasons for non-conservation of disulphide bonds between homologous proteins that share high sequence similarity.

**SCOP Family**	**PDB code of Proteins**		**Cysteine positions of the SS bond**	**Sequence Similarity** **%**	**Reason for non-conservation**	**Comment**	**Ref**
						
	**with SS**	**without SS**					
Rubisco, large subunit (c.1.14.1)	3rub	8ruc	172–192	94.7	Crystallographic artifact	Not clear if functionally significant or formed during crystallization period	[83]
Purple acid phosphatase (d.159.1.1)	1ute	1qhw	163–221	84.7	Crystallographic artifact	No indication crosslink in electron density map	[84]
VHS domain (a.118.9.2)	1ujk	1juq	33–76	72.7	Crystallographic artifact	Not favorable to accommodate cross link	-
DnaQ-like 3'-5' exonuclase (c.55.3.5)	1noy	1ih7	41–55	63.9	Crystallographic artifact	Not favorable to accommodate cross link	-
Transferrin (c.94.1.2)	-	1gv8 2tmp	-	-	Fragments of domains	1gv8 – fragment of N-terminal domain of the intact protein, ovotransferrin 2tmp – only 1–122 residues of N-terminal domain of TIMP	[85]
Annexin (a.65.1.1)	-	1scf	-	-	Fragments of domains	Partial structure	-
G proteins (c.37.1.8)	-	1ryh 1mh1 1dsb	-	-	Fragments of domains	Partial structure	-
Glutathione S-transferase, N-terminal domain (c.47.1.5)	1k0m	1rk4	24–59	98.6	Structural transition	A typical glutathione S-transferase but with a glutaredoxin-like active site. Disulphide bond facilitates a redox-controlled structural transition from monomeric to dimeric state	[49]
Prion-like (d.6.1.1)	1i4m	1uw3	179–214	91.2	Structural transition	Rearrangement of disulphide bonds helps in conformationallly altering the prion protein to pathogenic oligomeric form.	[50]
Alpha-macroglobulin receptor domain (b.2.4.1)	1ayo/1bv8	1edy	17–132	66.2	Structural transition	Major conformational differences between human/bovine and rat RBD	[60]
Papain-like (d.3.1.1)	1qdq	3pbh	148–252	88.9	Stabilization of local structure	Disulphide bond increases the conformational flexibility of the occluding loop, although the conformational stability of the overall structure is little affected.	[54]
Parvalbumin (a.39.1.4)	1a75	1bu3	11–33	88.7	Stabilization of local structure	This disulphide bond is unique to Whiting parvalbumin and stabilizes the two helical hairpin although the conformational stability of the overall structure is little affected.	-
Dipeptidyl peptidase IV/CD26, N-terminal domain (b.70.3.1)	1nu6	1orv	328–339	85.7	Increased activity	Adenosine deaminase (ADA) binds stronger to the disulphide bonded human protein than in porcine.	[51]
Ricin B-like (b.42.2.1)	2aai	1m2t 1onk	20–39	72.6	Increased activity	Reduced cytotoxicity in mistle toe lectin	[52]
Xylanase/endoglucanase 11/12 (b.29.1.11)	1yna	1xnd	110–154	59.5	Increased activity	Increased thermostability due to compounding effect of disulphide bond with increase in the density of charged particles	[53]
Mycobacterial antigens (c.69.1.3)	1f0n	1dqz	87–92	72.1	Unassigned role	Not obvious from the structural differences if the antigens have different biological roles	[55]
Quinoprotein alcohol dehydrogenase-like (b.70.1.1)	1g72	1kb0 1kb9	144–167	33.2	Unassigned role	No structural or functional role assigned	[56]
Subtilases (c.41.1.1)	1dbi	1thm	137–139	61.6	Unassigned role	C-X-C disulphide bridge is hypothesised to enhance the thermaostability	[57]
Hemorrhagin (d.92.1.9)	1bud	4aig	157–164	49.2	Unassigned role	No direct evidence if the variable disulphide bridges in the C-terminal subdomain hemorrhagin family of enzymes lead to differences in their hemorrhagic activity.	[58]

We have identified several naturally occurring non-conserved disulphide bonds in close homologues, which may play a significant role in their structural or functional differences. These disulphide bonds significantly contribute towards the protein structure or function (see Table 3) through oligomerization[60,61] or increased activity [62-64] or by bringing about local structural compaction[65] yet with little effect on the conformational stability of the overall fold. These disulphide bonds provide local structural stabilization usually in conjunction with other features like clustering of hydrophobic residues.

It is, however, not always immediately possible to ascertain role of such disulphides in related proteins [66-69]. An additional disulphide bond may not have direct effect on the functional difference between the homologues, although it brings about local structural variation.

(ii) high conservation with low sequence identity

Thioltransferases (c.47.1.1) are the members of thioredoxin superfamily, with a highly conserved fold and two vicinal (CXXC) active site cysteine residues and mediate formation of disulphides (oxidized form) or dithiols (reduced form). This class of enzymes mediates oxidation and reduction of disulphide bonds through thiol-disulfide exchange reactions between the active site cysteines of these enzymes and the cysteines in the target protein. Some of the members of this family, for example, thiredoxin from *Anabaena *sp. (PDB id: 1thx) and glutaredoxin from pig (PDB id: 1kte) share 9.5% sequence identity, while spinach (PDB id: 1f9m) and *Escherichia coli *(PDB id: 1fov) thioredoxins share only 9.8% sequence identity. This conservation is attributed to the functional role of the disulphide bond for the catalysis of thiol-disulphide exchange reactions required for many functions including electron and proton transport to essential enzymes like ribonucleotide reductase, for the formation of disulphides in protein folding and for general regulation of protein function by thiol-redox control. On the other hand, the density, distribution and connectivity patterns of disulphide bonds in many small disulphide-rich proteins impart regularities to their structures and play a major role in determining their fold even in the absence of clear sequence similarity. The three disulphide bonds in the family of small Kunitz-type inhibitors and BPTI-like proteins (SCOP family code: g.8.1.1) are highly conserved with their sequence similarity in the range of 28.6 to 95.6%. The members of this family, along with soft tick anticoagulant proteins (SCOP family code: g.8.1.2) classified under BPTI-like superfamily in SCOP, can be clustered into a single group based on the disulphide bonding pattern[28] while the sequence similarity across these families is as low as less than 20%.

In SCOP classification, sequence identity is not a lone criterion to cluster families of proteins[42]. When established by similar functions and structures, proteins are grouped together into same families even if their sequence identities are below 30%. The members of plant defensin family (SCOP family code: g.3.7.5) share four highly conserved disulphide bonds with a sequence similarity sometimes as low as 13.3%. Similarly, ATX Ia (a neurotoxin) and BDS-I (an antiviral protein) do not share any observable sequence similarity but share three highly conserved disulphide bonds[70].

### Interactions among the substituted residues

When a disulphide is not conserved, the local interactions among the substituted residues in the homologues lacking disulphide evoke some interest. Hence, to understand the functional implications of this non-conservation, we evaluated the possible interactions among the substituted residues lying in topologically conserved regions of the family of proteins.

In all, we have observed 7064 substitutions out of which, as expected, 87% are accounting for low or poorly conserved disulphide bonds. In about 25% of these poorly conserved disulphides, the bonded cysteines are aligned with gaps in the alignment that was derived from structural superimposition. Another 35% have at least one cysteine residues aligned with a gap. These depict situations where disulphides do not share topologically equivalent counterparts. Otherwise, in only 3% of the situations, both cysteines are aligned with residues in topologically conserved regions. These substituted residues can undergo, at best, non-covalent interactions and rarely covalent interactions (e.g. transglutamination), if any. Although in principle all interactions are electrostatic in nature, hydrogen bonding, hydrophobic interactions, aromatic and aliphatic-aromatic interactions were investigated separately.

A total of 54 distinct disulphides distributed in 43 families featured 213 topologically equivalent substitutions. These substitutions were studied with respect to the conservation status (highly or poorly conserved) of their native distinct disulphide. These results are featured in additional file 1. In order to avoid any bias inherent to our dataset, we further analysed the interactions at 40% sequence identity. The same general trend was observed.

It appears from our data that, in cases where the disulphides are generally highly conserved in the family (23 out of 54 disulphides), in the members that lack these disulphide bonds, there is a clear preference for hydrophobic residues among substituted residues (Additional file 1). Several of these disulphide substitutions cases are well documented in the literature. Examples are the Phe-Phe interaction that stabilises the monomers of α_2_M rat receptor binding domain (PDB id: 1edy) by bringing their amino- and carboxy-termini together[71]; the π-conjugation type interaction between Tyr25 and Ala106 residues in the anion binding site of YBHB from *E. coli *hence tightening the loops GXG and CR2 (a conserved motif) to each other[72], the aromatic-alphatic interaction between Trp-48 and Leu-65 in BphF, a Rieske-type ferredoxin associated with biphenyl dioxygenase (PDB id: 1fqt), which importantly contributes to lower the redox midpoint potential of the protein by approximately 500 mV[73].

On the other hand, the substitutions at positions of poorly conserved disulphide bonds (31 out of 54 distinct disulphides) correspond to residues that are not congenial for favourable inter-residue interactions (Additional file 1). A typical case is illustrated by adenylosuccinate lyase (SCOP family code: a.127.1.1) from the three disulphide bonded hyperthermophilic *Pyrobaculum aerophilum *and its homologue from *Thermotoga maritima *where the absence of three disulphides is compensated through other sequence and structural features within the vicinity of the disulphide bond of these proteins but not directly by the substituted residues[59]. Where a disulphide bond is absent, interactions among stabilization center residues, clusters of residues in protein contact maps where an accumulation of long range interactions are observed [74,75], also compensate for the absence of disulphide. Other typical cases are further documented in Additional files.

### Backbone conformation and residue length separating the half-cystines and conservation

**Figure 5:**
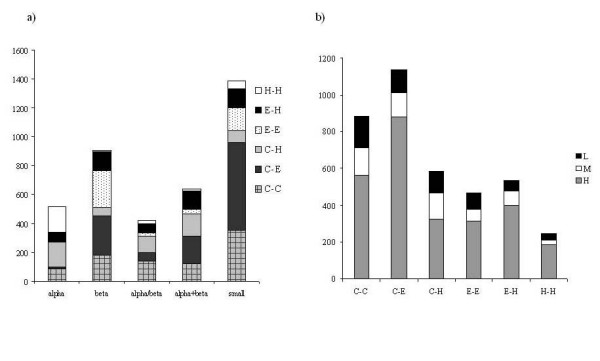
**Fold distribution of different backbone conformations of disulphide bonds: Alpha, beta, alpha/beta, alpha+beta, and small proteins are different SCOP fold classes; C/E/H are coil/strand/helical conformations of the half-cystines**. a) Backbone conformational preferences of disulphide bonds in different fold classes, b) Distribution of disulphide bond backbone conformations into different conservation classes; L-low or poorly conserved, M-medium conserved, and H-highly conserved.

Figure 5 represents the secondary structure at the two half-cystines of disulphide bonds in the present data set. It can be seen that the disulphide bonded cysteines correspond predominantly to coil-coil and coil-strand connectivities. Figure. 5 also depicts the secondary structure preferential distribution of half-cystines in different fold classes. It can be seen that the disulphide secondary structure connectivity preference is not biased by the small proteins which is the most populated protein class with a total of 1385 disulphides and by the fact that small proteins have a large preference (77%) for half-cystines to connect a coil region.

It is expected that local structures of the half-cystine containing regions would be topologically conserved in homologous proteins along with the conservation of the disulphide bond itself. As previously shown (cf *supra*), in about 70% of the disulphide bonds, at least one of the two half-cystines is structurally conserved. This is rather interesting since more than 68% of disulphide bonds in our data set occur in loop regions or connect regular secondary structures with coiled regions. Hence, the highly variable nature of loops/coils did not deter the disulphide bond conservation, strongly suggesting their role in structural stabilization (Figure. 5). On the other hand, in almost 80% of the non-conserved disulphide bonds, the secondary structure status of at least one half-cystine is in coil state. This suggests that disulphide bonds bring rigidity to such structurally variable regions in homologous proteins. In general, neither highly conserved nor low conserved disulphides display a preference for a particular local backbone conformation.

**Figure 6:**
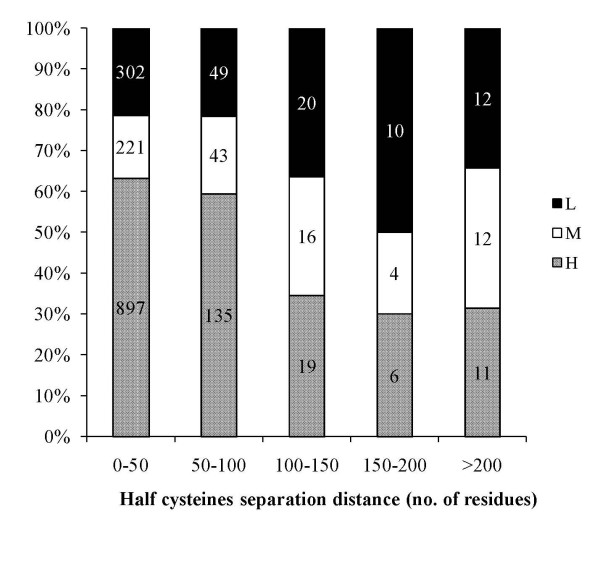
**Conservation status (H, M or L) of disulphide bonds as a function of half-cystine separation distance**.

An overwhelming majority (97%) of the disulphide bonds studied in our dataset have their half-cystines separated by less then 50 residues. A subset of our initial data, culled at 40% sequence identity, has been analysed by dividing them into five groups with increasing loop sizes of 50. Figure. 6 shows the probabilities for these groups to be highly or poorly conserved, indicates clear trends. A majority of the poorly conserved disulphides (i.e. nearly 75%) are non-local (> 100 residue separation) in the current analysis. Similarly, about 40% of non-local disulphides are poorly conserved and this drops to 20% in the case of local disulphides. Small proteins did not bias this observation since the data was analysed with and without the small proteins. Non-local disulphide bonds are less frequent and are not as much important as local disulphides which helps in kinetic folding by bringing together local regions of the protein [76].

### Solvent accessibility of disulphides and conservation

Since the relationship between extent of conservation of disulphides and their solvent accessibility is not yet known, we performed a survey of the solvent burial of disulphides in our dataset and integrated the information on extent of disulphide conservation. In this analysis, disulphide bond burial has been defined as follows: when both half-cystines are buried, the disulphide has been considered as 'buried'; when one half-cystine is buried and the other solvent exposed, the disulphide is considered as 'partially exposed'; when both half-cystines are solvent accessible, such disulphides are considered as 'exposed'.

**Figure 7:**
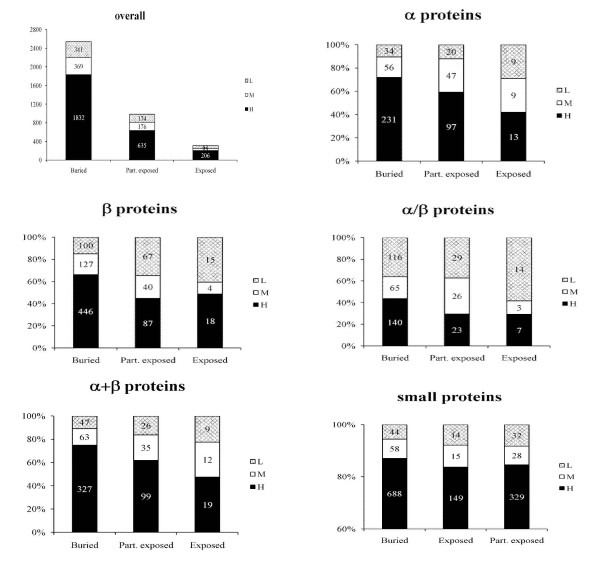
**Analysis of solvent accessibility (buried, partially exposed and exposed) of disulphide bonds and their conservation status (H, M and L) with regard to the SCOP classification of proteins**.

From Figure 7 and further analysis of the data it can be inferred that overall, buried as well as fully exposed disulphides share the same tendency (66–72%) for conservation. It should be noted that nearly 44% of the exposed half-cystines belong to small proteins, which can be reasoned by the lack of definite hydrophobic core as in globular proteins, thus emphasising their importance in maintaining the fold through the structural scaffold. However, it is noteworthy that buried as well as exposed disulphides are also vulnerable to mutations with as much as 13% and 19% respectively that are low conserved. Our results hence seem to establish that there is no apparent preference between high conservation and the solvent accessibility status of disulphides, i.e. high conservation of a specific disulphide can be an important feature of the disulphide irrespective of whether it is buried or exposed. Our results also suggest that there is no preference for poorly conserved disulphides to be exposed or buried. Similarly, our data shows that disulphide exposure would not necessarily be associated with low conservation.

However, when a distinction between small proteins and bigger proteins is made (Figure. 7), a higher proportion of partially or fully exposed disulphides are poorly or medium conserved. Though very slight, there is a general tendency in bigger proteins for exposed disulphides to be less conserved than buried disulphides. This is particularly clear in α/β proteins. From 58 α/β protein families containing 427 disulphides about 70% of the partially or fully exposed disulphides are low or medium conserved. On the other hand, high conservation of disulphides is common in small proteins and it is independent of the status of their disulphide solvent accessibilities (Figure. 7, also see Figure. 2 for distribution of disulphide bonds in different fold classes).

### Stereochemistry versus conservation of disulphide bonds

Analysis of disulphide bridge stereochemistry might give clues if there exists a relation between geometry and conservation of disulphide bonds. The bridge stereochemical parameters are calculated for all the native disulphide bonds in the database and are graded according to the criteria defined by Sowdhamini and co-workers [26,77]. A native disulphide bond, irrespective of its level of conservation, tends to appear in ideal stereochemistry (data not shown).

To perform this analysis, we selected a subset of protein structural data of high quality (571 disulphide bonds from 172 proteins) of our original data, with a crystallographic resolution better than 2 Å and R-factor 0.25 and sequence identity between homologues less than 25%.

**Figure 8:**
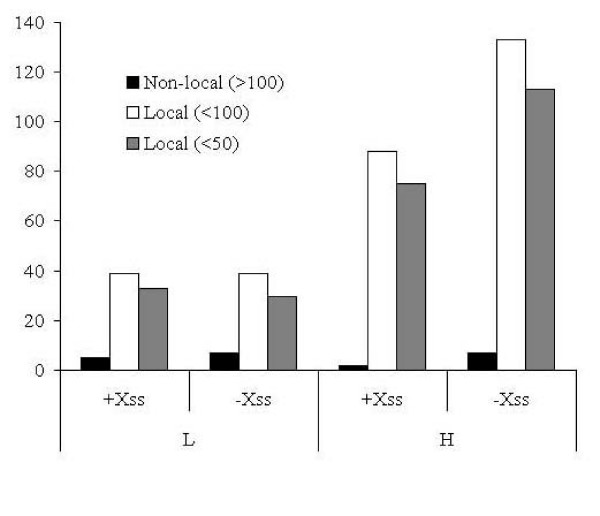
**Preferences for handedness of disulphide bonds in highly (H) and poorly (L) conserved disulphide bonds with respect to their Chi angle of the SS bond. -ve left-handed; +ve right-handed**.

The distribution of Cα and Cβ distances in the well and poorly conserved classes of disulphide bonds are very similar and did not show any significant deviation from ideal values (data not shown). Similarly, the SS bond dihedral angle showed similar distribution patterns. Although poorly conserved disulphide bonds are not necessarily non-local, as shown in previous section, several non-local disulphide bonds are poorly conserved. It would hence be of interest to see if the poorly conserved non-local disulphide bonds are strained or show deviations. Interestingly, contrary to the observation of Thornton[2] based on limited data that local disulphide bonds tend to be right handed, we observed a marginal preference to be left handed in highly conserved disulphide bonds. Low or poorly conserved disulphide bonds distributed equally in left handed and right handed conformations. However, non-local disulphide bonds, although sparse, indeed showed slightly higher preference to be left-handed (Figure. 8).

The distribution of all the bridge torsion angles (χss, χ1, χ2) of both highly and poorly conserved disulphide bonds were plotted and the respective histograms were very similar and also showed close similarity with previous studies (data not shown)[78]. Stereochemical evaluation necessitates studies of the specific families, since stereochemistry can be influenced by the local sequence variations of the homologues.

Overall, our results suggest that extent of conservation of a disulphide is independent of the conformation of the bridge.

### Variations of disulphides at family and superfamily level

As stated in the previous sections, non-conservation of distinct disulphide bonds within homologous families is a common feature and we analysed its biological implications through several examples. Such an analysis can be extended by investigating the conservation and structural variations of disulphides between members within a homologous family and across families in a superfamily (see Additional file 2 for a discussion on limited number of examples and Additional files 3, 4, and 5 for associated figures). This analysis, though limited to a superfamily, illustrates non-conserved disulphide bonds across superfamily members have limited effect on their common fold, but could be associated with recruitment of new structural features.

## Discussion

The purpose of this study is to examine the question of the extent of conservation of disulphide bonds in homologous proteins. The whole study is performed from a structural point of view so that we do not have to compensate for the quality of alignments nor make any assumptions on disulphide bond connectivity after including sequence homologues. For long, disulphide bonds are seen as conserved features like enzyme active sites, and often used to draw relations between distant relatives. There has been no general or systematic study previously on the structural conservation of disulphide bonds amongst a large number of protein domain families, although several protein specific studies highlighted the presence or absence of an extra disulphide bond in close homologues. The availability of classified information in terms of homologous protein families with sufficient number of structural entries has prompted us to carry out a large-scale analysis to quantify the extent of conservation of disulphide bonds.

The distribution of these mutations of disulphides is widespread in all structural classes and grading the extent of conservation enabled in quickly identifying the variable crosslinks. We found that the discriminative ability of such a large-scale analysis is high, helps in quickly identifying highly conserved disulphide bonds in homologues, which might play a crucial role in protein folding or maintaining the fold. Almost 60% of substitutions of poorly conserved disulphides in our dataset are gaps, suggesting that the impact of such disulphide bonds on protein folding kinetics could possibly be limited. For example, disulphide bonds in or near the folding nucleus enhance folding and are usually conserved in related proteins. Disulphides outside the folding nucleus nevertheless have different functions like in extracellular proteases (Rossmann fold containing proteins) where they protect a cell against errors in translocation and undesirable protease activity while they are in cytoplasm[30].

Protein structures diverge linearly, although at a much lower rate, with the sequence variability in homologous proteins [79,80]. However, several families of proteins, involving distant relatives, withstand such an evolutionary pressure and show minimal or no correlation between sequence and structure variation. While this could be due to the strong functional constraints like catalytic site conformation[81], disulphide bonds stabilizing the protein core form the structural constraints[79]. Our approach to study disulphide conservation holds the information on structural evolution of homologous proteins since it inherently encompasses the sequence-structure dependence. Hence, the invariance of disulphide bond conservation with the sequence divergence (see Figure. 4) can be reasoned to selection pressure, preserving these bonds due to their strong structural/functional role. We also found only in less than 15% single disulphide bonded families the crosslink is highly conserved. Similarly, abundance of disulphide (number of disulphides in a protein) has little effect on conservation. This suggests there are other factors that influence the sequence-structure relationship and there is no relation between the number of disulphide bonds and their conservation. Hence, conservation of disulphides can be reasonably explained by the important implications of disulphides in the structures and/or functions of proteins.

Disulphide bonds playing a functional role are usually highly conserved, where as disulphides in structurally similar but functionally different proteins are poorly conserved. In fact, disulphides probably have varying degree of conservation among homologous proteins depending upon if a given disulphide have structural or folding or functional or regulatory role. Sometimes a given disulphide might play more than one role. In practice such an analysis is very difficult to perform as detailed knowledge on structural/folding/functional/regulatory roles of most disulphides in most proteins is not available.

We show that non-conservation of disulphides within protein families can be associated with substitutions displaying favourable interactions or with other subtle structural changes that compensate the loss of covalent link in a homologue compared to another. Disulphide bond mutation not always changes the basic structure of the protein[2], but might affect the activity of the protein without significantly changing the structure[82,83], sometimes even without altering the function[39,84]. Our work also shows that non-conserved disulphides are often associated with variable structural features that were recruited during evolution and this recruitment is generally associated with differentiation or specialisation of protein function. One of the major implications of the non-conservation of disulphides within homologous families is that it confers plasticity to disulphide connectivity patterns. Our study clearly re-establishes that this plasticity is correlated to structural or functional constraints. Conserved disulphides result in conservation of their patterns, although with a few exceptions (conserved topology but not the connectivity pattern). The common or mixed ancestry of diverse proteins can be recognized by conservation of disulphide bonds[28,29,70] even when most of the surrounding residues have been replaced[85]. Attempts were made to map the patterns of paired natural cysteine mutations[41]. It has been shown that cystine mutations are coordinated and results in the loss of the disulphide bond[2] and such a feature can be used to identify the connectivity of cystines in multiple disulphide bonded families[41].

It has been noted that remote homology detection using disulphide bond connectivity, is severely limited not only by the requirement of their natural occurrence [28,70], but also by the lack of information on how far one can relax the size of the disulphide loops during searches. The availability of a library of disulphide profiles in the future might facilitate associating proteins with new connectivity and for the rational design of site-directed mutagenesis experiments.

We clearly demonstrate here that conservation of disulphides is a family-specific feature and is neither correlated to solvent accessibility status nor to backbone conformation. While disulphide bonds are common in all fold classes, their preference for stabilizing strands and loops has concentrated their presence in beta-strand class and in small proteins. Also the extracellular or secreted proteins, where disulphide bonds almost exclusively appear with some exceptions [86,87], usually adopt β and α+β type structures having more beta structures stabilized in part by medium- and long-range non-covalent interactions apart from disulphide bonds [74,88,89]. Hence the secondary structural preferences of extracellular or secreted proteins and streochemical restraints at the backbone regions where disulphide bonds are accommodated appear to be inter-related. The evolutionary plasticity of loop regions is greater than that of protein core [79] and this would explain the greater crosslink frequency in the loop regions of homologous proteins and which helps in maintaining the fold.

Interestingly, our work demonstrates that there is no clear correlation between sequence similarity and degree of disulphide conservation. It is shown that high sequence similarity does not guarantee disulphide bond conservation. Genuine cases where highly similar proteins do not share common disulphides are documented. To our knowledge, this is the first documented study that focuses on such a relationship, thus highlighting the singularity of the evolutionary dynamics of disulphides bridges in homologous protein structures. The idea of analysing disulphide bond conservation in protein families has recently been implemented into a web facility freely accessible at [90].

## Methods

### Dataset

The release 1.67 of SCOP [42] containing 2630 families with over 65122 domains has been used in the present analysis. The SCOP database, created by manual inspection and abetted by a battery of automated methods, broadly classifies all proteins in to five major classes, alpha, beta, alpha/beta, alpha+beta and small proteins. Small proteins are usually with less than 70 residues and are rich in disulphide bonds. Disulphide bond definitions of the domains are extracted from the SSBOND record of the corresponding PDB [91] files. Only intra-domain disulphide bonds are considered in the present study.

Of these, 580 families have at least one member with disulphide bond. Ignoring multidomain and membrane classes resulted in 557 families with at least one member with a disulphide bond. The dataset is pruned to remove redundancy (mutant and identical protein entries) rather than close sequence homologues. Filtering the initial dataset at 95% sequence identity and a resolution better than 2.5 Å has resulted in 300 families containing at least two members in each family. For proteins with NMR derived structures, the first model in the ensemble of structures was considered for superimposition.

When we tried to enrich the dataset to include homologous proteins of yet unknown structure, we encountered two problems: Firstly, in the case of distant, yet, definite homologues it is difficult to be confident of good quality of alignment with the homologues as structure is unavailable. Second problem which is even more serious is the non-availability of disulphide bond connectivity established by experimental methods for several sequences. If cysteines involved in disulphide formation in a protein are conserved in a homologue of unknown structure, one can be fairly confident about such disulphide connectivity in the homologue with yet unknown structure. However, if the cysteines occur in unconserved positions, especially in insertions, in the protein of unknown structure then the disulphide connectivity information for the protein of unknown structure is incomplete. Due to these problems we confined our analysis only to proteins of known 3-D structure.

### Cellular localisation of proteins in the dataset

Three prediction methods (TargetP[92], PSORT[93] and SubLoc[94]) and literature curation (Additional file 6) was performed, where possible, on the full-length sequence of the proteins in the entire dataset. Where all three or two out of three methods predict a protein to be cytoplasmic, we have examined the residues at the site of disulphide bond positions in the corresponding family (see Additional files 7 and 8).

### Structural superimposition

Structure-based sequence alignments in these SCOP families were derived using STAMP package[95]. STAMP aligns a set of homologous protein structures and generates a structure-based multiple sequence alignment among others. Topologically equivalent regions usually correspond to conserved secondary structures within a family of structures under comparison. Topologically equivalent regions from the multiple structural alignments are derived by a method of Argos and Rossman, implemented internally in the structural alignment program by calculating the probability of residue structural equivalencies[96]. The block of multiple structure-based sequence alignment is considered as the *alignment footprint *of the family.

### Annotation of distinct disulphide bonds and grading of conservation in a family

**Figure 9:**
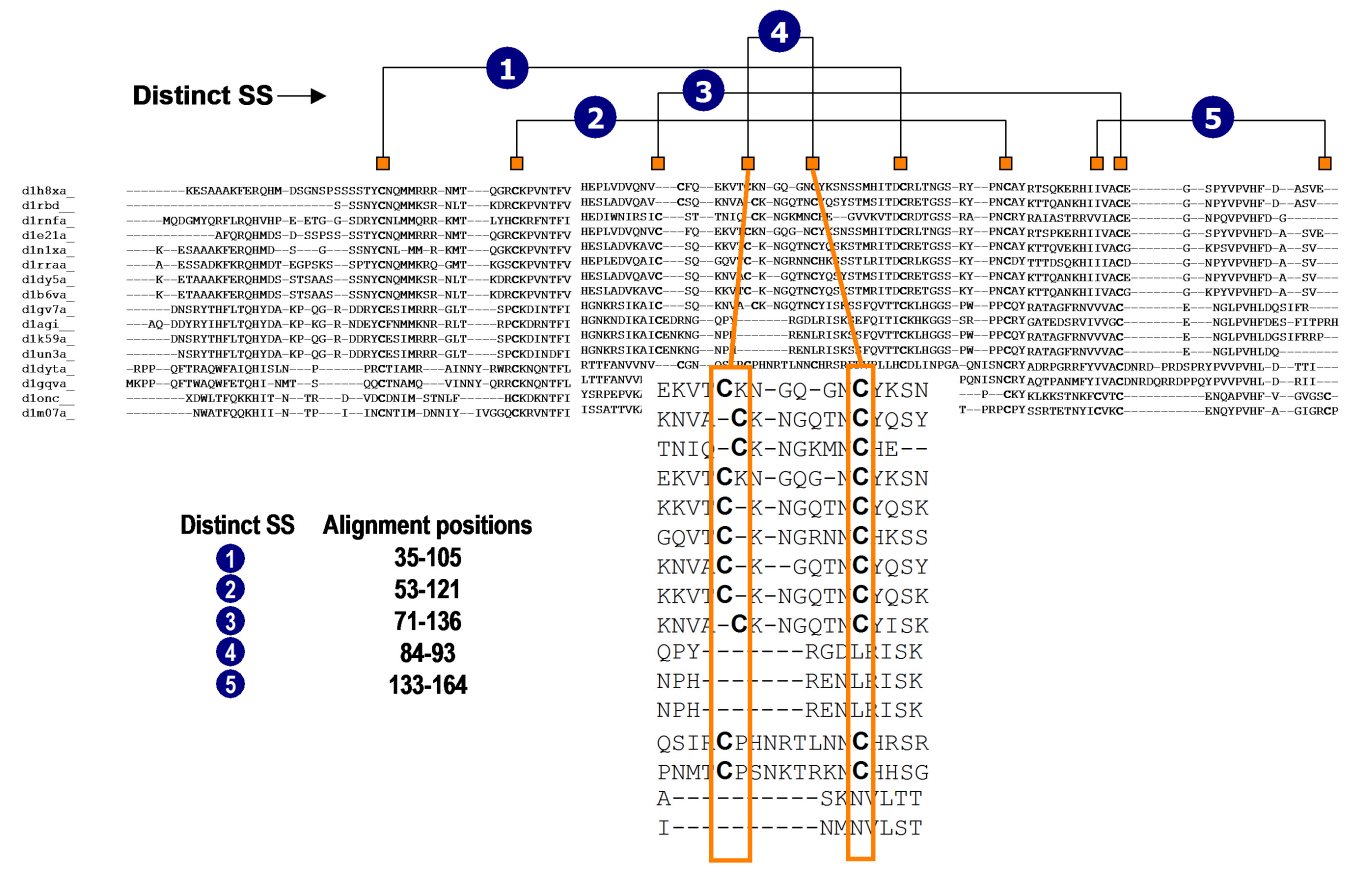
**Illustration of distinct disulphide bond annotation in Ribonuclease A-like [SCOP family code: d.5.1.1] family of proteins**. The structural alignment contains sixteen members. Marking of the disulphide bond positions on the structure based multiple sequence alignment has resulted in five distinct structural positions. However the maximum number of disulphides in a family member is only four, while the minimum is three. Each distinct disulphide bond is represented with approximate positions of their cysteines in the alignment. In the zoom out of the alignment region of the 4th distinct disulphide bond note the first cysteine is not in equivalent position, nevertheless it is conserved. Applying relaxation brings this disulphide bond into conservation. Also notice that this disulphide bond is not conserved in all the members.

Native disulphide bond information has been extracted from the SSBOND record of PDB files of all protein structures. For every family in the data set, the positions of disulphide bonds in homologous members are marked on the structural *alignment footprint *of the family. The positions of the disulphide bonds in this footprint region are counted as distinct disulphide bonds in the family (Figure. 9). Such an annotation is referred in this paper as the '*disulphide profile*' for the family and facilitates a global view of the connectivity pattern of all the disulphide bonds in a family of aligned proteins, irrespective of the presence or absence of a disulphide in the individual members. Once the distinct disulphide bonds in a family are identified, their conservation is calculated as a ratio between the number of proteins containing the distinct disulphide bond and the total number of proteins in the alignment. When there is no disulphide bond found in a member at a position, a relaxation (Figure. 9) has been applied by allowing an arbitrary shift of 4 residues on either side of the cysteine positions for counting the distinct disulphide bonds.

To understand the importance of the evolutionary variations of disulphide bonds in protein families, conservation is quantified and graded. A highly conserved disulphide bond is one that is present in more than 70% of the family members while medium and poorly conserved disulphide bonds are present in 30–70% and less than 30% of the members of the family, respectively.

### Descriptors of structural environment of disulphide bonds

Backbone conformation, solvent accessibility and residue depth are chosen to define the structural environment of the cysteines involved in disulphide bond formation. The backbone conformation and secondary structure assignment of half-cystine are derived using the program, DSSP[97]. However, a three state assignment is used instead of the default seven states. We grouped the eight DSSP labels into three categories: helix, H = 'HGI' (a-helix, 3-helix and p-helix), sheet, E = 'EB' and loop, C = 'TS' (turns and bends). Solvent accessibility has been calculated using the method of Lee and Richards[98] as implemented in the program NACCESS[99]. The relative accessibility of the half-cystine is its percentage solvent accessibility (SA) compared to Ala-Cys-Ala tripeptide. A half cystine with less than 10% SA is considered buried, 10–25% is partially exposed and with more than 25% SA is considered exposed. Residue depth is calculated by using a program, DPX[100]. This program gives the depth of each atom in protein, defined as distance (Å) from the closest solvent accessible atom and calculates the mean residue depth. These depth values are critically dependant on local parameters like protein size, shape and structure. However, it is important to understand if there exists a correlation among solvent accessibility, conservation and depth of half-cystines. Hence the half-cystine in each protein are classified as core, intermediate or surface relative to the residue depth values of other residues in the protein.

## Abbreviations

Å: Angstrom; DSSP: Define Secondary Structure of Proteins; DPX: Depth of each atom in Protein; NACCESS: Accessible Surface Area; NMR: Nuclear Magnetic Resonance; PDB: Protein Data Bank; SCOP: Structural Classification of Proteins; STAMP: Multiple Structure Superimposition Program; SSBOND: Disulphide Bond

## Authors' contributions

RRT developed the algorithm, performed data analysis and provided draft of the manuscript. MM conducted literature survey on the sub-cellular localization. NS, RS and BO conceived and formulated the ideas and developed the strategies. FC designed the project, provided the overall plan and supervised the project.

## Supplementary Material

Additional file 1 **Nature of side chains of of substituted residues topologically equivalent with cystines of a distinct disulphide bond in their respective families.** For domains where the original disulphide bond is mutated, the topologically equivalent substituted residues are extracted to verify if the interaction is conserved. Click here for file

Additional file 2 **Evolutionary dynamics of disulphides at family and superfamily level.** We extended our analysis by investigating the evolutionary dynamics of disulphides between members within a homologous family and also across families in a superfamily in order to provide further insights into the biological implications of conservation and non-conservation of disulphides. Click here for file

Additional file 3 **Evolutionary dynamics of disulphides in the alpha/beta hydrolase actetylcholinesterase-like family (SCOP family code: c.69.1.1).** Three highly superimposable members from this family are featured. In (a) is featured a representative (SCOP domain: d2bce__; PDB code: 2bce) from the cholesterol esterase and carboxyesterase types which display two highly conserved disulphides. In (b) is featured a representative (SCOP domain: d1dx4a_; PDB code: 1dx4) from the acetylcholinesterase type which display in addition to the two conserved disulphide, a third specific disulphide (arrow). In (c) is featured a representative from the paranitrobenzyl esterases group which do not feature any disulphide though two cysteines which are topologically equivalent to one highly conserved disulphide is present in the molecule. Click here for file

Additional file 4 **Evolutionary dynamics of disulphides in the alpha/beta hydrolase serine carboxypeptidase-like family (SCOP code: c.69.1.5).** Three superimposable members from this family are featured and which display one highly conserved disulphide (arrow). In (a) is featured a representative (SCOP domain: d1ac5_; PDB: 1ac5) from the serine carboxypeptidase II type and which displays two non-conserved disulphides. In (b) is featured a representative (SCOP domain: d1cpya_; PDB: 1cpy) from the human carboxypeptidase L type which display in addition to the conserved disulphide, four specific disulphides. In (c) is featured a representative from the human protective protein group which display in addition to the conserved disulphide, three specific disulphides. Click here for file

Additional file 5 **Representation of Type-B carboxylesterase/lipase (SCOP domain: d1thga_; PDB: **1thg**) from the fungal lipase family (SCOP family code: c.69.1.17).** Representation of Type-B carboxylesterase/lipase (SCOP domain: d1thga_; PDB: 1thg) from the fungal lipase family (SCOP family code: c.69.1.17) that features two disulphides that are topologically equivalent to the two highly conserved disulphides from acetylcholinesterase-like family (SCOP family code: c.69.1.1). Click here for file

Additional file 6 **Localization predictions by the three programs and the consensus starting from full-length sequences of proteins in the dataset.** This table provides a comparison and consensus obtained by applying the three prediction servers for cellular localization. Click here for file

Additional file 7 **Literature survey of proteins highly predicted to be cytoplasmic by all three programs.** This table provides the results of literature survey on comments of cellular localization for the protein chains, that are predicted to be cytoplasmic, by all three programs or by any two out of three programs. Click here for file

Additional file 8 **Conservation of disulphide bonds in protein chains highly predicted as cytoplasmic.** This table provides the results of examination of conservation (or otherwise) of distinct disulphides for proteins that have been highly predicted to be cytoplasmic. Click here for file
