# Supplementary material for: Analysis on conservation of disulphide bonds and their structural features in homologous protein domain families
Source: BMC Struct Biol. 2008 Dec 26;8:55. doi: 10.1186/1472-6807-8-55 (PMC2628669; doi:10.1186/1472-6807-8-55)
Supplement: Additional file 1 — Nature of side chains of of substituted residues topologically equivalent with cystines of a distinct disulphide bond in their respective families. For domains where the original disulphide bond is mutated, the topologically equivalent substituted residues are extracted to verify if the interaction is conserved. [file 1472-6807-8-55-S1.doc]

Table.S1. Interactions of substituted residues topologically equivalent with cystines of a distinct disulphide bond in their respective families.

| **SCOP family name** | **SCOP code** | **SCOP domain** | **Distinct disulphide bond^a^** | | **Substituted residues^b^** | **Original residue positions^c^** | | **Interaction^d^** |
| --- | --- | --- | --- | --- | --- | --- | --- | --- |
| **High or Medium conserved** |  |  |  |  |  |  |  |  |
| Haloperoxidase (bromoperoxidase) | a.111.1.2 | d1up8a_ | 90 | 99 | A/I | 82 | 91 | H |
|  |  | d1up8a_ | 534 | 557 | E/T | 506 | 528 | Hy |
| Fertilization protein | a.19.1.1 | d2lisa_ | 63 | 138 | W/V | 59 | 128 | H/SP-H |
| CCP-like | a.93.1.1 | d1jdra_ | 80 | 102 | S/Y | 54 | 67 | A-Ar |
|  |  | d1aru__ |  |  | A/A | 50 | 68 | H |
|  |  | d1oafa_ |  |  | A/P | 44 | 56 | H |
| Pilus chaperone | b.1.11.1 | d1qpxa1 | 95 | 119 | N/K | 89 | 101 | Hy |
| Peptidylglycine alpha-hydroxylating monooxygenase, PHM | b.121.1.2 | d1sdwa1 | 132 | 155 | V/S | 127 | 143 | H-P |
| Prokaryotic PEBP-like proteins | b.17.1.2 | d1fjja_ | 26 | 109 | Y/A | 24 | 105 | H/SP-H |
| CBM4/9 | b.18.1.14 | d1guia_ | 72 | 185 | Y/Y | 48 | 146 | Ar |
|  |  | d1k45a_ | 72 | 185 | A/Y | 58 | 158 | H-H/SP |
| Amine oxidase catalytic domain | b.30.2.1 | d1oaca1 | 119 | 145 | A/A | 102 | 128 | H |
| Rieske iron-sulfur protein (ISP) | b.33.1.1 | d1fqta_ | 150 | 178 | W/L | 48 | 65 | H/SP-H |
| YWTD domain | b.68.5.1 | d1ijqa1 | 182 | 193 | Y/S | 171 | 182 | A-Ar |
| Quinohemoprotein amine dehydrogenase B chain | b.69.2.2 | d1pbyb_ | 34 | 73 | I/G | 27 | 66 | H |
| Quinoprotein alcohol dehydrogenase-like | b.70.1.1 | d1kb0a2 | 414 | 443 | V/P | 386 | 415 | H |
| Dipeptidyl peptidase IV/CD26, N-terminal domain | b.70.3.1 | d1orva1 | 290 | 301 | I/I | 289 | 300 | H |
| Galacturonase | b.80.1.3 | d1bhe__ | 270 | 293 | N/F | 225 | 247 | A-Ar |
| N-acetylglucosamine 1-phosphate uridyltransferase GlmU, C-terminal domain | b.81.1.4 | d1g97a1 | 56 | 73 | A/V | 56 | 72 | H |
| LexA-related | b.87.1.1 | d1jhfa2 | 117 | 124 | V/F | 107 | 114 | H |
|  |  | d1umua_ |  |  | I/L | 86 | 92 | H |
| DsbA-like | c.47.1.13 | d1r4wa_ | 120 | 123 | S/S | 15 | 18 | Hy |
| Acetylcholinesterase-like | c.69.1.1 | d1mx1a_ | 468 | 600 | V/V | 407 | 523 | H |
|  |  | d2bce__ |  |  | I/L | 388 | 512 | H |
|  |  | d1qe3a_ |  |  | R/R | 355 | 463 | P |
| Arylsulfatase | c.76.1.2 | d1auk__ | 92 | 127 | G/F | 80 | 115 | H |
|  |  | d1hdha_ |  |  | H/Q | 86 | 119 | Hy |
| STAR domain | d.129.3.2 | d1jssa_ | 71 | 225 | D/L | 54 | 198 | P-H |
| L-aminoacid/polyamine oxidase | d.16.1.5 | d1b37a2 | 12 | 144 | F/A | 12 | 92 | H |
|  |  | d1s3ea2 |  |  | Y/P | 12 | 91 | H/SP-H |
|  |  | d1pj5a3 |  |  | T/I | 11 | 108 | P-H |
| MHC antigen-recognition domain | d.19.1.1 | d1uvqa2 | 142 | 222 | Y/A | 15 | 68 | H/SP-H |
| TolA | d.212.1.1 | d1lr0a_ | 53 | 80 | V/D | 51 | 77 | H-P |
| Merozoite surface protein 1 (MSP-1) | g.3.11.4 | d1b9wa1 | 14 | 34 | V/W | 12 | 28 | H-H/SP |
| **Poorly conserved** |  |  |  |  |  |  |  |  |
| L-aspartase/fumarase | a.127.1.1 | d1c3ca_ | 59 | 72 | R/T | 31 | 44 | Hy |
|  |  | d1k7wa_ |  |  | K/L | 31 | 44 | P-H |
|  |  | d1q5na_ |  |  | Q/A | 34 | 47 | P-H |
|  |  | d1vdka_ |  |  | R/A | 55 | 68 | P-H |
| Retrovirus capsid protein C-terminal domain | a.28.3.1 | d1eoqa_ | 53 | 76 | I/V | 48 | 69 | H |
| I set domains | b.1.1.4 | d1epfa1 | 77 | 217 | F/T | 19 | 61 | A-Ar |
|  |  | d1f2qa2 |  |  | F/N | 19 | 50 | A-Ar |
|  |  | d1fltx_ |  |  | V/G | 24 | 60 | H |
|  |  | d1iam_2 |  |  | L/V | 18 | 51 | H |
|  |  | d1epfa2 |  |  | V/Y | 20 | 57 | H-H/SP |
|  |  | d1f42a1 |  |  | V/T | 25 | 52 | H-P |
|  |  | d1f97a2 |  |  | V/D | 21 | 63 | H-P |
|  |  | d1gsma2 |  |  | A/T | 21 | 74 | H-P |

Table.S1 (continued)

| **SCOP family name** | **SCOP code** | **SCOP domain** | **Distinct disulphide bond^a^** | | **Substituted residues^b^** | **Original residue positions^c^** | | **Possible interaction^d^** |
| --- | --- | --- | --- | --- | --- | --- | --- | --- |
| **Poorly conserved** |  |  |  |  |  |  |  |  |
| I set domains | b.1.1.4 | d1jbja1 | 77 | 217 | L/T | 26 | 54 | H-P |
|  |  | d1nkr_1 |  |  | I/N | 20 | 58 | H-P |
|  |  | d2fcba2 |  |  | V/N | 19 | 50 | H-P |
|  |  | d1cs6a1 |  |  | T/D | 26 | 62 | Hy |
|  |  | d1f2qa1 |  |  | T/S | 20 | 45 | Hy |
|  |  | d1fhga_ |  |  | R/S | 27 | 66 | Hy |
|  |  | d1g0xa1 |  |  | T/Q | 22 | 55 | Hy |
|  |  | d1gxea_ |  |  | D/S | 30 | 94 | Hy |
|  |  | d1iile1 |  |  | K/S | 27 | 66 | Hy |
|  |  | d1n26a1 |  |  | T/R | 25 | 61 | Hy |
|  |  | d1ncu__ |  |  | R/T | 25 | 63 | Hy |
|  |  | d1nkr_2 |  |  | T/D | 24 | 62 | Hy |
|  |  | d1olla1 |  |  | T/K | 22 | 58 | Hy |
|  |  | d1olza1 |  |  | E/N | 16 | 51 | Hy |
|  |  | d1qz1a3 |  |  | T/E | 24 | 62 | Hy |
|  |  | d1vcaa2 |  |  | S/T | 20 | 55 | Hy |
|  |  | d1wiu__ |  |  | N/S | 21 | 59 | Hy |
|  |  | d2fcba1 |  |  | T/S | 23 | 53 | Hy |
|  |  | d1cs6a3 |  |  | T/L | 24 | 56 | P-H |
|  |  | d1gl4b_ |  |  | T/I | 21 | 55 | P-H |
|  |  | d1gsma1 |  |  | Q/V | 22 | 56 | P-H |
|  |  | d1iile2 |  |  | E/V | 25 | 64 | P-H |
|  |  | d1rhfa2 |  |  | Q/V | 20 | 52 | P-H |
|  |  | d1tit__ |  |  | H/I | 20 | 57 | P-H |
|  |  | d1cs6a2 |  |  | M/N | 23 | 62 | P |
|  |  | d1cs6a4 |  |  | R/E | 20 | 54 | Sa |
|  |  | d1g1ca_ |  |  | H/E | 23 | 62 | Sa |
|  |  | d1jbja2 |  |  | E/H | 18 | 45 | Sa |
|  |  | d1ucta1 |  |  | K/E | 25 | 59 | Sa |
|  |  | d1zxq_2 |  |  | E/H | 21 | 53 | Sa |
| I set domains | b.1.1.4 | d1iile1 | 101 | 216 | M/W | 40 | 65 | A-Ar |
|  |  | d1vcaa2 |  |  | F/S | 33 | 54 | A-Ar |
|  |  | d1cs6a3 |  |  | I/P | 37 | 55 | H |
|  |  | d1cs6a4 |  |  | V/G | 33 | 53 | H |
|  |  | d1gl4b_ |  |  | L/G | 35 | 54 | H |
|  |  | d1gsma2 |  |  | L/V | 35 | 73 | H |
|  |  | d1he7a_ |  |  | L/G | 32 | 63 | H |
|  |  | d1nkr_1 |  |  | F/A | 32 | 57 | H |
|  |  | d1wwca_ |  |  | L/G | 34 | 62 | H |
|  |  | d2fcba1 |  |  | I/P | 37 | 52 | H |
|  |  | d2fcba2 |  |  | V/P | 33 | 49 | H |
|  |  | d1epfa1 |  |  | I/S | 33 | 60 | H-P |
|  |  | d1epfa2 |  |  | I/N | 33 | 56 | H-P |
|  |  | d1f2qa2 |  |  | V/H | 33 | 49 | H-P |
|  |  | d1f42a1 |  |  | I/K | 36 | 51 | H-P |
|  |  | d1fltx_ |  |  | V/K | 36 | 59 | H-P |
|  |  | d1gsma1 |  |  | V/S | 36 | 55 | H-P |
|  |  | d1gxea_ |  |  | V/R | 41 | 93 | H-P |
|  |  | d1iam_2 |  |  | L/K | 31 | 50 | H-P |
|  |  | d1iile2 |  |  | I/E | 38 | 63 | H-P |
|  |  | d1jbja1 |  |  | I/N | 36 | 53 | H-P |
|  |  | d1jbja2 |  |  | L/K | 29 | 44 | H-P |
|  |  | d1n26a1 |  |  | V/R | 39 | 60 | H-P |
|  |  | d1ncu__ |  |  | V/S | 38 | 62 | H-P |
|  |  | d1olza1 |  |  | V/K | 28 | 50 | H-P |
|  |  | d1rhfa2 |  |  | I/S | 34 | 51 | H-P |
|  |  | d1tit__ |  |  | G/H | 32 | 56 | H-P |
|  |  | d1wiu__ |  |  | A/T | 34 | 58 | H-P |
|  |  | d1zxq_2 |  |  | G/K | 34 | 52 | H-P |
|  |  | d1cs6a1 |  |  | Y/G | 39 | 61 | H/SP-H |

Table.S1 (continued)

| **SCOP family name** | **SCOP code** | **SCOP domain** | **Distinct disulphide bond^a^** | | **Substituted residues^b^** | **Original residue positions^c^** | | **Possible interaction^d^** |
| --- | --- | --- | --- | --- | --- | --- | --- | --- |
| **Poorly conserved** |  |  |  |  |  |  |  |  |
| I set domains (continued) | b.1.1.4 | d1cs6a2 | 101 | 216 | Y/G | 37 | 61 | H/SP-H |
|  |  | d1f97a2 |  |  | Y/G | 35 | 62 | H/SP-H |
|  |  | d1g0xa1 |  |  | Y/G | 31 | 54 | H/SP-H |
|  |  | d1nkr_2 |  |  | Y/A | 36 | 61 | H/SP-H |
|  |  | d1olla1 |  |  | Y/V | 34 | 57 | H/SP-H |
|  |  | d1f2qa1 |  |  | T/S | 30 | 44 | Hy |
|  |  | d1ucta1 |  |  | T/P | 37 | 58 | P-H |
|  |  | d1qz1a3 |  |  | M/S | 37 | 61 | P |
| TNF-like | b.22.1.1 | d1c3ha_ | 107 | 132 | K/Y | 65 | 79 | A-Ar |
|  |  | d1o91a_ |  |  | W/Y | 62 | 76 | Ar |
|  |  | d1aly__ |  |  | I/A | 75 | 94 | H |
|  |  | d1dg6a_ |  |  | V/A | 75 | 94 | H |
|  |  | d1iqaa_ |  |  | L/G | 76 | 95 | H |
|  |  | d1kxga_ |  |  | G/I | 68 | 92 | H |
|  |  | d1rj8a_ |  |  | S/T | 70 | 84 | Hy |
|  |  | d2tnfa_ |  |  | T/V | 68 | 88 | P-H |
|  |  | d1aly__ | 131 | 156 | A/I | 93 | 108 | H |
|  |  | d1iqaa_ |  |  | G/I | 94 | 111 | H |
|  |  | d1pk6a_ |  |  | F/G | 78 | 92 | H |
|  |  | d1pk6c_ |  |  | F/G | 76 | 89 | H |
|  |  | d2tnfa_ |  |  | A/I | 87 | 109 | H |
|  |  | d1c3ha_ |  |  | T/G | 78 | 92 | P-H |
|  |  | d1dg6a_ |  |  | S/I | 93 | 110 | P-H |
|  |  | d1o91a_ |  |  | T/G | 75 | 89 | P-H |
| Glycosyl hydrolase family 7 catalytic core | b.29.1.10 | d1ojja_ | 3 | 74 | G/I | 3 | 73 | H |
| Xylanase/endoglucanase 11/12 | b.29.1.11 | d1h0ba_ | 165 | 219 | L/A | 146 | 181 | H |
|  |  | d1h4ga_ |  |  | V/N | 116 | 160 | H-P |
|  |  | d1olra_ |  |  | L/D | 142 | 178 | H-P |
|  |  | d2nlra_ |  |  | V/D | 143 | 178 | H-P |
|  |  | d1bk1__ |  |  | S/N | 102 | 146 | Hy |
| Fibroblast growth factors (FGF) | b.42.1.1 | d1bfg__ | 48 | 60 | F/G | 12 | 24 | H |
|  |  | d1ihka_ |  |  | F/G | 21 | 33 | H |
|  |  | d1q1ua_ |  |  | Y/G | 16 | 28 | H/SP-H |
|  |  | d1m2tb1 | 137 | 158 | V/L | 106 | 123 | H |
|  |  | d1m2tb2 |  |  | A/A | 96 | 114 | H |
|  |  | d1qxma2 |  |  | V/Y | 106 | 125 | H-H/SP |
|  |  | d1qxma1 |  |  | V/Q | 112 | 126 | H-P |
| Prokaryotic proteases | b.47.1.1 | d1arb__ | 231 | 255 | I/N | 145 | 159 | H-P |
|  |  | d1lcya2 |  |  | V/S | 120 | 134 | H-P |
|  |  | d1p3ca_ |  |  | K/S | 128 | 149 | Hy |
|  |  | d1qy6a_ |  |  | T/K | 130 | 147 | Hy |
|  |  | d1sgpe_ |  |  | T/S | 98 | 108 | Hy |
|  |  | d1agja_ |  |  | E/E | 157 | 174 | P |
| Pleckstrin-homology domain (PH domain) | b.55.1.1 | d1btka_ | 214 | 255 | P/F | 89 | 104 | H |
|  |  | d1dyna_ |  |  | I/A | 68 | 89 | H |
|  |  | d1btn__ |  |  | V/Q | 75 | 89 | H-P |
|  |  | d1dbha2 |  |  | A/S | 92 | 106 | H-P |
|  |  | d1dro__ |  |  | V/Q | 86 | 100 | H-P |
|  |  | d1eaza_ |  |  | L/Q | 68 | 81 | H-P |
|  |  | d1pls__ |  |  | V/Q | 68 | 82 | H-P |
|  |  | d1fhoa_ |  |  | T/K | 73 | 89 | Hy |
|  |  | d1h10a_ |  |  | T/H | 71 | 88 | Hy |
|  |  | d1kz7a2 |  |  | K/Q | 86 | 101 | Hy |
|  |  | d1ki1b2 |  |  | D/R | 90 | 103 | Sa |
| Fatty acid binding protein-like | b.60.1.2 | d1lfo__ | 149 | 158 | M/Y | 112 | 119 | A-Ar |
|  |  | d1o1va_ |  |  | S/Y | 112 | 119 | A-Ar |
|  |  | d1mdc__ |  |  | I/A | 116 | 125 | H |
|  |  | d1p6pa_ |  |  | I/L | 111 | 118 | H |

Table.S1 (continued)

| **SCOP family name** | **SCOP code** | **SCOP domain** | **Distinct disulphide bond^a^** | | **Substituted residues^b^** | **Original residue positions^c^** | | **Possible interaction^d^** |
| --- | --- | --- | --- | --- | --- | --- | --- | --- |
| **Poorly conserved** |  |  |  |  |  |  |  |  |
| Fatty acid binding protein-like | b.60.1.2 | d1ifc__ | 149 | 158 | Y/A | 117 | 124 | H/SP-H |
| Hemopexin-like domain | b.66.1.1 | d1itva_ | 176 | 201 | T/F | 149 | 161 | A-Ar |
|  |  | d1fbl_1 |  |  | V/F | 155 | 167 | H |
|  |  | d1gen__ |  |  | L/F | 157 | 171 | H |
|  |  | d1hxn__ |  |  | V/L | 151 | 175 | H |
| WD40-repeat | b.69.4.1 | d1erja_ | 260 | 314 | V/V | 162 | 193 | H |
|  |  | d1gxra_ |  |  | F/I | 155 | 187 | H |
|  |  | d1k8kc_ |  |  | A/V | 157 | 207 | H |
|  |  | d1nr0a1 |  |  | A/V | 118 | 152 | H |
|  |  | d1nr0a2 |  |  | V/V | 115 | 142 | H |
|  |  | d1pgua2 |  |  | A/V | 93 | 124 | H |
|  |  | d1tbga_ |  |  | V/L | 158 | 190 | H |
| delta-Endotoxin (insectocide), middle domain | b.77.2.1 | d1ciy_2 | 118 | 181 | V/I | 95 | 146 | H |
|  |  | d1ji6a2 |  |  | L/T | 103 | 150 | H-P |
| RuBisCo, large subunit, C-terminal domain | c.1.14.1 | d5ruba1 | 28 | 48 | T/F | 26 | 46 | A-Ar |
| Type II chitinase | c.1.8.5 | d1d2ka1 | 32 | 87 | V/F | 7 | 29 | H |
|  |  | d1edt__ |  |  | A/F | 8 | 33 | H |
|  |  | d1eoka_ |  |  | A/V | 6 | 26 | H |
|  |  | d1goia2 |  |  | G/L | 7 | 42 | H |
|  |  | d1itxa1 |  |  | G/V | 16 | 38 | H |
|  |  | d1kfwa1 |  |  | G/L | 13 | 39 | H |
|  |  | d1cnv__ |  |  | V/Y | 9 | 28 | H-H/SP |
|  |  | d1om0a_ |  |  | V/Y | 10 | 30 | H-H/SP |
|  |  | d1edqa2 |  |  | S/L | 30 | 52 | P-H |
|  |  | d2ebn__ |  |  | S/V | 10 | 35 | P-H |
|  |  | d1nar__ |  |  | E/E | 7 | 32 | P |
| Subtilases | c.41.1.1 | d1gci__ | 90 | 105 | G/T | 46 | 56 | H-P |
|  |  | d1ic6a_ |  |  | V/S | 56 | 63 | H-P |
|  |  | d1r64a2 |  |  | S/P | 74 | 84 | P-H |
|  |  | d1r64a2 | 258 | 353 | I/Q | 214 | 291 | H-P |
|  |  | d1gci__ |  |  | M/R | 169 | 241 | P |
| Higher-molecular-weight phosphotyrosine protein phosphatases | c.45.1.2 | d1g4us2 | 138 | 179 | P/L | 54 | 90 | H |
|  |  | d1lyva_ |  |  | P/M | 77 | 113 | H-P |
| Pancreatic carboxypeptidases | c.56.5.1 | d1m4la_ | 71 | 84 | L/G | 66 | 79 | H |
|  |  | d1jqga1 |  |  | S/T | 69 | 82 | Hy |
|  |  | d1jqga1 | 161 | 175 | G/H | 153 | 166 | H-P |
|  |  | d1m4la_ |  |  | G/H | 152 | 166 | H-P |
|  |  | d1jqga1 | 220 | 257 | G/F | 210 | 247 | H |
|  |  | d1m4la_ |  |  | T/I | 210 | 244 | P-H |
| Protein-L-isoaspartyl O-methyltransferase | c.66.1.7 | d1i1na_ | 132 | 156 | V/V | 114 | 138 | H |
|  |  | d1jg1a_ |  |  | V/L | 114 | 133 | H |
| Fungal ribonucleases | d.1.1.4 | d1i0va_ | 28 | 97 | Y/N | 24 | 84 | A-Ar |
|  |  | d1rtu__ |  |  | L/G | 27 | 92 | H |
| Cytochrome b5 | d.120.1.1 | d1mj4a_ | 28 | 57 | I/A | 19 | 48 | H |
|  |  | d1cyo__ |  |  | T/A | 21 | 50 | P-H |
| Fluorescent proteins | d.22.1.1 | d1ggxa_ | 148 | 203 | T/D | 138 | 187 | Hy |
|  |  | d1oxda_ |  |  | N/S | 146 | 199 | Hy |
| Pilin | d.24.1.1 | d1dzoa_ | 59 | 106 | V/I | 33 | 74 | H |

^a^ Start and end positions of the disulphide bonded cysteines in the structural alignment of the members of the family.

^b^ Substituted residues in the homologous members with no disulphide bond. The single letter code of amino acids is used.

^c^ Residue number of the substituted residues from their original PDB files

^d^ Possible interaction of the substituted residues. A – Aliphatic; Ar – Aromatic; H – Hydrophobic; Hy – Hydrogen bonding; P – Polar; SP – Slightly polar
