# Supplementary material for: Analysis on conservation of disulphide bonds and their structural features in homologous protein domain families
Source: BMC Struct Biol. 2008 Dec 26;8:55. doi: 10.1186/1472-6807-8-55 (PMC2628669; doi:10.1186/1472-6807-8-55)
Supplement: Additional file 2 — Evolutionary dynamics of disulphides at family and superfamily level. We extended our analysis by investigating the evolutionary dynamics of disulphides between members within a homologous family and also across families in a superfamily in order to provide further insights into the biological implications of conservation and non-conservation of disulphides. [file 1472-6807-8-55-S2.doc]

#### Evolutionary dynamics of disulphides at family and superfamily level

As stated in the previous sections, non-conservation of distinct disulphide bonds within homologous families is a very common feature and we analysed its biological implications through several examples. Here, we extend our analysis by investigating the evolutionary dynamics of disulphides between members within a homologous family and across families in a superfamily in order to provide further insights into the biological implications of conservation and non-conservation of disulphides. To illustrate this, we selected the large superfamily of alpha/beta hydrolases (SCOP superfamily code: C.69.1.). We primarily focused on the acetylcholinesterase-like family (SCOP supefamily code: c.69.1.1) whereby the members share pairwise sequence identities between 31% and 83%. This family harbours five types of esterase domains: cholesterol esterases (SCOP domain: d1f6wa and d2bce__), actetyl and butyryl cholineesterase (SCOP domain: d1ea5a_ and d1p0ia_), mammalian carboxyesterase (SCOP domain: d1mx1a_ and d1k4ya_) and thermophilic para-nitrobenzyl (PNB) esterase (SCOP domain: d1qe3a_). A total of three distinct disulphides are featured in this family. Out of these, two are highly conserved across the members of the family. Interestingly, the third disulphide is specific to the acetyl or butyryl cholinesterase type. On the other hand, the PNB esterases do not host any of these three disulphides though two free cysteines, which are topologically equivalent to one disulphide, are featured. Hence three groups of esterases can be distinguished within the acetylcholinesterase-like family based on the disulphide profiles; the ones with two disulphide bonds which includes the cholesterol esterase and carboxyesterase type I; the ones with three disulphides which includes the acetyl and butyryl cholinesterases; the ones with no disulphide bonds featured is the one containing PNB esterases. Representatives from these three groups are featured in Figure.S1 where the disulphides are highlighted. The additional disulphide in the acetyl or butyryl cholinesterase group is indeed bridging two α-helices, but this disulphide is missing in the cholesterol esterase type and is compensated by favourable aliphatic-aliphatic interactions between substituted residues (Ile395 and Leu519).

Similarly, when homologous members from the serine carboxypeptidase-like family (SCOP family code: c.69.1.5) are analysed, a single strictly conserved disulphide is found between the serine carboxypeptidase II and the human carboxypeptidase L or human 'protective protein' (HPP) subfamilies. This disulphide is indeed featured in a highly superimposable portion of the proteins (Figure.S2). However, the other disulphides are not conserved because they are located in regions that are non-superimposable and hence not topologically equivalent. It is apparent from this comparison that non-conservation of disulphides within this serine carboxypeptidase-like family is associated with recruitment of new structural features that are specific to the subfamilies.

Surprisingly, when a member (SCOP domain: d1thg__) of the fungal lipase family (SCOP family code: c.69.1.17) (Figure.S3) is compared to the members of acetylcholinesterase-like family (SCOP family code: c.69.1.1), both highly conserved disulphides from the latter are superimposable with the two disulphides from ‘d1thg__’. In contrast, although a superimposable core region could be identified between members from acetylcholinesterase-like (SCOP family code: c.69.1.1) and serine carboxypeptidase-like (SCOP family code: c.69.1.5) families, they do not share topologically equivalent disulphides. Their respective disulphides are not superimposable. This hence illustrates non-conserved disulphide bonds across superfamily members have limited effect on their common fold, but would be associated with recruitment of new structural features.

**Figure.S1.** Evolutionary dynamics of disulphides in the alpha/beta hydrolase actetylcholinesterase-like family (SCOP family code: c.69.1.1).

Three highly superimposable members from this family are featured. In (a) is featured a representative (SCOP domain: d2bce__; PDB code: 2bce) from the cholesterol esterase and carboxyesterase types which display two highly conserved disulphides. In (b) is featured a representative (SCOP domain: d1dx4a_; PDB code: 1dx4) from the acetylcholinesterase type which display in addition to the two conserved disulphide, a third specific disulphide (arrow). In (c) is featured a representative from the paranitrobenzyl esterases group which do not feature any disulphide though two cysteines which are topologically equivalent to one highly conserved disulphide is present in the molecule. Cysteines involved in disulphide bonds are shown in yellow (spacefill). Figure is drawn using Chimera (Ref).

**Figure.S2.** Evolutionary dynamics of disulphides in the alpha/beta hydrolase serine carboxypeptidase-like family (SCOP code: c.69.1.5).

Three superimposable members from this family are featured and which display one highly conserved disulphide (arrow). In (a) is featured a representative (SCOP domain : d1ac5_; PDB: 1ac5) from the serine carboxypeptidase II type and which displays two non-conserved disulphides. In (b) is featured a representative (SCOP domain: d1cpya_; PDB: 1cpy) from the human carboxypeptidase L type which display in addition to the conserved disulphide, four specific disulphides. In (c) is featured a representative from the human protective protein group which display in addition to the conserved disulphide, three specific disulphides. Cysteines are involved in disulphide bonds are shown in yellow (spacefill). Figure is drawn using Chimera (Ref).

**Figure.S3.** Representation of Type-B carboxylesterase/lipase (SCOP domain: d1thga_; PDB: 1thg) from the fungal lipase family (SCOP family code: c.69.1.17) that features two disulphides that are topologically equivalent to the two highly conserved disulphides from acetylcholinesterase-like family (SCOP family code: c.69.1.1). Cysteines are involved in disulphide bonds are shown in yellow (spacefill). Figure is drawn using Chimera (Ref).

Figure S1-S3 are drawn by using Chimera:

Pettersen, E.F., Goddard, T.D., Huang, C.C., Couch, G.S., Greenblatt, D.M., Meng, E.C., and Ferrin, T.E. "UCSF Chimera - A Visualization System for Exploratory Research and Analysis." *J. Comput. Chem.* **25**(13):1605-1612 (2004).
