# Supplementary figures and images for: Analysis on conservation of disulphide bonds and their structural features in homologous protein domain families
Source: BMC Struct Biol. 2008 Dec 26;8:55. doi: 10.1186/1472-6807-8-55 (PMC2628669; doi:10.1186/1472-6807-8-55)

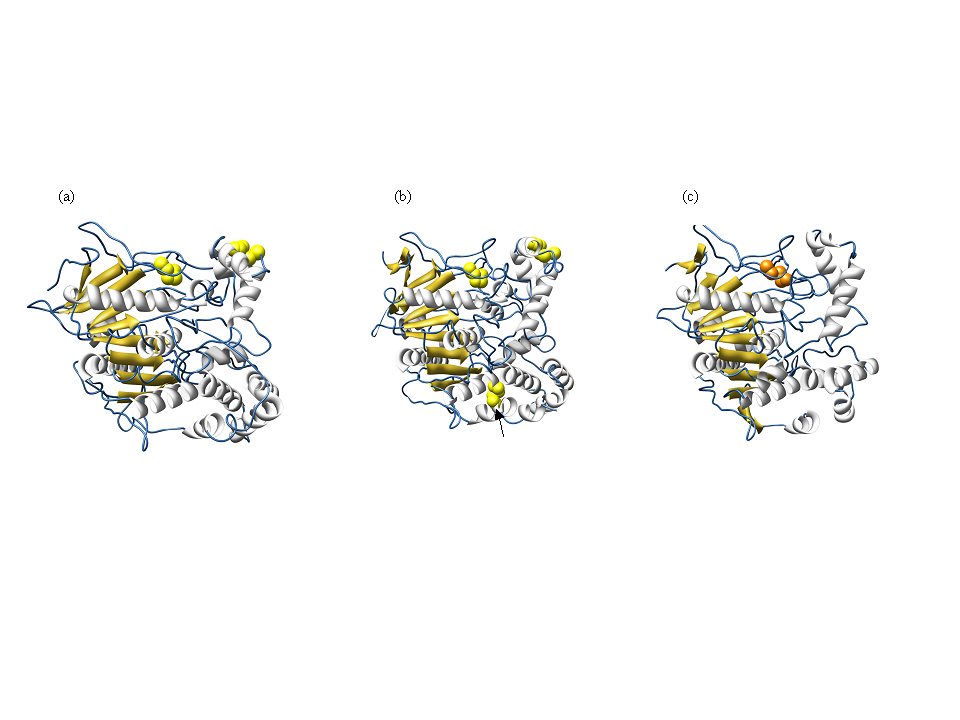

Supplement: Additional file 3 — Evolutionary dynamics of disulphides in the alpha/beta hydrolase actetylcholinesterase-like family (SCOP family code: c.69.1.1). Three highly superimposable members from this family are featured. In (a) is featured a representative (SCOP domain: d2bce__; PDB code: 2bce) from the cholesterol esterase and carboxyesterase types which display two highly conserved disulphides. In (b) is featured a representative (SCOP domain: d1dx4a_; PDB code: 1dx4) from the acetylcholinesterase type which display in addition to the two conserved disulphide, a third specific disulphide (arrow). In (c) is featured a representative from the paranitrobenzyl esterases group which do not feature any disulphide though two cysteines which are topologically equivalent to one highly conserved disulphide is present in the molecule. [file 1472-6807-8-55-S3.tiff]

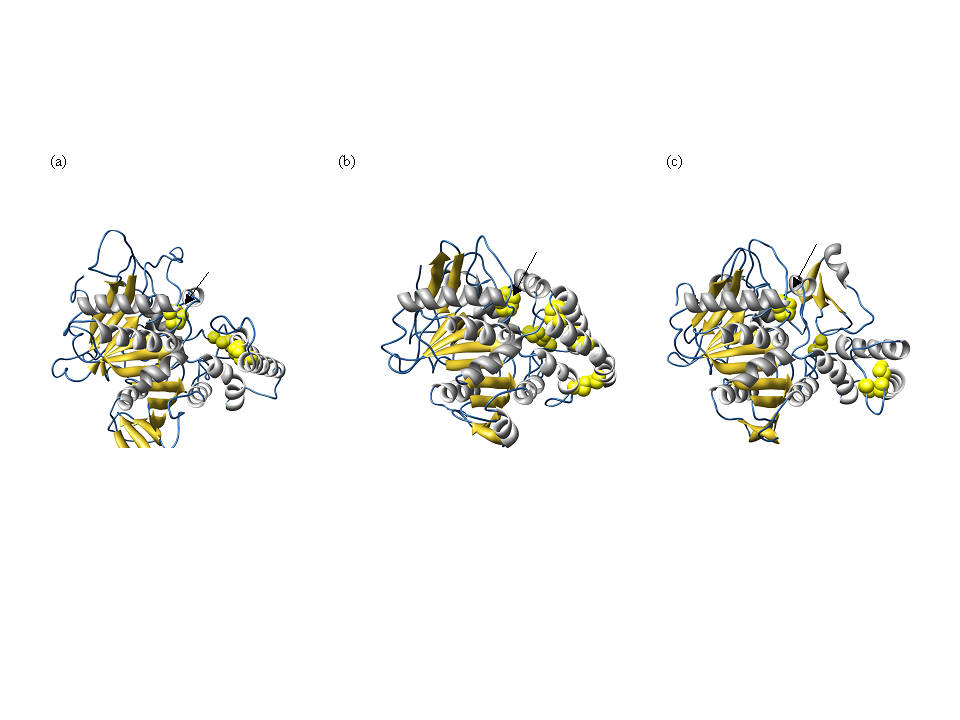

Supplement: Additional file 4 — Evolutionary dynamics of disulphides in the alpha/beta hydrolase serine carboxypeptidase-like family (SCOP code: c.69.1.5). Three superimposable members from this family are featured and which display one highly conserved disulphide (arrow). In (a) is featured a representative (SCOP domain: d1ac5_; PDB: 1ac5) from the serine carboxypeptidase II type and which displays two non-conserved disulphides. In (b) is featured a representative (SCOP domain: d1cpya_; PDB: 1cpy) from the human carboxypeptidase L type which display in addition to the conserved disulphide, four specific disulphides. In (c) is featured a representative from the human protective protein group which display in addition to the conserved disulphide, three specific disulphides. [file 1472-6807-8-55-S4.tiff]

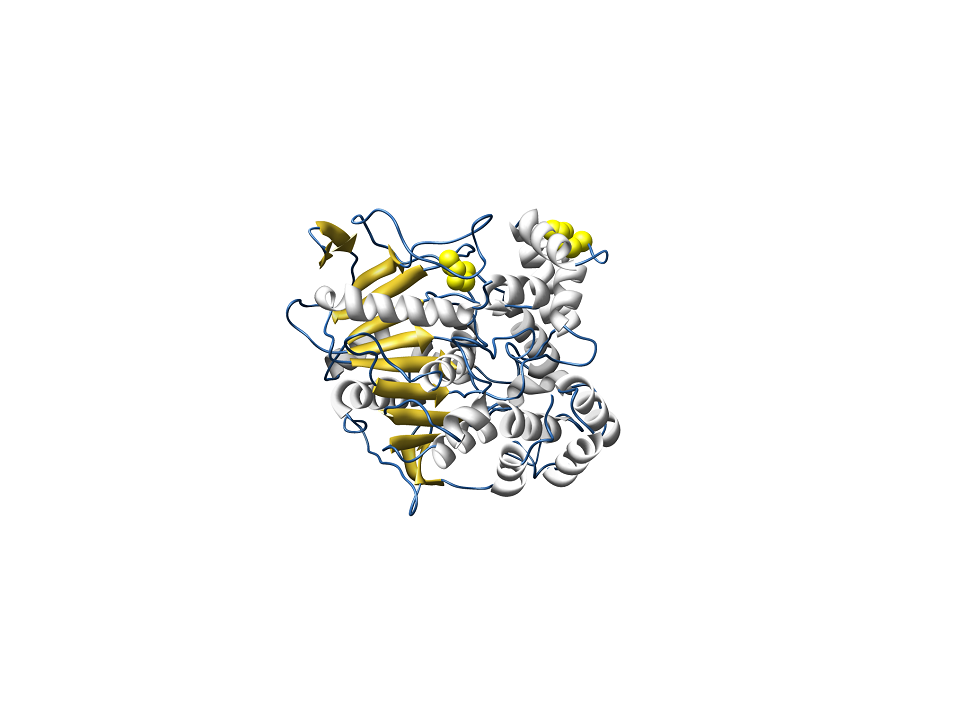

Supplement: Additional file 5 — Representation of Type-B carboxylesterase/lipase (SCOP domain: d1thga_; PDB: 1thg) from the fungal lipase family (SCOP family code: c.69.1.17). Representation of Type-B carboxylesterase/lipase (SCOP domain: d1thga_; PDB: 1thg) from the fungal lipase family (SCOP family code: c.69.1.17) that features two disulphides that are topologically equivalent to the two highly conserved disulphides from acetylcholinesterase-like family (SCOP family code: c.69.1.1). [file 1472-6807-8-55-S5.tiff]
