# Supplementary material for: Analysis on conservation of disulphide bonds and their structural features in homologous protein domain families
Source: BMC Struct Biol. 2008 Dec 26;8:55. doi: 10.1186/1472-6807-8-55 (PMC2628669; doi:10.1186/1472-6807-8-55)
Supplement: Additional file 6 — Localization predictions by the three programs and the consensus starting from full-length sequences of proteins in the dataset. This table provides a comparison and consensus obtained by applying the three prediction servers for cellular localization. [file 1472-6807-8-55-S6.pdf]

Supplementary Table 1 : Localization predictions by the three programs and the consensus starting from full-length sequences of proteins in the dataset

	SUBLOC	PSORT	TARGETP	Consensus
1A06A	Intracellular	Intracellular	_	
1A0JA	Intracellular	Extracellular	_	
1A15A	Intracellular	Extracellular	Extracellular	Extracellular
1A15B	Intracellular	Extracellular	Extracellular	Extracellular
1A1NA	Extracellular	Extracellular	Extracellular	Extracellular
1A21A	Extracellular	Extracellular	Extracellular	Extracellular
1A3PA	Extracellular	Extracellular	Extracellular	Extracellular
1A3RH	Intracellular	Intracellular	_	
1A4KH	Intracellular	Extracellular	Intracellular	Intracellular
1A4PA	Intracellular	Intracellular	_	
1A57A	Intracellular	Intracellular	_	
1A75A	Extracellular	Intracellular	_	
1A7SA	Intracellular	Extracellular	Extracellular	Extracellular
1AAPA	Intracellular	Extracellular	Extracellular	Extracellular
1ACWA	Extracellular		_	
1ADXA	Extracellular	Extracellular	Extracellular	Extracellular
1ADZA	Extracellular	Extracellular	Extracellular	Extracellular
1AG7A	Extracellular	Extracellular	_	
1AGDA	Extracellular	Extracellular	Extracellular	Extracellular
1AGGA	Intracellular	Extracellular	Extracellular	Extracellular
1AGIA	Intracellular	Extracellular	Extracellular	Extracellular
1AGQA	Intracellular	Extracellular	Extracellular	Extracellular
1AGTA	Extracellular	Extracellular	_	
1AHLA	Extracellular	Extracellular	_	
1AHMA	Intracellular	Extracellular	Extracellular	Extracellular
1AHOA	Extracellular	Extracellular	Extracellular	Extracellular
1AINA	Intracellular	Intracellular	_	
1AIUA	Extracellular	Extracellular	_	
1AJJA	Extracellular	Intracellular	Extracellular	Extracellular
1AJSA	Intracellular	Intracellular	_	
1AK2A	Intracellular	Intracellular	_	

1ALAA	Intracellular	Intracellular	_	
1ALCA	Intracellular	Extracellular	_	
1ALUA	Extracellular	Extracellular	Extracellular	Extracellular
1ALYA	Intracellular	Extracellular	Extracellular	Extracellular
1AM5A	Extracellular	Intracellular	_	
1AN1I	Extracellular	Extracellular	_	
1AOGA	Intracellular	Intracellular	Extracellular	Intracellular
1AOXA	Extracellular	Extracellular	Extracellular	Extracellular
1AP4A	Intracellular	Intracellular	_	
1APFA	Extracellular	Extracellular	_	
1APJA	Extracellular	Intracellular	Extracellular	Extracellular
1APOA	Extracellular	Extracellular	Extracellular	Extracellular
1APQA	Extracellular	Extracellular	Extracellular	Extracellular
1AQKL	Extracellular	Extracellular	_	
1AQUA	Intracellular	Intracellular	_	
1AQWA	Intracellular	Intracellular	Intracellular	Intracellular
1ARKA	Intracellular	Intracellular	_	
1ATAA	Extracellular	Extracellular	_	
1ATLA	Intracellular	Extracellular	Extracellular	Extracellular
1ATXA	Extracellular	Extracellular	_	
1ATZA	Extracellular	Extracellular	Extracellular	Extracellular
1AU1A	Intracellular	Extracellular	Extracellular	Extracellular
1AUIB	Intracellular	Intracellular	_	
1AUKA	Extracellular	Extracellular	Extracellular	Extracellular
1AV3A	Intracellular	Extracellular	Extracellular	Extracellular
1AVSA	Intracellular	Intracellular	_	
1AWJA	Intracellular	Intracellular	_	
1AWWA	Intracellular	Intracellular	_	
1AX8A	Intracellular	Extracellular	Extracellular	Extracellular
1AXHA	Extracellular	Extracellular	_	
1AXIA	Intracellular	Extracellular	Extracellular	Extracellular
1AXIB	Intracellular	Intracellular	Extracellular	Intracellular
1AXNA	Intracellular	Intracellular	_	
1AYAA	Intracellular	Intracellular	_	
1AYEA	Intracellular	Extracellular	Extracellular	Extracellular

1AYOA	Extracellular	Intracellular	_	
1AZSC	Intracellular	Intracellular	_	
1AZZA	Intracellular	Extracellular	_	
1B09A	Extracellular	Extracellular	Extracellular	Extracellular
1B16A	Intracellular	Intracellular	_	
1B2TA	Intracellular	Intracellular	Extracellular	Intracellular
1B4QA	Intracellular	Intracellular	_	
1B50A	Extracellular	Extracellular	Extracellular	Extracellular
1B56A	Intracellular	Extracellular	_	
1B5AA	Intracellular	Extracellular	_	
1B5LA	Intracellular	Extracellular	Extracellular	Extracellular
1B6VA	Intracellular	Extracellular	Extracellular	Extracellular
1B8WA	Intracellular	Extracellular	Extracellular	Extracellular
1B9GA	Intracellular	Extracellular	Extracellular	Extracellular
1B9OA	Intracellular	Extracellular	Extracellular	Extracellular
1BAHA	Extracellular	Extracellular	Extracellular	Extracellular
1BAKA	Intracellular	Intracellular	_	
1BB9A	Intracellular	Intracellular	_	
1BBPA	Extracellular	Extracellular	Extracellular	Extracellular
1BBZA	Intracellular	Intracellular	_	
1BCGA	Extracellular	Extracellular	Extracellular	Extracellular
1BDSA	Extracellular	Extracellular	_	
1BEBA	Extracellular	Extracellular	Extracellular	Extracellular
1BEIA	Extracellular	Extracellular	_	
1BETA	Extracellular	Extracellular	Extracellular	Extracellular
1BF0A	Extracellular	Extracellular	_	
1BFGA	Intracellular	Intracellular	_	
1BG1A	Intracellular	Intracellular	_	
1BGCA	Extracellular	Extracellular	Extracellular	Extracellular
1BGEA	Extracellular	Extracellular	_	
1BGKA	Extracellular	Extracellular	_	
1BHTA	Extracellular	Extracellular	Extracellular	Extracellular
1BIGA	Extracellular	Extracellular	Extracellular	Extracellular
1BIKA	Extracellular	Extracellular	Extracellular	Extracellular
1BIOA	Intracellular	Extracellular	Extracellular	Extracellular

1BJ7A	Intracellular	Extracellular	Extracellular	Extracellular
1BJFA	Extracellular	Intracellular	_	
1BKTA	Intracellular	Extracellular	Extracellular	Extracellular
1BLAA	Intracellular	Intracellular	_	
1BLJA	Intracellular	Intracellular	_	
1BLXA	Intracellular	Intracellular	_	
1BMGA	Intracellular	Extracellular	Extracellular	Extracellular
1BMRA	Extracellular	Extracellular	_	
1BNBA	Extracellular	Extracellular	_	
1BNDA	Intracellular	Extracellular	Extracellular	Extracellular
1BNDB	Intracellular	Extracellular	Extracellular	Extracellular
1BO0A	Extracellular	Extracellular	Extracellular	Extracellular
1BO9A	Intracellular	Intracellular	_	
1BOFA	Intracellular	Intracellular	_	
1BPVA	Intracellular		Extracellular	
1BQUA	Extracellular	Intracellular	Extracellular	Extracellular
1BQYA	Intracellular	Extracellular	Extracellular	Extracellular
1BR9A	Extracellular	Extracellular	Extracellular	Extracellular
1BRBI	Extracellular	Extracellular	Extracellular	Extracellular
1BRUP	Extracellular	Extracellular	Extracellular	Extracellular
1BTEA	Intracellular	Extracellular	Extracellular	Extracellular
1BTKA	Intracellular	Intracellular	_	
1BTNA	Intracellular	Intracellular	_	
1BU3A	Extracellular	Intracellular	_	
1BU8A	Extracellular	Extracellular	Extracellular	Extracellular
1BUDA	Intracellular	Extracellular	Extracellular	Extracellular
1BUNA	Extracellular	Extracellular	Extracellular	Extracellular
1BUNB	Extracellular	Extracellular	Extracellular	Extracellular
1BUSA	Intracellular	Extracellular	Extracellular	Extracellular
1BV8A	Extracellular	Extracellular	Extracellular	Extracellular
1BW0A	Intracellular	Intracellular	_	
1BWYA	Intracellular	Intracellular	_	
1BX4A	Intracellular	Intracellular	_	
1BX7A	Extracellular	Extracellular	_	
1BYFA	Intracellular	Intracellular	_	

1BYGA	Intracellular	Intracellular	_	
1BYUA	Intracellular	Intracellular	_	
1C1EH	Extracellular	Intracellular	_	
1C1YA	Intracellular	Intracellular	_	
1C2UA	Extracellular	Extracellular	_	
1C3HA	Extracellular	Extracellular	Extracellular	Extracellular
1C49A	Extracellular	Extracellular	_	
1C55A	Extracellular	Extracellular	_	
1C5AA	Intracellular	Extracellular	_	
1C6WA	Extracellular	Extracellular	_	
1C7VA	Intracellular	Intracellular	_	
1CB6A	Extracellular	Extracellular	Extracellular	Extracellular
1CB9A	Extracellular	Extracellular	_	
1CBSA	Intracellular	Intracellular	_	
1CCVA	Extracellular	Extracellular	_	
1CCZA	Extracellular	Extracellular	Extracellular	Extracellular
1CDOA	Intracellular	Intracellular	_	
1CDPA	Extracellular	Intracellular	_	
1CDQA	Extracellular	Extracellular	Extracellular	Extracellular
1CDTA	Extracellular	Extracellular	_	
1CDWA	Intracellular	Intracellular	_	
1CDYA	Extracellular	Intracellular	Extracellular	Extracellular
1CE2A	Extracellular	Extracellular	Extracellular	Extracellular
1CEAA	Extracellular	Extracellular	Extracellular	Extracellular
1CEJA	Intracellular	Intracellular	Extracellular	Intracellular
1CEWI	Intracellular	Extracellular	Extracellular	Extracellular
1CFBA	Intracellular	Intracellular	Extracellular	Intracellular
1CGHA	Intracellular	Extracellular	Extracellular	Extracellular
1CHLA	Extracellular	Extracellular	_	
1CHVS	Extracellular	Extracellular	_	
1CIPA	Intracellular	Intracellular	_	
1CIXA	Extracellular	Extracellular	Extracellular	Extracellular
1CJMA	Intracellular	Intracellular	_	
1CK4A	Intracellular	Extracellular	Extracellular	Extracellular
1CKAA	Intracellular	Intracellular	_	

1CKIA	Intracellular	Intracellular	_	
1CM8A	Intracellular	Intracellular	_	
1CMGA	Intracellular	Intracellular	_	
1CMRA	Extracellular	Extracellular	Extracellular	Extracellular
1CN2A	Extracellular	Extracellular	Extracellular	Extracellular
1CNNA	Intracellular		_	
1CNT1	Intracellular	Intracellular	Intracellular	Intracellular
1CO7I	Extracellular	Extracellular	Extracellular	Extracellular
1COEA	Extracellular	Extracellular	Extracellular	Extracellular
1CQHA	Extracellular	Extracellular	_	
1CR8A	Extracellular	Extracellular	Extracellular	Extracellular
1CRBA	Intracellular	Intracellular	_	
1CS6A	Intracellular	Extracellular	Extracellular	Extracellular
1CS8A	Intracellular	Extracellular	Extracellular	Extracellular
1CSEI	Extracellular	Extracellular	_	
1CSKA	Intracellular	Intracellular	_	
1CSYA	Intracellular	Intracellular	_	
1CTAA	Intracellular	Extracellular	_	
1CTQA	Intracellular	Intracellular	_	
1CVUA	Extracellular	Extracellular	Extracellular	Extracellular
1CWDL	Intracellular	Intracellular	_	
1CYDA	Intracellular	Intracellular	Intracellular	Intracellular
1CYOA	Intracellular	Extracellular	_	
1CZSA	Intracellular	Extracellular	Extracellular	Extracellular
1D0GR	Extracellular	Extracellular	_	
1D0NA	Intracellular	Intracellular	_	
1D1TA	Intracellular	Intracellular	_	
1D2EA	Intracellular	Intracellular	Intracellular	Intracellular
1D2LA	Extracellular	Extracellular	Extracellular	Extracellular
1D2SA	Intracellular	Extracellular	Extracellular	Extracellular
1D4TA	Extracellular	Intracellular	_	
1D4VA	Extracellular	Extracellular	_	
1D4XG	Intracellular	Intracellular	Extracellular	Intracellular
1D5MB	Intracellular	Extracellular	Extracellular	Extracellular
1D6BA	Extracellular	Extracellular	Extracellular	Extracellular

1D7PM	Intracellular	Extracellular	Extracellular	Extracellular
1D7QA	Intracellular	Intracellular	_	
1D9CA	Intracellular	Extracellular	Extracellular	Extracellular
1DANL	Extracellular	Extracellular	Extracellular	Extracellular
1DBHA	Intracellular	Intracellular	_	
1DDJA	Extracellular	Extracellular	Extracellular	Extracellular
1DECA	Extracellular	Extracellular	_	
1DEHA	Intracellular	Intracellular	_	
1DEMA	Extracellular	Extracellular	_	
1DEUA	Extracellular	Extracellular	Extracellular	Extracellular
1DFNA	Extracellular	Extracellular	Extracellular	Extracellular
1DG6A	Extracellular	Extracellular	Extracellular	Extracellular
1DGMA	Extracellular	Extracellular	_	
1DGUA	Intracellular	Intracellular	_	
1DJTA	Extracellular	Extracellular	Extracellular	Extracellular
1DL0A	Extracellular	Extracellular	_	
1DLEA	Extracellular	Extracellular	Extracellular	Extracellular
1DM5A	Intracellular	Intracellular	_	
1DO9A	Intracellular	Extracellular	_	
1DOKA	Extracellular	Extracellular	Extracellular	Extracellular
1DOTA	Extracellular	Intracellular	_	
1DP4A	Intracellular	Extracellular	Extracellular	Extracellular
1DPYA	Extracellular	Extracellular	Extracellular	Extracellular
1DQ7A	Extracellular	Extracellular	_	
1DQEA	Intracellular	Extracellular	Extracellular	Extracellular
1DROA	Intracellular	Intracellular	_	
1DS6A	Intracellular	Intracellular	_	
1DTDA	Intracellular	Extracellular	Extracellular	Extracellular
1DTKA	Extracellular	Extracellular	Extracellular	Extracellular
1DTLA	Intracellular	Intracellular	_	
1DTXA	Extracellular	Extracellular	_	
1DU9A	Extracellular	Extracellular	Extracellular	Extracellular
1DUGA	Intracellular	Intracellular	_	
1DV8A	Intracellular	Extracellular	_	
1DVPA	Intracellular	Intracellular	_	

1DX5I	Extracellular	Extracellular	Extracellular	Extracellular
1DY2A	Intracellular	Extracellular	Extracellular	Extracellular
1DY5A	Intracellular	Extracellular	Extracellular	Extracellular
1DYNA	Intracellular	Intracellular	_	
1DYTA	Intracellular	Extracellular	Extracellular	Extracellular
1DZKA	Intracellular	Intracellular	_	
1E0SA	Intracellular	Intracellular	_	
1E21A	Intracellular	Extracellular	Extracellular	Extracellular
1E27A	Intracellular	Intracellular	_	
1E3IA	Intracellular	Intracellular	_	
1E4QA	Extracellular	Extracellular	Extracellular	Extracellular
1E4RA	Extracellular	Extracellular	Extracellular	Extracellular
1E4TA	Extracellular	Extracellular	Extracellular	Extracellular
1E5PA	Intracellular	Extracellular	Extracellular	Extracellular
1E6GA	Intracellular	Intracellular	_	
1E6HA	Intracellular	Intracellular	_	
1E6WA	Intracellular	Intracellular	Intracellular	Intracellular
1E7WA	Intracellular	Intracellular	Intracellular	Intracellular
1E87A	Intracellular	Extracellular	_	
1E88A	Extracellular	Extracellular	Extracellular	Extracellular
1E8AA	Intracellular	Intracellular	_	
1E9LA	Intracellular	Extracellular	Extracellular	Extracellular
1E9TA	Extracellular	Extracellular	Extracellular	Extracellular
1EA5A	Extracellular	Extracellular	Extracellular	Extracellular
1EAIC	Extracellular	Extracellular	_	
1EALA	Intracellular	Intracellular	_	
1EAXA	Extracellular	Extracellular	_	
1EAZA	Intracellular	Intracellular	_	
1ECIA	Extracellular	Extracellular	_	
1ECIB	Extracellular	Extracellular	_	
1EDHA	Intracellular	Intracellular	Extracellular	Intracellular
1EDMB	Intracellular	Extracellular	Extracellular	Extracellular
1EDYA	Extracellular	Extracellular	Extracellular	Extracellular
1EEMA	Intracellular	Intracellular	_	
1EERA	Intracellular	Extracellular	Extracellular	Extracellular

1EERB	Intracellular	Extracellular	Extracellular	Extracellular
1EFEA	Extracellular	Extracellular	Extracellular	Extracellular
1EFNA	Intracellular	Intracellular	_	
1EGFA	Extracellular	Extracellular	Extracellular	Extracellular
1EGIA	Extracellular	Extracellular	Extracellular	Extracellular
1EGLA	Extracellular	Extracellular	_	
1EI9A	Intracellular	Extracellular	Extracellular	Extracellular
1EIHA	Intracellular	Extracellular	Extracellular	Extracellular
1EITA	Extracellular	Extracellular	_	
1EJ3A	Intracellular	Intracellular	_	
1EJMB	Extracellular	Extracellular	Extracellular	Extracellular
1EK6A	Intracellular	Intracellular	_	
1EKBB	Intracellular	Extracellular	Extracellular	Extracellular
1EL0A	Extracellular	Extracellular	Extracellular	Extracellular
1ELKA	Intracellular	Intracellular	_	
1ELTA	Extracellular	Intracellular	_	
1ELVA	Extracellular	Extracellular	Extracellular	Extracellular
1EM2A	Intracellular	Extracellular	Intracellular	Intracellular
1EMOA	Extracellular	Intracellular	Extracellular	Extracellular
1EMXA	Extracellular	Extracellular	_	
1EOTA	Intracellular	Extracellular	Extracellular	Extracellular
1EPAA	Intracellular	Extracellular	Extracellular	Extracellular
1EPFA	Intracellular	Intracellular	Extracellular	Intracellular
1EQ9A	Extracellular	Intracellular	_	
1ERDA	Extracellular	Extracellular	Extracellular	Extracellular
1ERPA	Extracellular	Extracellular	Extracellular	Extracellular
1ERYA	Extracellular	Extracellular	_	
1ESRA	Extracellular	Extracellular	Extracellular	Extracellular
1ETEA	Intracellular	Extracellular	Extracellular	Extracellular
1EUEA	Intracellular	Intracellular	_	
1EUFA	Intracellular	Extracellular	Extracellular	Extracellular
1EUOA	Intracellular	Extracellular	Extracellular	Extracellular
1EVSA	Intracellular	Extracellular	Extracellular	Extracellular
1EW2A	Intracellular	Intracellular	Extracellular	Intracellular
1EW3A	Intracellular	Extracellular	Extracellular	Extracellular

1EWFA	Intracellular	Extracellular	Extracellular	Extracellular
1EWKA	Intracellular	Extracellular	Extracellular	Extracellular
1EWSA	Extracellular	Extracellular	_	
1EXRA	Intracellular	Intracellular	_	
1EXSA	Intracellular	Extracellular	Extracellular	Extracellular
1EXTA	Extracellular	Extracellular	Extracellular	Extracellular
1EYOA	Intracellular	Extracellular	_	
1F14A	Intracellular	Intracellular	Intracellular	Intracellular
1F2FA	Intracellular	Intracellular	_	
1F2LA	Intracellular	Intracellular	Extracellular	Intracellular
1F2QA	Extracellular	Extracellular	Extracellular	Extracellular
1F3BA	Intracellular	Intracellular	_	
1F3KA	Extracellular		_	
1F3MC	Intracellular	Intracellular	_	
1F42A	Extracellular	Extracellular	Extracellular	Extracellular
1F5NA	Intracellular	Intracellular	_	
1F5YA	Extracellular	Intracellular	Extracellular	Extracellular
1F62A	Intracellular	Intracellular	_	
1F6BA	Intracellular	Intracellular	Extracellular	Intracellular
1F6FA	Intracellular	Extracellular	Extracellular	Extracellular
1F6RA	Intracellular	Extracellular	Extracellular	Extracellular
1F6WA	Intracellular	Extracellular	Extracellular	Extracellular
1F7EA	Extracellular	Extracellular	Extracellular	Extracellular
1F7ZA	Extracellular	Extracellular	Extracellular	Extracellular
1F81A	Intracellular	Intracellular	_	
1F8RA	Intracellular	Intracellular	Extracellular	Intracellular
1F8ZA	Extracellular	Intracellular	Extracellular	Intracellular
1F94A	Extracellular	Extracellular	_	
1F97A	Extracellular	Extracellular	Extracellular	Extracellular
1F9PA	Intracellular	Extracellular	Extracellular	Extracellular
1F9QA	Intracellular	Extracellular	Extracellular	Extracellular
1F9RA	Intracellular	Extracellular	Extracellular	Extracellular
1FAOA	Intracellular	Intracellular	_	
1FAQA	Intracellular	Intracellular	_	
1FASA	Extracellular	Extracellular	_	

1FBLA	Intracellular	Extracellular	Extracellular	Extracellular
1FBRA	Extracellular	Extracellular	Extracellular	Extracellular
1FCGA	Intracellular	Extracellular	Extracellular	Extracellular
1FD3A	Extracellular	Extracellular	Extracellular	Extracellular
1FDQA	Intracellular	Intracellular	_	
1FE0A	Intracellular	Extracellular	_	
1FE5A	Extracellular	Extracellular	Extracellular	Extracellular
1FE8H	Intracellular	Intracellular	_	
1FECA	Intracellular	Intracellular	_	
1FF4A	Extracellular	Extracellular	Extracellular	Extracellular
1FGKA	Intracellular	Extracellular	Extracellular	Extracellular
1FGYA	Intracellular	Intracellular	_	
1FH0A	Intracellular	Extracellular	Extracellular	Extracellular
1FHGA	Intracellular	Extracellular	_	
1FHOA	Intracellular	Intracellular	_	
1FHSA	Intracellular	Intracellular	_	
1FI5A	Intracellular	Intracellular	_	
1FI8A	Intracellular	Extracellular	Extracellular	Extracellular
1FIDA	Intracellular	Intracellular	Extracellular	Intracellular
1FIFA	Intracellular	Extracellular	Extracellular	Extracellular
1FJSA	Extracellular	Extracellular	Extracellular	Extracellular
1FJSL	Extracellular	Extracellular	Extracellular	Extracellular
1FKNA	Intracellular	Intracellular	Extracellular	Intracellular
1FKQA	Intracellular	Extracellular	Extracellular	Extracellular
1FLEI	Extracellular	Extracellular	Extracellular	Extracellular
1FLJA	Intracellular	Intracellular	_	
1FLTV	Extracellular	Extracellular	Extracellular	Extracellular
1FLTX	Intracellular	Intracellular	Extracellular	Intracellular
1FMKA	Intracellular	Intracellular	_	
1FMMS	Intracellular	Intracellular	_	
1FNAA	Extracellular	Extracellular	Extracellular	Extracellular
1FNFA	Extracellular	Extracellular	Extracellular	Extracellular
1FNGA	Intracellular	Intracellular	Extracellular	Intracellular
1FNLA	Extracellular	Extracellular	Extracellular	Extracellular
1FO3A	Intracellular	Extracellular	_	

1FONA	Extracellular	Intracellular	Intracellular	Intracellular
1FP0A	Intracellular	Intracellular	Intracellular	Intracellular
1FP5A	Extracellular	Intracellular	_	
1FPRA	Intracellular	Intracellular	_	
1FSUA	Intracellular	Intracellular	Extracellular	Intracellular
1FTPA	Intracellular	Intracellular	_	
1FU6A	Intracellular	Intracellular	_	
1FUJA	Intracellular	Extracellular	Extracellular	Extracellular
1FV1A	Intracellular	Extracellular	Extracellular	Extracellular
1FV1B	Intracellular	Extracellular	Extracellular	Extracellular
1FVRA	Intracellular	Extracellular	Extracellular	Extracellular
1FVUA	Extracellular	Extracellular	_	
1FVUB	Extracellular	Extracellular	_	
1FW1A	Intracellular	Intracellular	Intracellular	Intracellular
1FW4A	Intracellular	Intracellular	_	
1FXYA	Extracellular	Extracellular	Extracellular	Extracellular
1FYGA	Extracellular	Extracellular	Extracellular	Extracellular
1FYHA	Intracellular	Extracellular	Extracellular	Extracellular
1FYHB	Intracellular	Extracellular	Extracellular	Extracellular
1FZCB	Intracellular	Extracellular	Extracellular	Extracellular
1FZDA	Intracellular	Extracellular	Extracellular	Extracellular
1FZQA	Intracellular	Intracellular	_	
1FZVA	Intracellular	Extracellular	Extracellular	Extracellular
1G0XA	Intracellular	Extracellular	Extracellular	Extracellular
1G1CA	Intracellular		Extracellular	
1G1SA	Extracellular	Intracellular	Extracellular	Extracellular
1G1TA	Extracellular	Extracellular	Extracellular	Extracellular
1G2BA	Intracellular	Intracellular	_	
1G2TA	Intracellular	Extracellular	Extracellular	Extracellular
1G2XA	Extracellular	Extracellular	Extracellular	Extracellular
1G33A	Intracellular	Intracellular	_	
1G3MA	Intracellular	Intracellular	_	
1G4IA	Extracellular	Extracellular	Extracellular	Extracellular
1G5NA	Intracellular	Intracellular	_	
1G6MA	Extracellular	Extracellular	_	

1G6XA	Extracellular	Extracellular	Extracellular	Extracellular
1G7EA	Intracellular	Extracellular	Extracellular	Extracellular
1G8IA	Intracellular	Intracellular	_	
1G91A	Intracellular	Extracellular	Extracellular	Extracellular
1G96A	Intracellular	Extracellular	Extracellular	Extracellular
1G9PA	Extracellular	Extracellular	_	
1GBRA	Intracellular	Intracellular	_	
1GCQA	Intracellular	Intracellular	_	
1GCQC	Intracellular	Intracellular	_	
1GD6A	Intracellular	Extracellular	Extracellular	Extracellular
1GENA	Intracellular	Extracellular	Extracellular	Extracellular
1GGLA	Intracellular	Intracellular	_	
1GGXA	Intracellular	Intracellular	_	
1GGZA	Intracellular	Intracellular	_	
1GH2A	Intracellular	Intracellular	_	
1GHLA	Extracellular	Extracellular	Extracellular	Extracellular
1GHQC	Extracellular	Intracellular	Extracellular	Extracellular
1GJOA	Intracellular	Intracellular	Extracellular	Intracellular
1GK5A	Extracellular	Extracellular	Extracellular	Extracellular
1GKGA	Extracellular	Extracellular	Extracellular	Extracellular
1GKNA	Extracellular	Extracellular	Extracellular	Extracellular
1GL1I	Extracellular	Extracellular	Extracellular	Extracellular
1GL4B	Extracellular	Extracellular	Extracellular	Extracellular
1GL5A	Intracellular	Intracellular	_	
1GLQA	Intracellular	Intracellular	_	
1GM6A	Intracellular	Extracellular	Extracellular	Extracellular
1GMZA	Extracellular	Extracellular	_	
1GMZA	Extracellular	Intracellular	Intracellular	Intracellular
1GMZA	Extracellular	Extracellular	_	
1GMZA	Intracellular	Intracellular	_	
1GMZA	Intracellular	Intracellular	_	
1GMZA	Intracellular	Intracellular	_	
1GMZA	Intracellular	Intracellular	_	
1GMZA	Intracellular	Intracellular	_	
1GMZA	Intracellular	Intracellular	_	

1GMZA	Intracellular	Intracellular	_	
1GMZA	Intracellular	Intracellular	_	
1GMZA	Intracellular	Intracellular	_	
1GMZA	Intracellular	Intracellular	_	
1GMZA	Intracellular	Intracellular	_	
1GMZA	Intracellular	Intracellular	_	
1GMZA	Extracellular	Extracellular	Extracellular	Extracellular
1GP1A	Intracellular	Intracellular	Intracellular	Intracellular
1GPLA	Intracellular	Extracellular	Extracellular	Extracellular
1GQVA	Intracellular	Extracellular	Extracellular	Extracellular
1GR3A	Extracellular	Intracellular	Extracellular	Extracellular
1GSMA	Intracellular	Extracellular	Extracellular	Extracellular
1GSUA	Intracellular	Intracellular	_	
1GT1A	Intracellular	Intracellular	_	
1GV4A	Intracellular	Intracellular	Intracellular	Intracellular
1GV8A	Extracellular	Intracellular	_	
1GVKB	Extracellular	Extracellular	Extracellular	Extracellular
1GVZA	Intracellular	Extracellular	Extracellular	Extracellular
1GXEA	Intracellular	Intracellular	_	
1GXRA	Intracellular	Intracellular	Intracellular	Intracellular
1GZ2A	Extracellular	Extracellular	_	
1GZ6A	Intracellular	Intracellular	Intracellular	Intracellular
1GZ8A	Intracellular	Intracellular	_	
1GZKA	Intracellular	Intracellular	_	
1GZQA	Intracellular	Extracellular	Extracellular	Extracellular
1H03P	Extracellular	Extracellular	Extracellular	Extracellular
1H0DB	Extracellular	Intracellular	_	
1H0JA	Extracellular	Extracellular	Extracellular	Extracellular
1H0ZA	Intracellular	Extracellular	Extracellular	Extracellular
1H10A	Intracellular	Intracellular	_	
1H30A	Extracellular	Extracellular	Extracellular	Extracellular
1H3UA	Intracellular	Extracellular	Intracellular	Intracellular
1H45A	Extracellular	Extracellular	Extracellular	Extracellular
1H4WA	Extracellular	Intracellular	_	
1H59B	Extracellular	Extracellular	Extracellular	Extracellular

1H6RA	Intracellular	Intracellular	_	
1H76A	Extracellular	Intracellular	_	
1H8PA	Extracellular	Extracellular	Extracellular	Extracellular
1H8UA	Extracellular	Extracellular	Extracellular	Extracellular
1H8XA	Intracellular	Extracellular	Extracellular	Extracellular
1H92A	Intracellular	Intracellular	_	
1H95A	Intracellular	Intracellular	_	
1HA6A	Intracellular	Extracellular	Extracellular	Extracellular
1HAEA	Intracellular	Intracellular	_	
1HC9A	Extracellular	Extracellular	Extracellular	Extracellular
1HCBA	Intracellular	Intracellular	_	
1HCCA	Extracellular	Extracellular	Extracellular	Extracellular
1HD2A	Intracellular	Intracellular	Intracellular	Intracellular
1HD6A	Extracellular	Extracellular	_	
1HD7A	Intracellular	Intracellular	_	
1HDLA	Intracellular	Extracellular	Extracellular	Extracellular
1HDMA	Extracellular	Extracellular	Extracellular	Extracellular
1HDMB	Extracellular	Extracellular	Extracellular	Extracellular
1HDOA	Intracellular	Intracellular	_	
1HDRA	Intracellular	Intracellular	Intracellular	Intracellular
1HE7A	Intracellular	Intracellular	Extracellular	Extracellular
1HETA	Intracellular	Intracellular	_	
1HFIA	Extracellular	Extracellular	Extracellular	Extracellular
1HFXA	Intracellular	Extracellular	Extracellular	Extracellular
1HGUA	Intracellular	Extracellular	Extracellular	Extracellular
1HHLA	Extracellular	Extracellular	_	
1HI7A	Extracellular	Extracellular	Extracellular	Extracellular
1HICA	Intracellular	Extracellular	_	
1HJ7A	Extracellular	Intracellular	Extracellular	Extracellular
1HJ8A	Extracellular	Extracellular	Extracellular	Extracellular
1HJ9A	Extracellular	Extracellular	Extracellular	Extracellular
1HJXA	Intracellular	Extracellular	Extracellular	Extracellular
1HKKA	Intracellular	Extracellular	Extracellular	Extracellular
1HKOA	Intracellular	Extracellular	_	
1HLYA	Extracellular	Extracellular	_	

1HM6A	Intracellular	Intracellular	_	
1HMCA	Intracellular	Extracellular	Extracellular	Extracellular
1HMSA	Intracellular	Intracellular	_	
1HN4A	Extracellular	Extracellular	Extracellular	Extracellular
1HNAA	Intracellular	Intracellular	_	
1HNFA	Intracellular	Intracellular	Extracellular	Intracellular
1HP2A	Extracellular	Extracellular	_	
1HPLA	Extracellular	Intracellular	Extracellular	Extracellular
1HPTA	Extracellular	Extracellular	Extracellular	Extracellular
1HQ8A	Extracellular	Extracellular	_	
1HRNA	Extracellular	Extracellular	Extracellular	Extracellular
1HSBA	Extracellular	Extracellular	Extracellular	Extracellular
1HSOA	Intracellular	Intracellular	_	
1HSQA	Intracellular	Intracellular	_	
1HT0A	Intracellular	Intracellular	_	
1HULA	Intracellular	Extracellular	Extracellular	Extracellular
1HUMA	Extracellular	Extracellular	Extracellular	Extracellular
1HUPA	Intracellular	Extracellular	Extracellular	Extracellular
1HUQA	Intracellular	Intracellular	_	
1HUWA	Intracellular	Extracellular	Extracellular	Extracellular
1HVDA	Intracellular	Intracellular	_	
1HX0A	Extracellular	Extracellular	Extracellular	Extracellular
1HX1B	Intracellular	Intracellular	_	
1HXNA	Extracellular	Extracellular	Extracellular	Extracellular
1HY0A	Intracellular	Intracellular	_	
1HYKA	Extracellular	Extracellular	Extracellular	Extracellular
1HZFA	Intracellular	Extracellular	Extracellular	Extracellular
1HZIA	Intracellular	Extracellular	Extracellular	Extracellular
1I07A	Intracellular	Intracellular	_	
1I0AA	Intracellular	Intracellular	_	
1I0ZA	Intracellular	Intracellular	_	
1I10A	Intracellular	Intracellular	_	
1I1IP	Intracellular	Intracellular	Intracellular	Intracellular
1I1JA	Intracellular	Extracellular	Extracellular	Extracellular
1I1NA	Intracellular	Intracellular	_	

1I1RA	Extracellular	Intracellular	Extracellular	Extracellular
1I25A	Extracellular	Extracellular	Extracellular	Extracellular
1I26A	Extracellular	Extracellular	_	
1I2MA	Intracellular	Intracellular	_	
1I2UA	Extracellular	Extracellular	_	
1I3ZA	Extracellular	Extracellular	_	
1I4AA	Intracellular	Intracellular	_	
1I4FA	Intracellular	Extracellular	Extracellular	Extracellular
1I6GA	Extracellular	Extracellular	_	
1I6ZA	Intracellular	Intracellular	Intracellular	Intracellular
1I71A	Extracellular	Extracellular	Extracellular	Extracellular
1I7PA	Intracellular	Intracellular	Extracellular	Intracellular
1IAKA	Intracellular	Extracellular	Extracellular	Extracellular
1IAKB	Extracellular	Extracellular	Extracellular	Extracellular
1IAMA	Intracellular	Extracellular	Extracellular	Extracellular
1IARB	Intracellular	Extracellular	Extracellular	Extracellular
1IAUA	Intracellular	Extracellular	Extracellular	Extracellular
1ICAA	Intracellular	Extracellular	Extracellular	Extracellular
1ICCC	Intracellular	Intracellular	_	
1IE5A	Intracellular	Extracellular	Extracellular	Extracellular
1IEJA	Extracellular	Extracellular	Extracellular	Extracellular
1IFCA	Intracellular	Intracellular	_	
1IGLA	Intracellular	Extracellular	Extracellular	Extracellular
1IH0A	Intracellular	Intracellular	_	
1IHKA	Extracellular	Extracellular	_	
1IILE	Intracellular	Intracellular	Extracellular	Intracellular
1IIUA	Intracellular	Extracellular	Extracellular	Extracellular
1IIZA	Extracellular	Extracellular	_	
1IJBA	Extracellular	Extracellular	Extracellular	Extracellular
1IJQA	Extracellular	Intracellular	Extracellular	Extracellular
1IJTA	Intracellular	Extracellular	Extracellular	Extracellular
1IJXA	Extracellular	Extracellular	Extracellular	Extracellular
1IJYA	Extracellular	Extracellular	Extracellular	Extracellular
1IJZA	Extracellular	Extracellular	Extracellular	Extracellular
1ILR1	Intracellular	Extracellular	Extracellular	Extracellular

1IMTA	Extracellular	Extracellular	_	
1IMXA	Intracellular	Extracellular	Extracellular	Extracellular
1IODA	Intracellular	Extracellular	Extracellular	Extracellular
1IODB	Extracellular	Extracellular	Extracellular	Extracellular
1IODG	Extracellular	Extracellular	Extracellular	Extracellular
1IOXA	Extracellular	Extracellular	Extracellular	Extracellular
1IQ7A	Extracellular	Extracellular	Extracellular	Extracellular
1IQ9A	Extracellular	Extracellular	_	
1IQAA	Intracellular	Extracellular	_	
1IR3A	Intracellular	Extracellular	Extracellular	Extracellular
1IRHA	Extracellular	Extracellular	Extracellular	Extracellular
1IRJA	Intracellular	Intracellular	_	
1ITVA	Extracellular	Extracellular	Extracellular	Extracellular
1IVMA	Extracellular	Extracellular	Extracellular	Extracellular
1IVYA	Intracellular	Extracellular	Extracellular	Extracellular
1IW4A	Extracellular	Extracellular	_	
1IXXA	Extracellular	Extracellular	Extracellular	Extracellular
1IY6A	Extracellular	Extracellular	_	
1IYHA	Intracellular	Intracellular	_	
1J0SA	Intracellular	Intracellular	_	
1J2LA	Extracellular	Extracellular	_	
1J34A	Extracellular	Extracellular	_	
1J34B	Intracellular	Extracellular	Extracellular	Extracellular
1J34C	Extracellular	Intracellular	_	
1J37A	Intracellular	Extracellular	Extracellular	Extracellular
1J55A	Intracellular	Intracellular	_	
1J5JA	Extracellular	Extracellular	Extracellular	Extracellular
1J72A	Intracellular	Intracellular	_	
1J7MA	Intracellular	Extracellular	Extracellular	Extracellular
1J7OA	Intracellular	Intracellular	_	
1J7QA	Intracellular	Intracellular	_	
1J7RA	Intracellular	Intracellular	_	
1J8EA	Extracellular	Extracellular	Extracellular	Extracellular
1J8HD	Extracellular	Extracellular	Extracellular	Extracellular
1J8IA	Extracellular	Extracellular	Extracellular	Extracellular

1J8KA	Extracellular	Extracellular	Extracellular	Extracellular
1J99A	Intracellular	Intracellular	_	
1JAEA	Extracellular	Intracellular	_	
1JBAA	Intracellular	Intracellular	_	
1JBJA	Extracellular	Extracellular	Extracellular	Extracellular
1JBUL	Extracellular	Extracellular	Extracellular	Extracellular
1JC2A	Intracellular	Intracellular	_	
1JC6A	Intracellular	Extracellular	_	
1JC9A	Intracellular	Intracellular	_	
1JD0A	Intracellular	Extracellular	Extracellular	Extracellular
1JDPA	Intracellular	Extracellular	Extracellular	Extracellular
1JE6A	Intracellular	Extracellular	Extracellular	Extracellular
1JEXA	Intracellular	Extracellular	_	
1JF0A	Intracellular	Extracellular	_	
1JFKA	Intracellular	Intracellular	_	
1JFNA	Extracellular	Extracellular	Extracellular	Extracellular
1JGKA	Extracellular	Extracellular	Extracellular	Extracellular
1JIAA	Extracellular	Extracellular	Extracellular	Extracellular
1JK4A	Extracellular	Extracellular	Extracellular	Extracellular
1JK8A	Extracellular	Intracellular	_	
1JK8B	Extracellular	Intracellular	Extracellular	Extracellular
1JKSA	Intracellular	Extracellular	_	
1JLIA	Intracellular	Intracellular	Extracellular	Intracellular
1JLNA	Intracellular	Extracellular	Extracellular	Extracellular
1JLTA	Extracellular	Extracellular	_	
1JLTB	Extracellular	Extracellular	_	
1JNDA	Intracellular	Extracellular	Extracellular	Extracellular
1JPAA	Extracellular	Extracellular	Extracellular	Extracellular
1JQPA	Extracellular	Extracellular	Extracellular	Extracellular
1JQZA	Intracellular	Intracellular	_	
1JRFA	Extracellular	Extracellular	Extracellular	Extracellular
1JSEA	Extracellular	Extracellular	Extracellular	Extracellular
1JSFA	Extracellular	Extracellular	Extracellular	Extracellular
1JSSA	Intracellular	Intracellular	_	
1JTVA	Intracellular	Intracellular	Intracellular	Intracellular

1JU5A	Intracellular	Intracellular	_	
1JUFA	Intracellular	Extracellular	Extracellular	Extracellular
1JUGA	Extracellular	Extracellular	_	
1JUQA	Intracellular	Intracellular	_	
1JWDA	Intracellular	Intracellular	_	
1JWOA	Intracellular	Intracellular	Intracellular	Intracellular
1JWYB	Intracellular	Intracellular	_	
1JYDA	Intracellular	Extracellular	Extracellular	Extracellular
1JYRA	Intracellular	Intracellular	_	
1JZAA	Extracellular	Extracellular	Extracellular	Extracellular
1JZNA	Extracellular	Extracellular	_	
1JZUA	Intracellular	Extracellular	Extracellular	Extracellular
1K0MA	Intracellular	Intracellular	_	
1K1ZA	Intracellular	Intracellular	_	
1K2DA	Intracellular	Intracellular	_	
1K2DB	Extracellular	Extracellular	Extracellular	Extracellular
1K2HA	Intracellular	Intracellular	_	
1K2PA	Intracellular	Intracellular	_	
1K36A	Extracellular	Extracellular	Extracellular	Extracellular
1K3BA	Extracellular	Extracellular	Extracellular	Extracellular
1K3YA	Intracellular	Intracellular	_	
1K4US	Intracellular	Intracellular	_	
1K4YA	Intracellular	Extracellular	Extracellular	Extracellular
1K59A	Intracellular	Extracellular	Extracellular	Extracellular
1K5NA	Extracellular	Extracellular	Extracellular	Extracellular
1K5NB	Intracellular	Extracellular	Extracellular	Extracellular
1K7BA	Extracellular	Extracellular	Extracellular	Extracellular
1K7WA	Intracellular	Intracellular	_	
1K8DA	Intracellular	Intracellular	Extracellular	Intracellular
1K8KC	Intracellular	Intracellular	_	
1K8UA	Intracellular	Intracellular	_	
1K9AA	Intracellular	Intracellular	_	
1K9IA	Extracellular	Intracellular	_	
1K9JA	Extracellular	Intracellular	_	
1KAOA	Intracellular	Intracellular	_	

1KBAA	Extracellular	Extracellular	Extracellular	Extracellular
1KBFA	Intracellular	Intracellular	_	
1KBSA	Extracellular	Extracellular	Extracellular	Extracellular
1KCQA	Intracellular	Intracellular	Extracellular	Extracellular
1KCUH	Extracellular	Intracellular	_	
1KCVH	Extracellular	Extracellular	Extracellular	Extracellular
1KCVL	Intracellular	Extracellular	_	
1KDUA	Extracellular	Extracellular	Extracellular	Extracellular
1KEQA	Intracellular	Intracellular	Intracellular	Intracellular
1KEXA	Intracellular	Intracellular	Extracellular	Intracellular
1KGDA	Intracellular	Intracellular	_	
1KI0A	Extracellular	Extracellular	Extracellular	Extracellular
1KI1B	Intracellular	Intracellular	_	
1KJ6A	Extracellular	Extracellular	Extracellular	Extracellular
1KJSA	Intracellular	Intracellular	Extracellular	Intracellular
1KJVB	Intracellular	Intracellular	_	
1KJWA	Intracellular	Intracellular	_	
1KL9A	Intracellular	Intracellular	_	
1KLCA	Intracellular	Extracellular	Extracellular	Extracellular
1KLIH	Extracellular	Extracellular	Extracellular	Extracellular
1KLIL	Extracellular	Extracellular	Extracellular	Extracellular
1KLOA	Extracellular	Extracellular	Extracellular	Extracellular
1KLUB	Intracellular	Extracellular	Extracellular	Extracellular
1KMAA	Extracellular	Extracellular	_	
1KMQA	Intracellular	Intracellular	_	
1KOEA	Intracellular	Extracellular	Extracellular	Extracellular
1KOIA	Extracellular	Extracellular	Extracellular	Extracellular
1KRNA	Extracellular	Extracellular	Extracellular	Extracellular
1KS0A	Intracellular	Extracellular	Extracellular	Extracellular
1KSHA	Intracellular	Intracellular	_	
1KSOA	Extracellular	Extracellular	_	
1KSQA	Extracellular	Extracellular	Extracellular	Extracellular
1KT7A	Intracellular	Extracellular	_	
1KTBA	Intracellular	Intracellular	_	
1KTEA	Intracellular	Intracellular	_	

1KTHA	Intracellular	Extracellular	Extracellular	Extracellular
1KTJA	Intracellular	Extracellular	Extracellular	Extracellular
1KTZA	Intracellular	Extracellular	Extracellular	Extracellular
1KUFA	Intracellular	Extracellular	Extracellular	Extracellular
1KV0A	Extracellular	Extracellular	_	
1KVJA	Intracellular	Extracellular	_	
1KWMA	Intracellular	Extracellular	Extracellular	Extracellular
1KXGA	Intracellular	Intracellular	_	
1KXIA	Extracellular	Extracellular	Extracellular	Extracellular
1KXOA	Extracellular	Extracellular	Extracellular	Extracellular
1KXPD	Extracellular	Extracellular	Extracellular	Extracellular
1KZ7A	Intracellular	Intracellular	_	
1KZQA	Extracellular	Extracellular	Intracellular	Extracellular
1KZWA	Intracellular	Intracellular	_	
1L2HA	Extracellular	Intracellular	_	
1L5CA	Extracellular	Extracellular	Extracellular	Extracellular
1L6JA	Extracellular	Extracellular	Extracellular	Extracellular
1L6MA	Intracellular	Extracellular	Extracellular	Extracellular
1L6XA	Intracellular	Extracellular	Intracellular	Intracellular
1L8CA	Intracellular	Intracellular	_	
1L9LA	Extracellular	Extracellular	Extracellular	Extracellular
1LARA	Extracellular	Extracellular	Extracellular	Extracellular
1LARB	Extracellular	Extracellular	Extracellular	Extracellular
1LCKA	Intracellular	Intracellular	_	
1LCYA	Intracellular	Intracellular	Intracellular	Intracellular
1LD6A	Extracellular	Extracellular	Extracellular	Extracellular
1LD9A	Intracellular	Extracellular	Extracellular	Extracellular
1LDLA	Extracellular	Intracellular	Extracellular	Extracellular
1LDTL	Extracellular	Extracellular	_	
1LE6A	Extracellular	Extracellular	Extracellular	Extracellular
1LF2A	Intracellular	Intracellular	_	
1LF7A	Intracellular	Extracellular	Extracellular	Extracellular
1LFOA	Intracellular	Intracellular	_	
1LI1A	Extracellular	Extracellular	Extracellular	Extracellular
1LI1C	Extracellular	Extracellular	Extracellular	Extracellular

1LIDA	Intracellular	Intracellular	_	
1LIRA	Extracellular	Extracellular	Extracellular	Extracellular
1LITA	Extracellular	Extracellular	Extracellular	Extracellular
1LJ0A	Intracellular	Intracellular	_	
1LK2A	Intracellular	Extracellular	Extracellular	Extracellular
1LK2B	Extracellular	Extracellular	Extracellular	Extracellular
1LK3A	Intracellular	Extracellular	Extracellular	Extracellular
1LK3H	Extracellular	Intracellular	_	
1LK3L	Intracellular	Intracellular	_	
1LKIA	Intracellular	Extracellular	Extracellular	Extracellular
1LKKA	Intracellular	Intracellular	_	
1LMJA	Extracellular	Intracellular	Extracellular	Extracellular
1LMMA	Extracellular	Extracellular	_	
1LMQA	Extracellular	Extracellular	Extracellular	Extracellular
1LMRA	Extracellular	Extracellular	_	
1LN1A	Intracellular	Intracellular	_	
1LNUB	Extracellular	Extracellular	Extracellular	Extracellular
1LO6A	Extracellular	Extracellular	Extracellular	Extracellular
1LP9E	Extracellular	Intracellular	_	
1LPBA	Extracellular	Extracellular	Extracellular	Extracellular
1LPBB	Intracellular	Extracellular	Extracellular	Extracellular
1LPJA	Intracellular	Intracellular	_	
1LQIA	Extracellular	Extracellular	Extracellular	Extracellular
1LQQA	Extracellular	Extracellular	_	
1LQVA	Intracellular	Extracellular	Extracellular	Extracellular
1LR7A	Extracellular	Extracellular	Extracellular	Extracellular
1LS6A	Intracellular	Intracellular	_	
1LSIA	Extracellular	Extracellular	Extracellular	Extracellular
1LSLA	Extracellular	Extracellular	Extracellular	Extracellular
1LTOA	Intracellular	Extracellular	Extracellular	Extracellular
1LUFA	Extracellular	Extracellular	Extracellular	Extracellular
1LUIA	Intracellular	Intracellular	_	
1LVGA	Intracellular	Intracellular	Intracellular	Intracellular
1LWRA	Intracellular	Intracellular	Extracellular	Intracellular
1LXIA	Intracellular	Intracellular	Extracellular	Intracellular

1LY2A	Extracellular	Intracellular	Extracellular	Extracellular
1M07A	Extracellular	Extracellular	Extracellular	Extracellular
1M0UA	Intracellular	Intracellular	_	
1M12A	Extracellular	Extracellular	Extracellular	Extracellular
1M2IA	Intracellular	Extracellular	_	
1M2SA	Extracellular	Extracellular	Extracellular	Extracellular
1M31A	Intracellular	Extracellular	_	
1M39A	Intracellular	Intracellular	Intracellular	Intracellular
1M48A	Extracellular	Extracellular	Extracellular	Extracellular
1M4KA	Extracellular	Extracellular	Extracellular	Extracellular
1M4LA	Extracellular	Extracellular	Extracellular	Extracellular
1M4RA	Intracellular	Extracellular	Extracellular	Extracellular
1M61A	Intracellular	Intracellular	_	
1M62A	Intracellular	Intracellular	_	
1M66A	Intracellular	Intracellular	_	
1M6DA	Intracellular	Extracellular	Extracellular	Extracellular
1M6HA	Intracellular	Intracellular	_	
1M6IA	Intracellular	Intracellular	Intracellular	Intracellular
1M6OA	Extracellular	Extracellular	Extracellular	Extracellular
1M7BA	Intracellular	Extracellular	_	
1M8RA	Extracellular	Extracellular	_	
1M8TA	Extracellular	Extracellular	Extracellular	Extracellular
1M9ZA	Intracellular	Intracellular	Extracellular	Intracellular
1MAIA	Intracellular	Intracellular	_	
1MB6A	Extracellular	Extracellular	Extracellular	Extracellular
1MC2A	Extracellular	Extracellular	Extracellular	Extracellular
1MCXA	Intracellular	Intracellular	_	
1MD6A	Extracellular	Extracellular	_	
1MDCA	Intracellular	Intracellular	_	
1ME4A	Extracellular	Extracellular	Extracellular	Extracellular
1MEKA	Intracellular	Extracellular	Extracellular	Extracellular
1MEMA	Extracellular	Extracellular	Extracellular	Extracellular
1MEXH	Extracellular	Intracellular	_	
1MF7A	Intracellular	Extracellular	Extracellular	Extracellular
1MG5A	Intracellular	Intracellular	_	

1MH1A	Intracellular	Intracellular	_	
1MH5B	Extracellular	Extracellular	_	
1MH5B	Extracellular	Intracellular	Intracellular	Intracellular
1MH5B	Extracellular	Extracellular	_	
1MH5B	Intracellular	Intracellular	_	
1MH5B	Intracellular	Intracellular	_	
1MH5B	Intracellular	Intracellular	_	
1MH5B	Intracellular	Intracellular	_	
1MH5B	Intracellular	Intracellular	_	
1MH5B	Intracellular	Intracellular	_	
1MH5B	Intracellular	Intracellular	_	
1MH5B	Intracellular	Intracellular	_	
1MH5B	Intracellular	Intracellular	_	
1MH5B	Intracellular	Intracellular	_	
1MH5B	Intracellular	Intracellular	_	
1MH5B	Intracellular	Intracellular	_	
1MH5B	Extracellular	Extracellular	Extracellular	Extracellular
1MH5H	Intracellular	Intracellular	_	
1MH8A	Extracellular	Extracellular	_	
1MHOA	Intracellular	Intracellular	_	
1MHQA	Intracellular	Intracellular	Extracellular	Intracellular
1MI2A	Extracellular	Extracellular	Extracellular	Extracellular
1MJ4A	Extracellular	Intracellular	Intracellular	Intracellular
1MJUH	Intracellular	Intracellular	_	
1MLDA	Intracellular	Intracellular	Intracellular	Intracellular
1MM0A	Intracellular	Extracellular	Intracellular	Intracellular
1MM2A	Intracellular	Intracellular	_	
1MM3A	Intracellular	Intracellular	_	
1MOUA	Intracellular	Intracellular	_	
1MOXA	Extracellular	Extracellular	Extracellular	Extracellular
1MOXC	Intracellular	Extracellular	Extracellular	Extracellular
1MP8A	Intracellular	Intracellular	_	
1MPZA	Extracellular	Extracellular	_	
1MQ4A	Intracellular	Intracellular	_	
1MQBA	Intracellular	Extracellular	Extracellular	Extracellular

1MQIA	Intracellular	Extracellular	Extracellular	Extracellular
1MR8A	Intracellular	Intracellular	_	
1MS6A	Intracellular	Extracellular	Extracellular	Extracellular
1MSGA	Intracellular	Extracellular	Extracellular	Extracellular
1MTXA	Extracellular	Extracellular	_	
1MUJA	Intracellular	Extracellular	Extracellular	Extracellular
1MUJB	Extracellular	Extracellular	Extracellular	Extracellular
1MVGA	Intracellular	Intracellular	_	
1MVIA	Extracellular	Extracellular	Extracellular	Extracellular
1MVJA	Extracellular	Extracellular	Extracellular	Extracellular
1MW4A	Intracellular	Intracellular	_	
1MX1A	Intracellular	Extracellular	Extracellular	Extracellular
1MXEA	Intracellular	Intracellular	_	
1MYNA	Extracellular	Extracellular	Extracellular	Extracellular
1MZAA	Intracellular	Extracellular	Extracellular	Extracellular
1N0SA	Extracellular	Extracellular	Extracellular	Extracellular
1N0YA	Intracellular	Intracellular	_	
1N0YB	Intracellular	Intracellular	_	
1N1FA	Intracellular	Extracellular	Extracellular	Extracellular
1N1XA	Intracellular	Extracellular	Extracellular	Extracellular
1N26A	Intracellular	Extracellular	Extracellular	Extracellular
1N28A	Intracellular	Extracellular	Extracellular	Extracellular
1N3LA	Intracellular	Intracellular	_	
1N3YA	Intracellular	Extracellular	Extracellular	Extracellular
1N4YA	Intracellular	Extracellular	Extracellular	Extracellular
1N5DA	Intracellular	Intracellular	Intracellular	Intracellular
1N5MA	Intracellular	Extracellular	Extracellular	Extracellular
1N5UA	Extracellular	Extracellular	Extracellular	Extracellular
1N6UA	Intracellular	Extracellular	Extracellular	Extracellular
1N8YC	Extracellular	Extracellular	Extracellular	Extracellular
1N9DA	Intracellular	Extracellular	Extracellular	Extracellular
1NA7A	Extracellular	Intracellular	_	
1NB5I	Intracellular	Intracellular	_	
1NCFA	Extracellular	Extracellular	Extracellular	Extracellular
1NCFB	Extracellular	Extracellular	Extracellular	Extracellular

1NCIA	Intracellular	Intracellular	Extracellular	Intracellular
1NCUA	Intracellular		Extracellular	
1NCXA	Intracellular	Intracellular	_	
1ND1A	Intracellular	Extracellular	Extracellular	Extracellular
1ND6A	Extracellular	Extracellular	Extracellular	Extracellular
1NDHA	Intracellular	Intracellular	_	
1NEGA	Intracellular	Intracellular	_	
1NEPA	Extracellular	Extracellular	Extracellular	Extracellular
1NG2A	Intracellular	Intracellular	_	
1NIXA	Extracellular	Extracellular	_	
1NIYA	Extracellular	Extracellular	_	
1NKLA	Extracellular	Extracellular	_	
1NKRA	Extracellular	Extracellular	Extracellular	Extracellular
1NKXA	Extracellular	Extracellular	Extracellular	Extracellular
1NL1A	Extracellular	Extracellular	Extracellular	Extracellular
1NLOC	Intracellular	Intracellular	_	
1NMMA	Intracellular	Extracellular	Extracellular	Extracellular
1NN5A	Intracellular	Intracellular	Intracellular	Intracellular
1NN6A	Intracellular	Extracellular	Extracellular	Extracellular
1NOWA	Intracellular	Intracellular	Extracellular	Intracellular
1NP1A	Extracellular	Extracellular	Extracellular	Extracellular
1NPEA	Extracellular	Extracellular	Extracellular	Extracellular
1NPIA	Extracellular	Extracellular	Extracellular	Extracellular
1NPMA	Extracellular	Extracellular	Extracellular	Extracellular
1NQNA	Extracellular	Extracellular	Extracellular	Extracellular
1NR0A	Intracellular	Intracellular	_	
1NRAA	Extracellular	Extracellular	_	
1NRVA	Intracellular	Intracellular	_	
1NSAA	Intracellular	Extracellular	Extracellular	Extracellular
1NSHA	Intracellular	Intracellular	_	
1NSTA	Intracellular	Intracellular	Extracellular	Intracellular
1NTNA	Extracellular	Extracellular	_	
1NTXA	Extracellular	Extracellular	_	
1NU6A	Extracellular	Extracellular	Extracellular	Extracellular
1NVRA	Intracellular	Intracellular	_	

1NXCA	Intracellular	Intracellular	_	
1NZIA	Extracellular	Extracellular	Extracellular	Extracellular
1O1VA	Intracellular	Intracellular	_	
1O4RA	Intracellular	Intracellular	_	
1O6SB	Intracellular	Intracellular	Extracellular	Intracellular
1O6XA	Intracellular	Extracellular	Extracellular	Extracellular
1O73A	Intracellular	Intracellular	_	
1O7BT	Intracellular	Extracellular	Extracellular	Extracellular
1O7ZA	Extracellular	Extracellular	Extracellular	Extracellular
1O80A	Extracellular	Extracellular	Extracellular	Extracellular
1O8VA	Intracellular	Intracellular	_	
1O91A	Extracellular	Intracellular	Extracellular	Extracellular
1O9AA	Extracellular	Extracellular	Extracellular	Extracellular
1OAAA	Intracellular	Extracellular	_	
1OAWA	Extracellular	Extracellular	_	
1OB3A	Intracellular	Intracellular	_	
1OC4A	Intracellular	Extracellular	Extracellular	Extracellular
1OE0A	Intracellular	Intracellular	_	
1OE8A	Intracellular	Intracellular	_	
1OE9B	Intracellular	Intracellular	_	
1OEBA	Intracellular	Intracellular	_	
1OGAD	Extracellular	Extracellular	_	
1OGAE	Intracellular	Intracellular	_	
1OGSA	Intracellular	Extracellular	_	
1OHTA	Intracellular	Extracellular	Extracellular	Extracellular
1OIVA	Intracellular	Extracellular	_	
1OK3A	Extracellular	Extracellular	Extracellular	Extracellular
1OKQA	Extracellular	Extracellular	Extracellular	Extracellular
1OKTA	Intracellular	Intracellular	_	
1OLZA	Intracellular	Extracellular	Extracellular	Extracellular
1OMBA	Intracellular	Extracellular	Extracellular	Extracellular
1OMCA	Extracellular	Extracellular	Extracellular	Extracellular
1OMRA	Intracellular	Intracellular	_	
1OMWA	Intracellular	Intracellular	_	
1OMYA	Extracellular	Extracellular	Extracellular	Extracellular

1ONCA	Intracellular	Extracellular	_	
1ONJA	Extracellular	Extracellular	Extracellular	Extracellular
1ONQA	Intracellular	Extracellular	Extracellular	Extracellular
1OO9B	Extracellular	Extracellular	Extracellular	Extracellular
1OOHA	Intracellular	Extracellular	Extracellular	Extracellular
1OP4A	Intracellular	Intracellular	Extracellular	Intracellular
1OPBA	Intracellular	Intracellular	_	
1OPJA	Intracellular	Intracellular	_	
1OPKA	Intracellular	Intracellular	_	
1OQCA	Intracellular	Extracellular	_	
1OQEK	Intracellular	Intracellular	_	
1OQEN	Intracellular	Intracellular	_	
1ORFA	Intracellular	Extracellular	Extracellular	Extracellular
1ORVA	Extracellular	Extracellular	Extracellular	Extracellular
1OSPH	Intracellular	Intracellular	_	
1OSXA	Intracellular	Intracellular	_	
1OV3A	Intracellular	Intracellular	_	
1OVNA	Intracellular	Extracellular	Extracellular	Extracellular
1OWSA	Extracellular	Extracellular	_	
1OWSB	Extracellular	Extracellular	_	
1OWWA	Extracellular	Extracellular	Extracellular	Extracellular
1OZNA	Intracellular	Intracellular	Extracellular	Intracellular
1OZUA	Intracellular	Intracellular	_	
1P0AA	Extracellular	Extracellular	_	
1P0CA	Intracellular	Intracellular	_	
1P0IA	Intracellular	Extracellular	Extracellular	Extracellular
1P15A	Intracellular	Intracellular	Extracellular	Intracellular
1P4OA	Intracellular	Extracellular	Extracellular	Extracellular
1P4QB	Intracellular	Intracellular	_	
1P57B	Extracellular	Extracellular	Extracellular	Extracellular
1P5ZB	Intracellular	Intracellular	_	
1P6PA	Intracellular	Intracellular	_	
1P8XA	Intracellular	Intracellular	Extracellular	Intracellular
1P9AG	Intracellular	Extracellular	Extracellular	Extracellular
1P9JA	Extracellular	Intracellular	Extracellular	Extracellular

1PA1A	Intracellular	Intracellular	_	
1PB7A	Intracellular	Extracellular	Extracellular	Extracellular
1PBAA	Intracellular	Extracellular	Extracellular	Extracellular
1PCEA	Intracellular	Extracellular	Extracellular	Extracellular
1PCNA	Extracellular	Extracellular	Extracellular	Extracellular
1PD21	Intracellular	Intracellular	_	
1PFNA	Intracellular	Extracellular	Extracellular	Extracellular
1PFZA	Intracellular	Intracellular	_	
1PHKA	Intracellular	Intracellular	_	
1PHTA	Intracellular	Intracellular	_	
1PICA	Intracellular	Intracellular	_	
1PK6A	Extracellular	Extracellular	Extracellular	Extracellular
1PK6B	Extracellular	Extracellular	Extracellular	Extracellular
1PK6C	Extracellular	Extracellular	Extracellular	Extracellular
1PL7A	Intracellular	Intracellular	_	
1PLFA	Intracellular	Extracellular	_	
1PLOA	Intracellular	Intracellular	Extracellular	Intracellular
1PLSA	Intracellular	Intracellular	_	
1PMLA	Extracellular	Extracellular	Extracellular	Extracellular
1PMQA	Intracellular	Intracellular	_	
1PMXA	Intracellular	Extracellular	Extracellular	Extracellular
1PN9A	Intracellular	Intracellular	_	
1PNHA	Extracellular	Extracellular	Intracellular	Extracellular
1POAA	Extracellular	Extracellular	Extracellular	Extracellular
1PP2L	Extracellular	Extracellular	Extracellular	Extracellular
1PPFE	Intracellular	Extracellular	Extracellular	Extracellular
1PR9A	Intracellular	Intracellular	Intracellular	Intracellular
1PRXA	Intracellular	Intracellular	_	
1PSOE	Extracellular	Extracellular	Extracellular	Extracellular
1PSRA	Intracellular	Intracellular	_	
1PT6A	Intracellular	Extracellular	Extracellular	Extracellular
1PTQA	Intracellular	Intracellular	_	
1PVAA	Extracellular	Intracellular	_	
1PWAA	Intracellular	Extracellular	Extracellular	Extracellular
1PWBA	Extracellular	Extracellular	Extracellular	Extracellular

1PWTA	Intracellular	Intracellular	_	
1PYTA	Extracellular	Extracellular	Extracellular	Extracellular
1PYTC	Extracellular	Intracellular	Intracellular	Intracellular
1PYTD	Extracellular	Extracellular	Extracellular	Extracellular
1PZ5B	Extracellular	Intracellular	_	
1PZ7A	Extracellular	Extracellular	Extracellular	Extracellular
1PZ8A	Extracellular	Extracellular	Extracellular	Extracellular
1Q0EA	Intracellular	Intracellular	_	
1Q0PA	Extracellular	Extracellular	Extracellular	Extracellular
1Q0YH	Extracellular	Extracellular	_	
1Q0YH	Extracellular	Intracellular	Intracellular	Intracellular
1Q0YH	Extracellular	Extracellular	_	
1Q0YH	Intracellular	Intracellular	_	
1Q0YH	Intracellular	Intracellular	_	
1Q0YH	Intracellular	Intracellular	_	
1Q0YH	Intracellular	Intracellular	_	
1Q0YH	Intracellular	Intracellular	_	
1Q0YH	Intracellular	Intracellular	_	
1Q0YH	Intracellular	Intracellular	_	
1Q0YH	Intracellular	Intracellular	_	
1Q0YH	Intracellular	Intracellular	_	
1Q0YH	Intracellular	Intracellular	_	
1Q0YH	Intracellular	Intracellular	_	
1Q0YH	Intracellular	Intracellular	_	
1Q0YH	Extracellular	Extracellular	Extracellular	Extracellular
1Q1UA	Intracellular	Intracellular	Intracellular	Intracellular
1Q20A	Intracellular	Intracellular	_	
1Q38A	Extracellular	Extracellular	Extracellular	Extracellular
1Q3MA	Intracellular	Extracellular	Extracellular	Extracellular
1Q41A	Intracellular	Intracellular	Intracellular	Intracellular
1Q4GA	Extracellular	Intracellular	Extracellular	Extracellular
1Q56A	Extracellular	Extracellular	Extracellular	Extracellular
1Q61A	Intracellular	Intracellular	_	
1Q72H	Extracellular	Extracellular	_	
1Q72H	Extracellular	Intracellular	Intracellular	Intracellular

1Q72H	Extracellular	Extracellular	_	
1Q72H	Intracellular	Intracellular	_	
1Q72H	Intracellular	Intracellular	_	
1Q72H	Intracellular	Intracellular	_	
1Q72H	Intracellular	Intracellular	_	
1Q72H	Intracellular	Intracellular	_	
1Q72H	Intracellular	Intracellular	_	
1Q72H	Intracellular	Intracellular	_	
1Q72H	Intracellular	Intracellular	_	
1Q72H	Intracellular	Intracellular	_	
1Q72H	Intracellular	Intracellular	_	
1Q72H	Intracellular	Intracellular	_	
1Q72H	Intracellular	Intracellular	_	
1Q72H	Extracellular	Extracellular	Extracellular	Extracellular
1Q8HA	Extracellular	Extracellular	_	
1Q8LA	Intracellular	Extracellular	_	
1Q9RB	Extracellular	Intracellular	_	
1QADA	Intracellular	Intracellular	_	
1QDDA	Extracellular	Extracellular	Extracellular	Extracellular
1QDPA	Extracellular	Extracellular	_	
1QDQA	Extracellular	Extracellular	Extracellular	Extracellular
1QE6B	Intracellular	Extracellular	Extracellular	Extracellular
1QF9A	Intracellular	Intracellular	_	
1QFTA	Extracellular	Extracellular	Extracellular	Extracellular
1QG3A	Intracellular	Extracellular	Extracellular	Extracellular
1QG7A	Intracellular	Extracellular	Extracellular	Extracellular
1QHUA	Extracellular	Extracellular	Extracellular	Extracellular
1QHWA	Intracellular	Extracellular	Extracellular	Extracellular
1QK6A	Intracellular	Extracellular	Extracellular	Extracellular
1QK7A	Extracellular	Extracellular	Extracellular	Extracellular
1QKWA	Intracellular	Intracellular	_	
1QKYA	Extracellular	Extracellular	_	
1QLKA	Intracellular	Intracellular	_	
1QLLA	Extracellular	Extracellular	_	
1QLSA	Intracellular	Intracellular	_	

1QLYA	Intracellular	Intracellular	_	
1QMVA	Intracellular	Intracellular	_	
1QNXA	Extracellular	Extracellular	Extracellular	Extracellular
1QO3A	Intracellular	Intracellular	Extracellular	Intracellular
1QO3C	Intracellular	Extracellular	_	
1QO3D	Intracellular	Extracellular	_	
1QPCA	Intracellular	Intracellular	_	
1QQFA	Intracellular	Extracellular	Extracellular	Extracellular
1QQYA	Intracellular	Extracellular	_	
1QUAA	Intracellular	Intracellular	_	
1QV0A	Intracellular	Extracellular	_	
1QY1A	Intracellular	Extracellular	Extracellular	Extracellular
1QZ1A	Intracellular	Intracellular	Extracellular	Intracellular
1QZOA	Extracellular	Extracellular	_	
1QZOA	Extracellular	Intracellular	Intracellular	Intracellular
1QZOA	Extracellular	Extracellular	_	
1QZOA	Intracellular	Intracellular	_	
1QZOA	Intracellular	Intracellular	_	
1QZOA	Intracellular	Intracellular	_	
1QZOA	Intracellular	Intracellular	_	
1QZOA	Intracellular	Intracellular	_	
1QZOA	Intracellular	Intracellular	_	
1QZOA	Intracellular	Intracellular	_	
1QZOA	Intracellular	Intracellular	_	
1QZOA	Intracellular	Intracellular	_	
1QZOA	Intracellular	Intracellular	_	
1QZOA	Intracellular	Intracellular	_	
1QZOA	Intracellular	Intracellular	_	
1QZOA	Extracellular	Extracellular	Extracellular	Extracellular
1R0PA	Extracellular	Extracellular	Extracellular	Extracellular
1R0RI	Extracellular	Extracellular	_	
1R0TB	Extracellular	Extracellular	_	
1R0TB	Extracellular	Intracellular	Intracellular	Intracellular
1R0TB	Extracellular	Extracellular	_	
1R0TB	Intracellular	Intracellular	_	

1R0TB	Intracellular	Intracellular	_	
1R0TB	Intracellular	Intracellular	_	
1R0TB	Intracellular	Intracellular	_	
1R0TB	Intracellular	Intracellular	_	
1R0TB	Intracellular	Intracellular	_	
1R0TB	Intracellular	Intracellular	_	
1R0TB	Intracellular	Intracellular	_	
1R0TB	Intracellular	Intracellular	_	
1R0TB	Intracellular	Intracellular	_	
1R0TB	Intracellular	Intracellular	_	
1R0TB	Intracellular	Intracellular	_	
1R0TB	Extracellular	Extracellular	Extracellular	Extracellular
1R18A	Intracellular	Intracellular	_	
1R2QA	Intracellular	Intracellular	_	
1R3CA	Intracellular	Intracellular	_	
1R4WA	Intracellular	Intracellular	_	
1R5RA	Extracellular	Extracellular	_	
1R5RA	Extracellular	Intracellular	Intracellular	Intracellular
1R5RA	Extracellular	Extracellular	_	
1R5RA	Intracellular	Intracellular	_	
1R5RA	Intracellular	Intracellular	_	
1R5RA	Intracellular	Intracellular	_	
1R5RA	Intracellular	Intracellular	_	
1R5RA	Intracellular	Intracellular	_	
1R5RA	Intracellular	Intracellular	_	
1R5RA	Intracellular	Intracellular	_	
1R5RA	Intracellular	Intracellular	_	
1R5RA	Intracellular	Intracellular	_	
1R5RA	Intracellular	Intracellular	_	
1R5RA	Intracellular	Intracellular	_	
1R5RA	Intracellular	Intracellular	_	
1R5RA	Extracellular	Extracellular	Extracellular	Extracellular
1R6TA	Intracellular	Intracellular	_	
1R79A	Intracellular	Intracellular	_	
1R8QA	Intracellular	Intracellular	_	

1R8SA	Intracellular	Intracellular	_	
1RBDA	Intracellular	Extracellular	Extracellular	Extracellular
1RDL1	Intracellular	Extracellular	Extracellular	Extracellular
1RDQE	Intracellular	Intracellular	_	
1REOA	Intracellular	Intracellular	Extracellular	Intracellular
1REWA	Intracellular	Extracellular	Extracellular	Extracellular
1REWC	Intracellular	Intracellular	Extracellular	Intracellular
1RHFA	Intracellular	Extracellular	Extracellular	Extracellular
1RHGA	Extracellular	Extracellular	Extracellular	Extracellular
1RI9A	Intracellular	Intracellular	_	
1RIEA	Intracellular	Intracellular	Intracellular	Intracellular
1RJ8A	Intracellular	Intracellular	_	
1RJBA	Extracellular	Extracellular	Extracellular	Extracellular
1RK4A	Intracellular	Intracellular	_	
1RMIA	Extracellular	Extracellular	_	
1RMIA	Extracellular	Intracellular	Intracellular	Intracellular
1RMIA	Extracellular	Extracellular	_	
1RMIA	Intracellular	Intracellular	_	
1RMIA	Intracellular	Intracellular	_	
1RMIA	Intracellular	Intracellular	_	
1RMIA	Intracellular	Intracellular	_	
1RMIA	Intracellular	Intracellular	_	
1RMIA	Intracellular	Intracellular	_	
1RMIA	Intracellular	Intracellular	_	
1RMIA	Intracellular	Intracellular	_	
1RMIA	Intracellular	Intracellular	_	
1RMIA	Intracellular	Intracellular	_	
1RMIA	Intracellular	Intracellular	_	
1RMIA	Intracellular	Intracellular	_	
1RMIA	Extracellular	Extracellular	Extracellular	Extracellular
1RNFA	Intracellular	Extracellular	Extracellular	Extracellular
1RODA	Intracellular	Extracellular	Extracellular	Extracellular
1RP1A	Intracellular	Extracellular	Extracellular	Extracellular
1RPMA	Extracellular	Extracellular	Extracellular	Extracellular
1RPYA	Extracellular	Intracellular	_	

1RRAA	Intracellular	Extracellular	Extracellular	Extracellular
1RROA	Extracellular	Extracellular	_	
1RWYA	Intracellular	Intracellular	_	
1RYHA	Intracellular	Intracellular	_	
1RYOA	Extracellular	Extracellular	Extracellular	
1S3EA	Intracellular	Intracellular	_	
1S6BA	Extracellular	Extracellular	_	
1S6BB	Extracellular	Extracellular	Intracellular	Extracellular
1S6CA	Intracellular	Intracellular	Intracellular	Intracellular
1S6UA	Intracellular	Extracellular	_	
1S83A	Extracellular	Extracellular	_	
1S8IA	Extracellular	Extracellular	Extracellular	Extracellular
1S9VA	Extracellular	Extracellular	Extracellular	Extracellular
1S9VB	Intracellular	Intracellular	Extracellular	Intracellular
1SACA	Extracellular	Extracellular	Extracellular	Extracellular
1SCFA	Extracellular	Extracellular	Extracellular	Extracellular
1SCFC	Extracellular	Extracellular	Extracellular	Extracellular
1SCOA	Extracellular	Extracellular	_	
1SCYA	Extracellular	Extracellular	_	
1SDWA	Intracellular	Extracellular	Extracellular	Extracellular
1SEMA	Intracellular	Intracellular	_	
1SFPA	Extracellular	Extracellular	Extracellular	Extracellular
1SH1A	Extracellular	Extracellular	_	
1SHFA	Intracellular	Intracellular	_	
1SHPA	Extracellular	Extracellular	_	
1SHUX	Intracellular	Intracellular	Extracellular	Intracellular
1SISA	Extracellular	Extracellular	_	
1SKZA	Extracellular	Extracellular	Extracellular	Extracellular
1SM3H	Extracellular	Intracellular	_	
1SMDA	Extracellular	Extracellular	Extracellular	Extracellular
1SMGA	Intracellular	Intracellular	_	
1SMRA	Extracellular	Extracellular	Extracellular	Extracellular
1SN4A	Extracellular	Extracellular	_	
1SNBA	Extracellular	Extracellular	_	
1SO8A	Intracellular	Intracellular	_	

1SOXA	Intracellular	Intracellular	_	
1SPPA	Intracellular	Extracellular	Extracellular	Extracellular
1SPPB	Intracellular	Extracellular	Extracellular	Extracellular
1STFI	Intracellular	Intracellular	_	
1SVYA	Intracellular	Intracellular	_	
1SXMA	Extracellular	Extracellular	_	
1SZ8A	Extracellular	Extracellular	_	
1T2AA	Intracellular	Intracellular	_	
1T2DA	Intracellular	Extracellular	Extracellular	Extracellular
1TADA	Intracellular	Intracellular	_	
1TBGA	Intracellular	Intracellular	_	
1TBOA	Intracellular	Intracellular	_	
1TCRA	Intracellular	Extracellular	Extracellular	Extracellular
1TENA	Extracellular	Extracellular	Extracellular	Extracellular
1TETH	Intracellular	Extracellular	_	
1TFDA	Extracellular	Extracellular	Extracellular	Extracellular
1TFSA	Extracellular	Extracellular	_	
1TGJA	Intracellular	Extracellular	Extracellular	Extracellular
1TGSI	Extracellular	Extracellular	_	
1TGXA	Extracellular	Extracellular	_	
1THEA	Extracellular	Extracellular	Extracellular	Extracellular
1TITA	Intracellular		Extracellular	
1TKIA	Intracellular		Extracellular	
1TN3A	Extracellular	Extracellular	Extracellular	Extracellular
1TN4A	Intracellular	Extracellular	_	
1TONA	Extracellular	Extracellular	Extracellular	Extracellular
1TPGA	Extracellular	Extracellular	Extracellular	Extracellular
1TRNA	Extracellular	Extracellular	Extracellular	Extracellular
1TSKA	Extracellular	Extracellular	Extracellular	Extracellular
1TUCA	Intracellular	Intracellular	_	
1TVXA	Intracellular	Extracellular	Extracellular	Extracellular
1TVXB	Intracellular	Extracellular	Extracellular	Extracellular
1TXBA	Extracellular	Extracellular	_	
1TXMA	Extracellular	Extracellular	_	
1UADA	Intracellular	Intracellular	_	

1UCTA	Intracellular	Extracellular	Extracellular	Extracellular
1UDKA	Extracellular	Extracellular	_	
1UDLA	Intracellular	Intracellular	_	
1UE9A	Intracellular	Intracellular	_	
1UEMA	Intracellular	Extracellular	Extracellular	Extracellular
1UENA	Intracellular	Intracellular	Extracellular	Intracellular
1UEYA	Intracellular	Intracellular	Extracellular	Intracellular
1UFFA	Intracellular	Intracellular	_	
1UG1A	Intracellular	Intracellular	_	
1UGVA	Intracellular	Intracellular	_	
1UHCA	Intracellular	Intracellular	_	
1UJ0A	Intracellular	Intracellular	_	
1UJKA	Intracellular	Intracellular	_	
1UJTA	Intracellular	Extracellular	Extracellular	Extracellular
1UJYA	Intracellular	Intracellular	_	
1UK5A	Intracellular	Intracellular	_	
1UKMA	Extracellular	Extracellular	Extracellular	Extracellular
1UKMB	Intracellular	Extracellular	Extracellular	Extracellular
1UMRA	Extracellular	Extracellular	Extracellular	Extracellular
1UMRC	Intracellular	Extracellular	Extracellular	Extracellular
1UN3A	Intracellular	Extracellular	Extracellular	Extracellular
1UPTA	Intracellular	Extracellular	Intracellular	Intracellular
1UT3A	Intracellular	Extracellular	Intracellular	Intracellular
1UTEA	Intracellular	Extracellular	Extracellular	Extracellular
1UTIA	Intracellular	Intracellular	_	
1UU3A	Intracellular	Intracellular	_	
1UUBA	Extracellular	Extracellular	Extracellular	Extracellular
1UUHA	Extracellular	Extracellular	Extracellular	Extracellular
1UV0A	Intracellular	Extracellular	Extracellular	Extracellular
1UVQA	Extracellular	Extracellular	Extracellular	Extracellular
1UVQB	Intracellular	Extracellular	Extracellular	Extracellular
1UZPA	Extracellular	Intracellular	Extracellular	Extracellular
1V9EA	Intracellular	Intracellular	_	
1VAPA	Extracellular	Extracellular	_	
1VCAA	Intracellular	Extracellular	Extracellular	Extracellular

1VJ1A	Extracellular	Intracellular	_	
1VNAA	Extracellular	Extracellular	Extracellular	Extracellular
1VR2A	Intracellular	Extracellular	Extracellular	Extracellular
1VTXA	Extracellular	Extracellular	_	
1WDCB	Intracellular	Intracellular	_	
1WDCC	Intracellular	Intracellular	_	
1WEJF	Intracellular	Intracellular	_	
1WWBX	Intracellular	Extracellular	Extracellular	Extracellular
1WWCA	Intracellular	Intracellular	Extracellular	Intracellular
1WWWV	Intracellular	Extracellular	Extracellular	Extracellular
1WWWX	Intracellular	Intracellular	Extracellular	Intracellular
1XKBA	Extracellular	Extracellular	Extracellular	Extracellular
1XSOA	Intracellular	Intracellular	_	
1YCSB	Intracellular	Intracellular	_	
1YFOA	Intracellular	Intracellular	Extracellular	Intracellular
1ZAQA	Extracellular	Extracellular	Extracellular	Extracellular
1ZEIA	Extracellular	Extracellular	Extracellular	Extracellular
1ZFPE	Intracellular	Intracellular	_	
1ZXQA	Intracellular	Extracellular	Extracellular	Extracellular
2A2UA	Intracellular	Extracellular	Extracellular	Extracellular
2ABLA	Intracellular	Intracellular	_	
2ABXA	Extracellular	Extracellular	Extracellular	Extracellular
2AFGA	Intracellular	Intracellular	_	
2AFPA	Extracellular	Extracellular	Extracellular	Extracellular
2AK3A	Intracellular	Intracellular	Intracellular	Intracellular
2AW0A	Intracellular	Extracellular	_	
2B3CA	Extracellular	Extracellular	Extracellular	Extracellular
2BCEA	Intracellular	Intracellular	_	
2BJXA	Intracellular	Extracellular	Extracellular	Extracellular
2BMTA	Extracellular	Extracellular	Extracellular	Extracellular
2CB5A	Intracellular	Intracellular	_	
2CBLA	Intracellular	Intracellular	_	
2CDXA	Extracellular	Extracellular	Extracellular	Extracellular
2CK0H	Intracellular	Extracellular	_	
2CK0L	Intracellular	Extracellular	_	

2CSTA	Intracellular	Intracellular	_	
2CTXA	Extracellular	Extracellular	_	
2DNJA	Extracellular	Extracellular	Extracellular	Extracellular
2ECHA	Extracellular	Extracellular	_	
2EQLA	Intracellular	Extracellular	_	
2ERLA	Extracellular	Extracellular	Extracellular	Extracellular
2FBJH	Extracellular	Intracellular	_	
2FCBA	Extracellular	Extracellular	Extracellular	Extracellular
2FHEA	Intracellular	Intracellular	_	
2FN2A	Extracellular	Extracellular	Extracellular	Extracellular
2GMFA	Extracellular	Extracellular	Extracellular	Extracellular
2GSQA	Intracellular	Intracellular	_	
2GSRA	Intracellular	Intracellular	_	
2GSTA	Intracellular	Intracellular	_	
2HCCA	Extracellular	Extracellular	Extracellular	Extracellular
2HFTA	Extracellular	Intracellular	Extracellular	Extracellular
2HLCA	Intracellular	Extracellular	Extracellular	Extracellular
2IADA	Intracellular	Extracellular	Extracellular	Extracellular
2IADB	Extracellular	Extracellular	Extracellular	Extracellular
2ILAA	Intracellular	Intracellular	_	
2ILKA	Intracellular	Extracellular	Extracellular	Extracellular
2JELH	Intracellular	Extracellular	_	
2KTXA	Extracellular	Extracellular	_	
2LISA	Intracellular	Extracellular	Extracellular	Extracellular
2MFNA	Extracellular	Extracellular	Extracellular	Extracellular
2NGRA	Extracellular	Intracellular	_	
2OVOA	Extracellular	Extracellular	_	
2PCPB	Intracellular	Extracellular	_	
2PF1A	Extracellular	Extracellular	Extracellular	Extracellular
2PGDA	Intracellular	Intracellular	Extracellular	Intracellular
2PLDA	Intracellular	Intracellular	_	
2PSPA	Extracellular	Extracellular	_	
2PVBA	Extracellular	Intracellular	_	
2RELA	Extracellular	Extracellular	Extracellular	Extracellular
2RIGA	Intracellular	Extracellular	Extracellular	Extracellular

2SASA	Intracellular	Intracellular	_	
2SCPA	Intracellular	Intracellular	_	
2SHPA	Intracellular	Intracellular	_	
2SN3A	Extracellular	Extracellular	Extracellular	Extracellular
2TGIA	Intracellular	Extracellular	Extracellular	Extracellular
2TMPA	Extracellular	Extracellular	Extracellular	Extracellular
2TNFA	Extracellular	Extracellular	_	
2VIKA	Intracellular	Intracellular	_	
3ADKA	Intracellular	Intracellular	_	
3CMSA	Extracellular	Extracellular	Extracellular	Extracellular
3EBXA	Extracellular	Extracellular	Extracellular	Extracellular
3ERKA	Intracellular	Intracellular	_	
3FRUA	Intracellular	Extracellular	Extracellular	Extracellular
3GRSA	Intracellular	Intracellular	Intracellular	Intracellular
3IL8A	Intracellular	Extracellular	Extracellular	Extracellular
3KIVA	Extracellular	Extracellular	Extracellular	Extracellular
3LRIA	Intracellular	Extracellular	Extracellular	Extracellular
3LYNA	Intracellular	Intracellular	Extracellular	Intracellular
3LZTA	Extracellular	Extracellular	Extracellular	Extracellular
3OVOA	Extracellular	Extracellular	_	
3PBHA	Extracellular	Extracellular	Extracellular	Extracellular
3RABA	Extracellular	Extracellular	_	
3RP2A	Intracellular	Extracellular	Extracellular	Extracellular
4AIGA	Intracellular	Intracellular	Intracellular	Intracellular
4HTCI	Extracellular	Extracellular	_	
4MDHA	Intracellular	Intracellular	Extracellular	Intracellular
4PEPA	Extracellular	Extracellular	Extracellular	Extracellular
4TSVA	Intracellular	Extracellular	_	
5CYTR	Intracellular	Intracellular	_	
5HPGA	Extracellular	Extracellular	Extracellular	Extracellular
5P2PA	Extracellular	Extracellular	Extracellular	Extracellular
5PALA	Intracellular	Intracellular	_	
7AATA	Intracellular	Intracellular	Intracellular	Intracellular
8I1BA	Intracellular	Intracellular	_	
9LDTA	Intracellular	Intracellular	_	

1ABRB	Intracellular	Intracellular	_	
1AC5A	Intracellular	Intracellular	Intracellular	Intracellular
1AE1A	Intracellular	Intracellular	_	
1AECA	Extracellular	Intracellular	Extracellular	Extracellular
1AG6A	Intracellular	Intracellular	Intracellular	Intracellular
1AKYA	Intracellular	Intracellular	_	
1AOZA	Extracellular	Intracellular	_	
1APAA	Extracellular	Intracellular	Extracellular	Extracellular
1APXA	Intracellular	Intracellular	_	
1AQ0A	Extracellular	Intracellular	_	
1AQZA	Intracellular	Intracellular	Extracellular	Intracellular
1ARUA	Intracellular	Extracellular	Extracellular	Extracellular
1AUNA	Extracellular	Intracellular	Extracellular	Intracellular
1AVAA	Intracellular	Extracellular	Extracellular	Extracellular
1AVAC	Extracellular	Intracellular	Intracellular	Intracellular
1AVBA	Intracellular	Intracellular	Extracellular	Intracellular
1AVWB	Intracellular	Extracellular	Extracellular	Extracellular
1AXDA	Intracellular	Intracellular	_	
1AYJA	Extracellular	Extracellular	Extracellular	Extracellular
1AYXA	Extracellular	Intracellular	Extracellular	Extracellular
1AZ6A	Extracellular	Intracellular	Intracellular	Intracellular
1AZJA	Extracellular	Intracellular	Intracellular	Intracellular
1AZKA	Extracellular	Intracellular	Intracellular	Intracellular
1B37A	Intracellular	Intracellular	Extracellular	Intracellular
1B80A	Intracellular	Intracellular	Extracellular	Intracellular
1BEAA	Extracellular	Intracellular	Extracellular	Extracellular
1BG4A	Extracellular	Extracellular	_	
1BHPA	Extracellular	Extracellular	Extracellular	Extracellular
1BK1A	Extracellular	Intracellular	Extracellular	Extracellular
1BOLA	Intracellular	Intracellular	Extracellular	Intracellular
1BRYY	Intracellular	Extracellular	Extracellular	Extracellular
1BRZA	Extracellular	Intracellular	_	
1BWOA	Extracellular	Extracellular	Extracellular	Extracellular
1BXOA	Extracellular	Intracellular	_	
1BYPA	Intracellular	Intracellular	Intracellular	Intracellular

1C2AA	Extracellular	Intracellular	_	
1C6RA	Extracellular	Intracellular	_	
1CBGA	Intracellular	Intracellular	_	
1CC8A	Intracellular	Extracellular	_	
1CCRA	Intracellular	Intracellular	_	
1CEXA	Extracellular	Intracellular	Intracellular	Intracellular
1CF3A	Extracellular	Intracellular	Extracellular	Extracellular
1CFEA	Extracellular	Extracellular	Extracellular	Extracellular
1CLVI	Extracellular	Intracellular	_	
1CNSA	Extracellular	Extracellular	Extracellular	Extracellular
1CNVA	Intracellular	Extracellular	Extracellular	Extracellular
1COAI	Intracellular	Intracellular	_	
1CQDA	Extracellular	Intracellular	_	
1CRLA	Intracellular	Intracellular	Extracellular	Intracellular
1CSNA	Intracellular	Intracellular	_	
1CTJA	Extracellular	Intracellular	_	
1CYJA	Extracellular	Intracellular	Intracellular	Intracellular
1CZFA	Intracellular	Extracellular	Extracellular	Extracellular
1D2KA	Intracellular	Intracellular	Extracellular	Intracellular
1D6AA	Extracellular	Intracellular	Extracellular	Extracellular
1D6RI	Extracellular	Intracellular	Extracellular	Extracellular
1DBYA	Intracellular	Intracellular	Intracellular	Intracellular
1DE3A	Intracellular	Extracellular	Extracellular	Extracellular
1DF6A	Extracellular	Intracellular	_	
1DF9C	Extracellular	Intracellular	Intracellular	Intracellular
1DGWA	Intracellular	Extracellular	Extracellular	Extracellular
1DHKB	Extracellular	Intracellular	Extracellular	Extracellular
1DIXA	Extracellular	Extracellular	Extracellular	Extracellular
1DKCA	Extracellular	Extracellular	Extracellular	Extracellular
1DL2A	Intracellular	Intracellular	Extracellular	Intracellular
1DPJA	Intracellular	Extracellular	Extracellular	Extracellular
1DU5A	Extracellular	Intracellular	Extracellular	Extracellular
1DWMA	Extracellular	Intracellular	Intracellular	Intracellular
1DYSA	Intracellular	Intracellular	_	
1E4MM	Intracellular	Extracellular	_	

1E6BA	Intracellular	Intracellular	Intracellular	Intracellular
1EAGA	Intracellular	Intracellular	Extracellular	Intracellular
1EB6A	Intracellular	Intracellular	Extracellular	Intracellular
1EDOA	Intracellular	Intracellular	Intracellular	Intracellular
1EJ8A	Intracellular	Extracellular	_	
1EJGA	Extracellular	Intracellular	_	
1EK0A	Intracellular	Intracellular	Extracellular	Intracellular
1EKMA	Intracellular	Intracellular	Intracellular	Intracellular
1EN2A	Extracellular	Intracellular	Extracellular	Intracellular
1ENPA	Intracellular	Intracellular	Intracellular	Intracellular
1EP7A	Intracellular	Intracellular	_	
1EQCA	Extracellular	Intracellular	Extracellular	Extracellular
1EQKA	Intracellular	Intracellular	Extracellular	Intracellular
1ERJA	Extracellular	Intracellular	_	
1ERJA	Extracellular	Intracellular	Intracellular	Intracellular
1ERJA	Extracellular	Intracellular	_	
1ERJA	Intracellular	Intracellular	_	
1ERJA	Intracellular	Intracellular	_	
1ERJA	Intracellular	Intracellular	_	
1ERJA	Intracellular	Intracellular	_	
1ERJA	Intracellular	Intracellular	_	
1ERJA	Intracellular	Intracellular	_	
1ERJA	Intracellular	Intracellular	_	
1ERJA	Intracellular	Intracellular	_	
1ERJA	Intracellular	Intracellular	_	
1ERJA	Intracellular	Intracellular	_	
1ERJA	Intracellular	Intracellular	_	
1ERJA	Intracellular	Intracellular	_	
1ERJA	Extracellular	Extracellular	Extracellular	Extracellular
1EYLA	Intracellular	Intracellular	Extracellular	Intracellular
1EZVE	Intracellular	Intracellular	Intracellular	Intracellular
1F2SI	Intracellular		_	
1F54A	Intracellular	Intracellular	_	
1F5MA	Intracellular	Intracellular	_	
1F60A	Intracellular	Intracellular	_	

1F9MA	Intracellular	Intracellular	Intracellular	Intracellular
1FB6A	Intracellular	Intracellular	Intracellular	Intracellular
1FI2A	Intracellular	Intracellular	_	
1FK5A	Extracellular	Intracellular	Extracellular	Extracellular
1FNCA	Intracellular	Intracellular	Intracellular	Intracellular
1FNYA	Extracellular	Intracellular	Extracellular	Extracellular
1FOBA	Extracellular	Intracellular	Extracellular	Extracellular
1FPWA	Intracellular	Intracellular	_	
1FUSA	Extracellular	Intracellular	Extracellular	Extracellular
1FVQA	Intracellular	Extracellular	_	
1FX5A	Intracellular	Intracellular	_	
1G0OA	Intracellular	Intracellular	_	
1G12A	Extracellular	Extracellular	Extracellular	Extracellular
1G16A	Intracellular	Intracellular	_	
1G66A	Intracellular	Intracellular	Extracellular	Intracellular
1G7YA	Extracellular	Intracellular	Extracellular	Extracellular
1G9FA	Extracellular	Intracellular	Extracellular	Extracellular
1GAIA	Extracellular	Intracellular	Extracellular	Extracellular
1GDNA	Extracellular	Intracellular	Extracellular	Extracellular
1GDVA	Intracellular	Extracellular	Extracellular	Extracellular
1GECE	Extracellular	Extracellular	Extracellular	Extracellular
1GGWA	Intracellular	Intracellular	_	
1GHSA	Extracellular	Intracellular	Extracellular	Extracellular
1GK8A	Intracellular	Intracellular	_	
1GKYA	Intracellular	Intracellular	_	
1GNWA	Intracellular	Intracellular	_	
1GPEA	Intracellular	Intracellular	_	
1GPSA	Extracellular	Intracellular	Intracellular	Intracellular
1GPTA	Extracellular	Intracellular	Intracellular	
1GQ8A	Extracellular	Intracellular	_	
1GU7A	Intracellular	Intracellular	Intracellular	Intracellular
1GW0A	Extracellular	Intracellular	Extracellular	Extracellular
1GWUA	Intracellular	Extracellular	Extracellular	Extracellular
1GYCA	Extracellular	Intracellular	Extracellular	Extracellular
1GZ7A	Extracellular	Intracellular	Extracellular	Extracellular

1GZCA	Extracellular	Intracellular	Extracellular	Extracellular
1H20A	Extracellular	Intracellular	_	
1H34A	Intracellular		Intracellular	
1H49A	Intracellular	Intracellular	Intracellular	Intracellular
1H4PA	Intracellular	Intracellular	Extracellular	Intracellular
1H5QA	Intracellular	Intracellular	Extracellular	Intracellular
1H65A	Intracellular	Intracellular	_	
1H9WA	Extracellular	Extracellular	_	
1HG8A	Intracellular	Intracellular	Intracellular	Intracellular
1HJSA	Extracellular	Intracellular	_	
1HSSA	Extracellular	Intracellular	Intracellular	Intracellular
1HYPA	Intracellular	Extracellular	Extracellular	Extracellular
1I0VA	Extracellular	Intracellular	Extracellular	Extracellular
1I1WA	Extracellular	Intracellular	Extracellular	Extracellular
1I24A	Intracellular	Intracellular	Intracellular	Intracellular
1IA5A	Intracellular	Extracellular	Intracellular	Intracellular
1IB9A	Extracellular	Intracellular	_	
1IBQA	Extracellular	Intracellular	Intracellular	Intracellular
1IDKA	Extracellular	Extracellular	Extracellular	Extracellular
1IHPA	Extracellular	Intracellular	Extracellular	Extracellular
1IOOA	Intracellular	Intracellular	Extracellular	Intracellular
1IQQA	Extracellular	Intracellular	Extracellular	Extracellular
1IUZA	Intracellular	Intracellular	_	
1J1QA	Intracellular	Intracellular	_	
1J71A	Intracellular	Extracellular	Extracellular	Extracellular
1JCVA	Intracellular	Intracellular	_	
1JDRA	Intracellular	Intracellular	Intracellular	Intracellular
1JEHA	Intracellular	Intracellular	Intracellular	Intracellular
1JERA	Intracellular	Intracellular	_	
1JJZA	Extracellular	Extracellular	Extracellular	Extracellular
1JKZA	Extracellular	Intracellular	_	
1JMNA	Extracellular	Extracellular	_	
1JMPA	Extracellular	Intracellular	Intracellular	Intracellular
1JO8A	Intracellular	Intracellular	_	
1JPCA	Extracellular	Extracellular	Extracellular	Extracellular

1JR8A	Intracellular	Intracellular	Extracellular	Intracellular
1JXCA	Extracellular	Extracellular	Extracellular	Extracellular
1K0DA	Intracellular	Intracellular	Intracellular	Intracellular
1K0DD	Intracellular	Intracellular	Intracellular	Intracellular
1K3IA	Extracellular	Intracellular	Extracellular	Extracellular
1KALA	Extracellular	Extracellular	Extracellular	Extracellular
1KBIA	Intracellular	Intracellular	Intracellular	Intracellular
1KDGA	Extracellular	Intracellular	Extracellular	Extracellular
1KDJA	Intracellular	Intracellular	_	
1KHIA	Extracellular	Intracellular	_	
1KHQA	Extracellular	Extracellular	Extracellular	Extracellular
1KREA	Extracellular	Intracellular	Extracellular	Extracellular
1KSIA	Intracellular	Intracellular	Extracellular	Intracellular
1KULA	Extracellular	Intracellular	Extracellular	Extracellular
1KY3A	Intracellular	Intracellular	_	
1L6HA	Extracellular	Extracellular	Extracellular	Extracellular
1LEDA	Extracellular	Intracellular	_	
1LGYA	Extracellular	Intracellular	Intracellular	Intracellular
1LJPA	Extracellular	Intracellular	Intracellular	Intracellular
1LK9A	Extracellular	Extracellular	Extracellular	Extracellular
1LKJA	Intracellular	Intracellular	_	
1LLFA	Intracellular	Intracellular	Extracellular	Intracellular
1LLNA	Intracellular	Extracellular	Extracellular	Extracellular
1LLPA	Intracellular	Extracellular	Extracellular	Extracellular
1LMSA	Intracellular	Intracellular	_	
1LP8A	Intracellular	Extracellular	Extracellular	Extracellular
1LRHA	Intracellular	Intracellular	Extracellular	Intracellular
1LRIA	Extracellular	Intracellular	Extracellular	Extracellular
1LS9A	Extracellular	Intracellular	_	
1LU0A	Extracellular		_	
1LW6I	Intracellular	Intracellular	_	
1M2OB	Intracellular	Intracellular	Extracellular	Intracellular
1M2RA	Intracellular	Intracellular	_	
1M2TA	Extracellular	Intracellular	Intracellular	Intracellular
1M2TB	Extracellular	Intracellular	Intracellular	Intracellular

1M45A	Intracellular	Intracellular	_	
1MCTI	Extracellular		_	
1MIDA	Extracellular	Extracellular	Extracellular	Extracellular
1MN2A	Extracellular	Intracellular	Extracellular	Extracellular
1MPPA	Intracellular	Intracellular	Extracellular	Intracellular
1MR3F	Intracellular	Intracellular	_	
1MR4A	Extracellular	Extracellular	Extracellular	Extracellular
1MRGA	Intracellular	Intracellular	Extracellular	Intracellular
1MRJA	Extracellular	Intracellular	Extracellular	Extracellular
1MVQA	Intracellular	Extracellular	_	
1MVZA	Extracellular	Intracellular	Intracellular	Intracellular
1N0UA	Intracellular	Intracellular	_	
1N7HA	Intracellular	Intracellular	_	
1N89A	Extracellular	Extracellular	Extracellular	Extracellular
1NB1A	Extracellular	Extracellular	Extracellular	Extracellular
1NBJA	Extracellular	Intracellular	_	
1NBLA	Extracellular	Intracellular	Intracellular	Intracellular
1NH2A	Intracellular	Intracellular	_	
1NHCA	Intracellular	Extracellular	Intracellular	Intracellular
1NIOA	Intracellular	Intracellular	Intracellular	Intracellular
1NLSA	Extracellular	Extracellular	Extracellular	
1NM7A	Intracellular	Intracellular	Intracellular	Intracellular
1NRJB	Intracellular	Intracellular	Extracellular	
1O0EA	Extracellular	Intracellular	_	
1OC7A	Extracellular	Extracellular	Extracellular	Extracellular
1OD5A	Intracellular	Intracellular	Extracellular	Intracellular
1OEWA	Extracellular	Intracellular	Extracellular	Intracellular
1OJJA	Extracellular	Intracellular	_	
1OKHA	Extracellular	Intracellular	Extracellular	Intracellular
1OM0A	Intracellular	Intracellular	Extracellular	Intracellular
1ONKA	Extracellular	Intracellular	Intracellular	Intracellular
1ONKB	Extracellular	Intracellular	Intracellular	Intracellular
1OOTA	Intracellular	Intracellular	_	
1OQPA	Intracellular	Intracellular	_	
1ORLA	Extracellular	Intracellular	_	

1OYJA	Intracellular	Intracellular	_	
1OYVI	Extracellular	Extracellular	Extracellular	Extracellular
1P8BA	Extracellular	Intracellular	Extracellular	Extracellular
1PA2A	Intracellular	Intracellular	Extracellular	Intracellular
1PCVA	Extracellular	Extracellular	Extracellular	Extracellular
1PGUA	Intracellular	Intracellular	_	
1PI2A	Extracellular	Intracellular	_	
1PJUA	Extracellular	Extracellular	Extracellular	Extracellular
1PLBA	Intracellular	Intracellular	_	
1PLCA	Intracellular	Intracellular	Intracellular	Intracellular
1PPOA	Extracellular	Intracellular	Extracellular	
1PVXA	Extracellular	Intracellular	_	
1Q2BA	Extracellular	Intracellular	Intracellular	Intracellular
1Q44A	Intracellular	Intracellular	_	
1Q4MA	Extracellular	Intracellular	_	
1Q4MA	Extracellular	Intracellular	Intracellular	Intracellular
1Q4MA	Extracellular	Intracellular	_	
1Q4MA	Intracellular	Intracellular	_	
1Q4MA	Intracellular	Intracellular	_	
1Q4MA	Intracellular	Intracellular	_	
1Q4MA	Intracellular	Intracellular	_	
1Q4MA	Intracellular	Intracellular	_	
1Q4MA	Intracellular	Intracellular	_	
1Q4MA	Intracellular	Intracellular	_	
1Q4MA	Intracellular	Intracellular	_	
1Q4MA	Intracellular	Intracellular	_	
1Q4MA	Intracellular	Intracellular	_	
1Q4MA	Intracellular	Intracellular	_	
1Q4MA	Intracellular	Intracellular	_	
1Q4MA	Extracellular	Extracellular	Extracellular	Extracellular
1Q8YA	Intracellular	Intracellular	_	
1Q9BA	Extracellular	Extracellular	Extracellular	Extracellular
1QCXA	Extracellular	Intracellular	Extracellular	Extracellular
1QDMA	Extracellular	Intracellular	Extracellular	Extracellular
1QFXA	Extracellular	Intracellular	Extracellular	Extracellular

1QFZA	Intracellular	Intracellular	Intracellular	Intracellular
1QGJA	Intracellular	Intracellular	Extracellular	Intracellular
1QI7A	Intracellular	Extracellular	Extracellular	Extracellular
1QI9A	Intracellular	Intracellular	_	
1QJWA	Extracellular	Intracellular	_	
1QMGA	Intracellular	Intracellular	Intracellular	Intracellular
1QNAA	Intracellular	Intracellular	_	
1QOZA	Extracellular	Extracellular	_	
1QPAA	Extracellular	Extracellular	Extracellular	Extracellular
1QUPA	Intracellular	Extracellular	_	
1R4YA	Intracellular	Extracellular	Extracellular	Extracellular
1R64A	Intracellular	Intracellular	Intracellular	Intracellular
1RFSA	Intracellular	Intracellular	Intracellular	Intracellular
1RMGA	Extracellular	Intracellular	Intracellular	Intracellular
1RQWA	Extracellular	Intracellular	_	
1RTUA	Extracellular	Intracellular	_	
1RZLA	Extracellular	Extracellular	Extracellular	Extracellular
1S4VA	Intracellular	Intracellular	Extracellular	Intracellular
1SRDA	Extracellular	Intracellular	Intracellular	Intracellular
1TABI	Intracellular	Intracellular	Intracellular	Intracellular
1TCAA	Extracellular	Intracellular	Extracellular	Extracellular
1THGA	Extracellular	Intracellular	Extracellular	Extracellular
1TIAA	Extracellular	Intracellular	Extracellular	Extracellular
1TIBA	Extracellular	Intracellular	Extracellular	Extracellular
1TIEA	Intracellular	Intracellular	_	
1TMKA	Intracellular	Intracellular	_	
1TMQB	Extracellular	Intracellular	Intracellular	Intracellular
1TUXA	Extracellular	Intracellular	Extracellular	Extracellular
1UASA	Extracellular	Intracellular	Extracellular	Extracellular
1UCAA	Intracellular	Intracellular	Intracellular	Intracellular
1UHAA	Extracellular	Intracellular	_	
1UHNA	Extracellular	Extracellular	Extracellular	Extracellular
1UKZA	Intracellular	Intracellular	_	
1ULKA	Extracellular	Intracellular	Extracellular	Extracellular
1UQ5A	Extracellular	Extracellular	Extracellular	Extracellular

1UWCA	Extracellular	Extracellular	Extracellular	Extracellular
1V6IA	Extracellular	Extracellular	Extracellular	Extracellular
1VDCA	Intracellular	Intracellular	_	
1VK0A	Intracellular	Intracellular	_	
1WBAA	Intracellular	Intracellular	_	
1WBFA	Extracellular	Intracellular	_	
1WGTA	Extracellular	Intracellular	_	
1XNDA	Extracellular	Intracellular	_	
1XYNA	Extracellular	Intracellular	Extracellular	Extracellular
1XYOA	Extracellular	Intracellular	Intracellular	Intracellular
1YAAA	Intracellular	Intracellular	_	
1YALA	Extracellular	Intracellular	Extracellular	Extracellular
1YCCA	Intracellular	Intracellular	_	
1YEBA	Intracellular	Intracellular	_	
1YNAA	Extracellular	Intracellular	Extracellular	Extracellular
1YPCI	Intracellular	Intracellular	_	
1YTCA	Intracellular	Intracellular	_	
2AAAA	Extracellular	Intracellular	Intracellular	Intracellular
2AAIB	Extracellular	Extracellular	Extracellular	Extracellular
2ACTA	Extracellular	Intracellular	Extracellular	Extracellular
2AE1A	Intracellular	Intracellular	_	
2APRA	Intracellular	Extracellular	Extracellular	Extracellular
2ASIA	Extracellular	Intracellular	Intracellular	Intracellular
2BBIA	Extracellular	Intracellular	Extracellular	Extracellular
2BTCI	Extracellular	Intracellular	_	
2CBPA	Extracellular	Intracellular	_	
2CNAA	Extracellular	Extracellular	Extracellular	Extracellular
2CNDA	Intracellular	Intracellular	_	
2ENGA	Extracellular	Intracellular	_	
2HVMA	Intracellular	Intracellular	Extracellular	Intracellular
2LETA	Extracellular	Intracellular	_	
2PHLA	Intracellular	Extracellular	Extracellular	Extracellular
2PLHA	Extracellular	Intracellular	Intracellular	Intracellular
2PLTA	Extracellular	Intracellular	Intracellular	Intracellular
3GCBA	Intracellular	Intracellular	Intracellular	Intracellular

3OVWA	Intracellular	Extracellular	Extracellular	Extracellular
3RUBL	Intracellular	Intracellular	_	
3TGLA	Extracellular	Intracellular	Extracellular	Extracellular
4CPAI	Extracellular	Intracellular	_	
4SGBI	Extracellular	Intracellular	Extracellular	Extracellular
7MDHA	Intracellular	Intracellular	Intracellular	Intracellular
7PCYA	Intracellular	Intracellular	_	
7TAAA	Extracellular	Intracellular	Extracellular	Extracellular
7WGAA	Extracellular	Intracellular	Extracellular	Extracellular
8RUCA	Intracellular	Intracellular	_	
9PCYA	Intracellular	Intracellular	_	
9WGAA	Extracellular	Intracellular	Extracellular	Extracellular
155CA	Extracellular			
1A2PA	Extracellular			
1A5ZA	Intracellular			
1A62A	Intracellular			
1A7TA	Intracellular			
1A8HA	Intracellular			
1A8PA	Intracellular			
1A99A	Extracellular			
1AA6A	Intracellular			
1AACA	Extracellular			
1ACXA	Extracellular			
1ADWA	Extracellular			
1AFJA	Extracellular			
1AGJA	Intracellular			
1AH9A	Intracellular			
1AISA	Intracellular			
1AIWA	Extracellular			
1AJKA	Extracellular			
1AJOA	Extracellular			
1AKOA	Intracellular			
1AKPA	Extracellular			
1AL3A	Intracellular			
1AMFA	Extracellular			

1AN8A	Intracellular
1ANUA	Extracellular
1AOHA	Extracellular
1ARBA	Extracellular
1AYGA	Extracellular
1AZCA	Extracellular
1B5PA	Intracellular
1B8PA	Intracellular
1BAGA	Extracellular
1BDBA	Intracellular
1BEDA	Extracellular
1BF2A	Extracellular
1BG6A	Intracellular
1BH6A	Extracellular
1BHEA	Extracellular
1BKBA	Intracellular
1BLUA	Intracellular
1BN8A	Extracellular
1BQBA	Extracellular
1BQKA	Extracellular
1BXKA	Intracellular
1BXUA	Extracellular
1BYKA	Intracellular
1C2RA	Extracellular
1C3CA	Intracellular
1C4QA	Intracellular
1C53A	Extracellular
1C6SA	Extracellular
1C75A	Intracellular
1C9OA	Intracellular
1CC3A	Extracellular
1CC5A	Extracellular
1CEOA	Intracellular
1CG2A	Intracellular
1CGTA	Extracellular

1CHKA	Extracellular
1CIUA	Extracellular
1CIYA	Intracellular
1CKEA	Intracellular
1CLCA	Extracellular
1CLFA	Extracellular
1CLXA	Extracellular
1CNOA	Extracellular
1CO6A	Extracellular
1CORA	Extracellular
1COTA	Extracellular
1COYA	Extracellular
1CPMA	Extracellular
1CPNA	Extracellular
1CPZA	Intracellular
1CQXA	Intracellular
1CSPA	Intracellular
1CUOA	Extracellular
1CV8A	Extracellular
1CVLA	Extracellular
1CWVA	Extracellular
1CX1A	Extracellular
1CXCA	Extracellular
1CXYA	Intracellular
1CYGA	Extracellular
1D1HA	Extracellular
1D5AA	Intracellular
1DARA	Intracellular
1DB3A	Intracellular
1DBIA	Extracellular
1DBQA	Intracellular
1DJRD	Intracellular
1DKIA	Extracellular
1DKLA	Extracellular
1DL5A	Intracellular

1DLCA	Extracellular
1DLJA	Intracellular
1DMSA	Intracellular
1DPEA	Extracellular
1DQZA	Extracellular
1DRKA	Extracellular
1DURA	Extracellular
1DVHA	Extracellular
1DVVA	Extracellular
1DW0A	Extracellular
1DYPA	Extracellular
1DYZA	Extracellular
1DZOA	Extracellular
1E29A	Intracellular
1E30A	Extracellular
1E3PA	Intracellular
1E43A	Extracellular
1E4EA	Intracellular
1E4IA	Intracellular
1E4YA	Intracellular
1E5CA	Extracellular
1E6UA	Intracellular
1ECEA	Extracellular
1ED7A	Extracellular
1ED8A	Extracellular
1EDGA	Extracellular
1EDTA	Extracellular
1EFCA	Intracellular
1EFUB	Intracellular
1EGAA	Intracellular
1EGOA	Intracellular
1EGZA	Extracellular
1EKEA	Intracellular
1EL5A	Intracellular
1ELJA	Extracellular

1ENFA	Intracellular
1ENYA	Intracellular
1EOKA	Extracellular
1EP3B	Intracellular
1EPWA	Intracellular
1EQ2A	Intracellular
1EQWA	Extracellular
1ESOA	Extracellular
1ET9A	Intracellular
1EU1A	Intracellular
1EURA	Extracellular
1EUTA	Extracellular
1EXHA	Extracellular
1EXMA	Intracellular
1EZ4A	Intracellular
1EZMA	Extracellular
1EZSA	Extracellular
1F00I	Extracellular
1F0NA	Extracellular
1F1CA	Extracellular
1F1SA	Extracellular
1F83A	Intracellular
1F83A	Intracellular
1F83A	Intracellular
1F83A	Intracellular
1F83A	Intracellular
1F83A	Intracellular
1F83A	Intracellular
1F83A	Intracellular
1F83A	Intracellular
1F83A	Intracellular
1F83A	Intracellular
1F83A	Intracellular
1F83A	Intracellular
1F83A	Intracellular

1F83A	Intracellular
1F83A	Intracellular
1F8EA	Extracellular
1FA4A	Extracellular
1FCAA	Intracellular
1FDRA	Intracellular
1FFYA	Intracellular
1FG7A	Intracellular
1FI3A	Extracellular
1FJJA	Intracellular
1FL2A	Intracellular
1FMCA	Intracellular
1FNOA	Intracellular
1FNUA	Intracellular
1FO5A	Intracellular
1FOVA	Intracellular
1FQTA	Intracellular
1FUQA	Intracellular
1FUXA	Extracellular
1FVKA	Extracellular
1FXDA	Intracellular
1FXJA	Intracellular
1FXRA	Intracellular
1FXXA	Intracellular
1G0CA	Intracellular
1G24A	Extracellular
1G4US	Extracellular
1G5AA	Intracellular
1G6PA	Extracellular
1G72A	Extracellular
1G72B	Extracellular
1G7OA	Intracellular
1G7SA	Intracellular
1G87A	Extracellular
1G8KA	Extracellular

1G8KB	Extracellular
1G94A	Extracellular
1G97A	Intracellular
1G9GA	Extracellular
1GA6A	Extracellular
1GBGA	Extracellular
1GCAA	Extracellular
1GCIA	Extracellular
1GCXA	Intracellular
1GCXA	Intracellular
1GCXA	Intracellular
1GCXA	Intracellular
1GCXA	Intracellular
1GCXA	Intracellular
1GCXA	Intracellular
1GCXA	Intracellular
1GCXA	Intracellular
1GCXA	Intracellular
1GCXA	Intracellular
1GCXA	Intracellular
1GCXA	Intracellular
1GCXA	Intracellular
1GCXA	Intracellular
1GCXA	Intracellular
1GCYA	Extracellular
1GEEA	Intracellular
1GEGA	Intracellular
1GERA	Intracellular
1GKSA	Extracellular
1GNSA	Extracellular
1GO3E	Intracellular
1GOUA	Extracellular
1GPQA	Extracellular
1GSIA	Intracellular
1GSKA	Intracellular

1GTRA	Intracellular
1GU2A	Extracellular
1GU3A	Extracellular
1GUDA	Extracellular
1GV0A	Intracellular
1GV1A	Intracellular
1GV1A	Intracellular
1GVHA	Intracellular
1H0HA	Extracellular
1H3FA	Intracellular
1H4IA	Extracellular
1H4IB	Extracellular
1H75A	Intracellular
1HDCA	Intracellular
1HDHA	Intracellular
1HEJC	Extracellular
1HIZA	Extracellular
1HPGA	Extracellular
1HPWA	Extracellular
1HROA	Extracellular
1HSLA	Extracellular
1HV9A	Intracellular
1HVXA	Extracellular
1HXHA	Intracellular
1HYEA	Intracellular
1HYHA	Intracellular
1HYUA	Intracellular
1I39A	Intracellular
1I4GA	Intracellular
1I4PA	Intracellular
1I5PA	Extracellular
1I6AA	Intracellular
1I6LA	Intracellular
1I8OA	Extracellular
1IC6A	Extracellular

1ID2A	Extracellular
1II5A	Intracellular
1IKPA	Intracellular
1ILEA	Intracellular
1IO2A	Intracellular
1IOWA	Intracellular
1IQ0A	Intracellular
1IQZA	Intracellular
1ITXA	Extracellular
1IVWA	Intracellular
1IXCA	Intracellular
1IXHA	Extracellular
1IY8A	Intracellular
1IZ6A	Intracellular
1IZ9A	Intracellular
1J09A	Intracellular
1J0HA	Intracellular
1J18A	Extracellular
1J1UA	Intracellular
1J33A	Intracellular
1J3GA	Intracellular
1J3VA	Intracellular
1J3VA	Intracellular
1J3VA	Intracellular
1J3VA	Intracellular
1J3VA	Intracellular
1J3VA	Intracellular
1J3VA	Intracellular
1J3VA	Intracellular
1J3VA	Intracellular
1J3VA	Intracellular
1J3VA	Intracellular
1J3VA	Intracellular
1J3VA	Intracellular
1J3VA	Intracellular

1J3VA	Intracellular
1J3VA	Intracellular
1J48A	Extracellular
1J54A	Intracellular
1J58A	Intracellular
1J5VA	Intracellular
1J5VA	Intracellular
1J5VA	Intracellular
1J5VA	Intracellular
1J5VA	Intracellular
1J5VA	Intracellular
1J5VA	Intracellular
1J5VA	Intracellular
1J5VA	Intracellular
1J5VA	Intracellular
1J5VA	Intracellular
1J5VA	Intracellular
1J5VA	Intracellular
1J5VA	Intracellular
1J5VA	Intracellular
1J5VA	Intracellular
1J7NA	Intracellular
1J9AA	Intracellular
1J9QA	Extracellular
1JAKA	Intracellular
1JALA	Intracellular
1JB0C	Extracellular
1JDLA	Extracellular
1JETA	Extracellular
1JFUA	Extracellular
1JFXA	Extracellular
1JG1A	Intracellular
1JHFA	Intracellular
1JI1A	Extracellular
1JI2A	Intracellular

1JI6A	Intracellular
1JIWI	Extracellular
1JJ2A	Extracellular
1JJEA	Intracellular
1JJVA	Intracellular
1JL1A	Intracellular
1JNYA	Intracellular
1JQBA	Intracellular
1JT8A	Intracellular
1JU3A	Intracellular
1JVBA	Intracellular
1JW7A	Intracellular
1JW7A	Intracellular
1JW7A	Intracellular
1JW7A	Intracellular
1JW7A	Intracellular
1JW7A	Intracellular
1JW7A	Intracellular
1JW7A	Intracellular
1JW7A	Intracellular
1JW7A	Intracellular
1JW7A	Intracellular
1JW7A	Intracellular
1JW7A	Intracellular
1JW7A	Intracellular
1JW7A	Intracellular
1JW7A	Intracellular
1JX6A	Intracellular
1JYEA	Intracellular
1JZ7A	Intracellular
1JZGA	Extracellular
1K0IA	Intracellular
1K0RA	Intracellular
1K0VA	Intracellular
1K2WA	Intracellular

1K85A	Extracellular
1K8WA	Intracellular
1KB0A	Extracellular
1KBVA	Extracellular
1KBZA	Extracellular
1KCLA	Extracellular
1KEWA	Intracellular
1KFSA	Intracellular
1KHTA	Extracellular
1KITA	Extracellular
1KJOA	Extracellular
1KK1A	Intracellular
1KLUD	Intracellular
1KLXA	Extracellular
1KNGA	Extracellular
1KNMA	Extracellular
1KOLA	Intracellular
1KOPA	Extracellular
1KOQA	Extracellular
1KQFA	Extracellular
1KQKA	Intracellular
1KRHA	Intracellular
1KS9A	Intracellular
1KV7A	Extracellular
1KWFA	Extracellular
1KYGA	Intracellular
1KYGA	Intracellular
1KYGA	Intracellular
1KYGA	Intracellular
1KYGA	Intracellular
1KYGA	Intracellular
1KYGA	Intracellular
1KYGA	Intracellular
1KYGA	Intracellular
1KYGA	Intracellular

1KYGA	Intracellular
1KYGA	Intracellular
1KYGA	Intracellular
1KYGA	Intracellular
1KYGA	Intracellular
1KYGA	Intracellular
1L1YA	Extracellular
1L4IA	Extracellular
1L5OA	Intracellular
1L6RA	Intracellular
1LC5A	Intracellular
1LDNA	Intracellular
1LI5A	Intracellular
1LLDA	Intracellular
1LNIA	Intracellular
1LNSA	Intracellular
1LQRA	Intracellular
1LQRA	Intracellular
1LQRA	Intracellular
1LQRA	Intracellular
1LQRA	Intracellular
1LQRA	Intracellular
1LQRA	Intracellular
1LQRA	Intracellular
1LQRA	Intracellular
1LQRA	Intracellular
1LQRA	Intracellular
1LQRA	Intracellular
1LQRA	Intracellular
1LQRA	Intracellular
1LQRA	Intracellular
1LQRA	Intracellular
1LR0A	Extracellular
1LRWA	Extracellular
1LRWB	Extracellular

1LSTA	Extracellular
1LU4A	Extracellular
1LUPA	Extracellular
1LVLA	Intracellular
1LYVA	Extracellular
1M2XA	Extracellular
1M7XA	Intracellular
1MDAH	Intracellular
1MDAH	Intracellular
1MDAH	Intracellular
1MDAH	Intracellular
1MDAH	Intracellular
1MDAH	Intracellular
1MDAH	Intracellular
1MDAH	Intracellular
1MDAH	Intracellular
1MDAH	Intracellular
1MDAH	Intracellular
1MDAH	Intracellular
1MDAH	Intracellular
1MDAH	Intracellular
1MDAH	Intracellular
1MDAH	Intracellular
1MDAL	Intracellular
1MDAL	Intracellular
1MDAL	Intracellular
1MDAL	Intracellular
1MDAL	Intracellular
1MDAL	Intracellular
1MDAL	Intracellular
1MDAL	Intracellular
1MDAL	Intracellular
1MDAL	Intracellular
1MDAL	Intracellular
1MDAL	Intracellular

1MDAL	Intracellular
1MDAL	Intracellular
1MDAL	Intracellular
1MDAL	Intracellular
1MEEA	Extracellular
1MGRA	Intracellular
1MJCA	Intracellular
1MJNA	Intracellular
1MKYA	Intracellular
1MO9A	Intracellular
1MOYA	Extracellular
1MP9A	Intracellular
1MTZA	Intracellular
1MV8A	Intracellular
1MVEA	Extracellular
1MWYA	Intracellular
1MZ4A	Intracellular
1N02A	Extracellular
1N12A	Extracellular
1N2AA	Intracellular
1N2ZA	Extracellular
1N4WA	Extracellular
1N8JA	Intracellular
1NBCA	Extracellular
1NE3A	Intracellular
1NFFA	Intracellular
1NG4A	Intracellular
1NH8A	Intracellular
1NHOA	Intracellular
1NHPA	Intracellular
1NIFA	Extracellular
1NNFA	Extracellular
1NNSA	Extracellular
1NOAA	Extracellular
1NP3A	Intracellular

1NPCA	Extracellular
1NRHX	Intracellular
1NRHX	Intracellular
1NRHX	Intracellular
1NRHX	Intracellular
1NRHX	Intracellular
1NRHX	Intracellular
1NRHX	Intracellular
1NRHX	Intracellular
1NRHX	Intracellular
1NRHX	Intracellular
1NRHX	Intracellular
1NRHX	Intracellular
1NRHX	Intracellular
1NRHX	Intracellular
1NRHX	Intracellular
1NRHX	Intracellular
1NT4A	Extracellular
1NW2A	Extracellular
1NWPA	Extracellular
1NY4A	Intracellular
1NYAA	Intracellular
1NYCA	Intracellular
1NZJA	Intracellular
1O4SA	Intracellular
1O63A	Intracellular
1O6YA	Intracellular
1O6ZA	Intracellular
1O7JA	Intracellular
1O88A	Extracellular
1O89A	Intracellular
1OACA	Extracellular
1OALA	Extracellular
1OAPA	Extracellular
1OBBA	Intracellular

1OD8A	Extracellular
1ODZA	Extracellular
1OGIA	Extracellular
1OIMA	Intracellular
1OLLA	Intracellular
1ON4A	Intracellular
1OQ3A	Intracellular
1OQSA	Intracellular
1OQSB	Extracellular
1OQWA	Extracellular
1ORRA	Intracellular
1OS8A	Extracellular
1OUVA	Extracellular
1P3JA	Intracellular
1P4SA	Intracellular
1P5VA	Extracellular
1P5VB	Extracellular
1PAMA	Extracellular
1PAZA	Extracellular
1PBGA	Intracellular
1PCLA	Extracellular
1PCSA	Extracellular
1PDAA	Intracellular
1PDKB	Intracellular
1PE9A	Extracellular
1PFVA	Intracellular
1PGSA	Extracellular
1PMTA	Intracellular
1PMYA	Extracellular
1POTA	Extracellular
1PSQA	Intracellular
1PSZA	Extracellular
1PUIA	Extracellular
1PXVA	Extracellular
1Q16A	Intracellular

1Q1RA	Intracellular
1Q5NA	Intracellular
1Q7CA	Intracellular
1Q8IA	Intracellular
1Q98A	Intracellular
1QB5D	Extracellular
1QBAA	Extracellular
1QE3A	Intracellular
1QFJA	Intracellular
1QGIA	Extracellular
1QHOA	Extracellular
1QHQA	Extracellular
1QHTA	Intracellular
1QISA	Intracellular
1QJVA	Extracellular
1QL3A	Extracellular
1QO0A	Intracellular
1QORA	Intracellular
1QPXA	Extracellular
1QQ9A	Extracellular
1QSGA	Intracellular
1QTFA	Extracellular
1QTMA	Intracellular
1QTRA	Intracellular
1QW9A	Intracellular
1QWDA	Intracellular
1QXHA	Intracellular
1QY6A	Extracellular
1R0RE	Extracellular
1R1MA	Extracellular
1R3EA	Intracellular
1R4PA	Intracellular
1R88A	Extracellular
1R9LA	Extracellular
1RBLA	Intracellular

1RFLA	Intracellular
1RIPA	Intracellular
1RJWA	Intracellular
1RKDA	Intracellular
1RKQA	Intracellular
1RKRA	Extracellular
1RL2A	Extracellular
1RMSA	Extracellular
1RPNA	Intracellular
1RTQA	Extracellular
1RXJA	Extracellular
1S01A	Extracellular
1S0UA	Intracellular
1S3GA	Intracellular
1S4QA	Intracellular
1S5DA	Extracellular
1S96A	Intracellular
1SBPA	Extracellular
1SGPE	Extracellular
1SGVA	Intracellular
1SLUA	Extracellular
1SMLA	Extracellular
1SMPI	Extracellular
1SPVA	Intracellular
1SROA	Intracellular
1SSXA	Extracellular
1ST9A	Intracellular
1SUPA	Extracellular
1SWGA	Extracellular
1TF4A	Extracellular
1TFEA	Intracellular
1TGOA	Intracellular
1THMA	Extracellular
1THTA	Intracellular
1THXA	Intracellular

1TMLA	Extracellular
1TMOA	Intracellular
1TOAA	Intracellular
1TRBA	Intracellular
1UC7A	Intracellular
1UDBA	Intracellular
1UHVA	Intracellular
1UIUA	Extracellular
1UMUA	Intracellular
1UN2A	Extracellular
1UOKA	Intracellular
1UR5A	Intracellular
1USGA	Extracellular
1UU1A	Intracellular
1UUFA	Intracellular
1UUZA	Extracellular
1VHMA	Intracellular
1VHTA	Intracellular
1VHUA	Intracellular
1VIXA	Intracellular
1VJWA	Intracellular
1WDNA	Extracellular
1WSAA	Intracellular
1XNBA	Extracellular
1XYZA	Extracellular
1ZINA	Intracellular
2APSA	Extracellular
2AY1A	Intracellular
2B3IA	Extracellular
2BATA	Extracellular
2BBKL	Extracellular
2BC2A	Intracellular
2CMDA	Intracellular
2EBNA	Extracellular
2EIFA	Extracellular

2FDNA	Intracellular
2FNBA	Extracellular
2GBPA	Extracellular
2LIVA	Extracellular
2MADH	Intracellular
2MCMA	Extracellular
2MTAC	Intracellular
2NAPA	Intracellular
2PIAA	Extracellular
2RBIA	Extracellular
2SFAA	Extracellular
2SGAA	Extracellular
2TRXA	Extracellular
2TS1A	Intracellular
3C2CA	Extracellular
3CHBD	Intracellular
3EZMA	Extracellular
3LADA	Intracellular
3MBPA	Extracellular
3SEBA	Intracellular
3SILA	Extracellular
3THIA	Extracellular
3TSSA	Extracellular
451CA	Extracellular
4AAHB	Extracellular
4LIPD	Extracellular
4PGAA	Extracellular
4TMKA	Intracellular
5RUBA	Intracellular
7A3HA	Extracellular
8ABPA	Extracellular
