# Supplementary material for: Analysis on conservation of disulphide bonds and their structural features in homologous protein domain families
Source: BMC Struct Biol. 2008 Dec 26;8:55. doi: 10.1186/1472-6807-8-55 (PMC2628669; doi:10.1186/1472-6807-8-55)
Supplement: Additional file 7 — Literature survey of proteins highly predicted to be cytoplasmic by all three programs. This table provides the results of literature survey on comments of cellular localization for the protein chains, that are predicted to be cytoplasmic, by all three programs or by any two out of three programs. [file 1472-6807-8-55-S7.pdf]

Supplementary Table 2: Literature survey of proteins highly predicted to be cytoplasmic by all three programs				
1A4KH	DIELS ALDER CATALYTIC ANTIBODY WITH TRANSITION STATE ANALOGUE	Extracellular	Antibodies occur in two forms: a soluble form secreted into the blood and other fluids in the body, and a membrane-bound form that is attached to the surface of a B cell.	
1AOGA	TRYPANOSOMA CRUZI TRYPANOTHIONE REDUCTASE (OXIDIZED FORM)	cytoplasm	GO annotation	
1AQWA	GLUTATHIONE S-TRANSFERASE IN COMPLEX WITH GLUTATHIONE	cytoplasm	GO annotation	
1AXIB	STRUCTURAL PLASTICITY AT THE HGH:HGHBP INTERFACE	Extracellular	GO annotation	
1B2TA	SOLUTION STRUCTURE OF THE CX3C CHEMOKINE DOMAIN OF FRACTALKINE	Extracellular	GO annotation	
1CEJA	SOLUTION STRUCTURE OF AN EGF MODULE PAIR FROM THE PLASMODIUM FALCIPARUM MEROZOITE SURFACE PROTEIN 1		GO annotation	
1CFBA	CRYSTAL STRUCTURE OF TANDEM TYPE III FIBRONECTIN DOMAINS FROM DROSOPHILA NEUROGLIAN AT 2.0 ANGSTROMS	Extracellular	GO annotation	
1CNT1	CILIARY NEUROTROPHIC FACTOR	Extracellular	CNTF itself lacks a classical signal peptide sequence of a secreted protein, but is thought to convey its cytoprotective effects after release from adult glial cells by some mechanism induced by injury.	The ciliary neurotrophic factor and its receptor, CNTFR alpha. Sleeman MW, Anderson KD, Lambert PD, Yancopoulos GD, Wiegand SJ. Regeneron Pharmaceuticals, Tarrytown, NY 10591-6707, USA. mark.sleeman@regpha.com PMID: 10812968
1CYDA	CARBONYL REDUCTASE COMPLEXED WITH NADPH AND 2-PROPANOL	Mitochondria	GO annotation	
1D2EA	CRYSTAL STRUCTURE OF MITOCHONDRIAL EF-TU IN COMPLEX WITH GDP	Mitochondria	GO annotation	
1D4XG	Crystal Structure of Caenorhabditis Elegans Mg-ATP Actin Complexed with Human Gelsolin Segment 1 at 1.75 A resolution.	Both Extracellular and cytoplasm	GO annotation	
1E6WA	RAT BRAIN 3-HYDROXYACYL-COA DEHYDROGENASE BINARY COMPLEX WITH NADH AND ESTRADIOL	cytoplasm	GO annotation	
1E7WA	ONE ACTIVE SITE, TWO MODES OF REDUCTION CORRELATE THE MECHANISM OF LEISHMANIA PTERIDINE REDUCTASE WITH PTERIN METABOLISM AND ANTIFOLATE DRUG RESISTANCE IN TRPANOSOMES	Extracellular	Biological evaluation of selected inhibitors was performed against the Extracellular forms of T. cruzi and L. major, both wild-type and overexpressing PTR1 lines, as a model for PTR1-driven antifolate drug resistance and the intracellular form of T. cruzi.	Discovery of potent pteridine reductase inhibitors to guide antiparasite drug development. Proceedings of the National Academy of Sciences of the United States of America 2008;105(5):1448-53.
1EDHA	E-CADHERIN DOMAINS 1 AND 2 IN COMPLEX WITH CALCIUM	Extracellular	GO annotation	

1EM2A	STAR-RELATED LIPID TRANSPORT DOMAIN OF MLN64	Extracellular	GO annotation	
1EPFA	CRYSTAL STRUCTURE OF THE TWO N-TERMINAL IMMUNOGLOBULIN DOMAINS OF THE NEURAL CELL ADHESION MOLECULE (NCAM)	Extracellular	GO annotation	
1EW2A	CRYSTAL STRUCTURE OF A HUMAN PHOSPHATASE	Extracellular	GO annotation	
1F14A	L-3-HYDROXYACYL-COA DEHYDROGENASE (APO)	cytoplasm	GO annotation	
1F2LA	CRYSTAL STRUCTURE OF CHEMOKINE DOMAIN OF FRACTALKINE	Extracellular	GO annotation	
1F6BA	CRYSTAL STRUCTURE OF CHEMOKINE DOMAIN OF FRACTALKINE	cytoplasm	GO annotation	
1F8RA	CRYSTAL STRUCTURE OF L-AMINO ACID OXIDASE FROM CALLOSELASMA RHODOSTOMA COMPLEXED WITH CITRATE	Extracellular	GO annotation	
1F8ZA	NMR STRUCTURE OF THE SIXTH LIGAND-BINDING MODULE OF THE LDL RECEPTOR	Extracellular	GO annotation	
1FIDA	STRUCTURE OF HUMAN GAMMA FIBRINOGEN 30 KD CARBOXYL TERMINAL FRAGMENT	Extracellular	GO annotation	
1FKNA	STRUCTURE OF BETA-SECRETASE COMPLEXED WITH INHIBITOR	Both Extracellular and cytoplasm	GO annotation	
1FLTX	VEGF IN COMPLEX WITH DOMAIN 2 OF THE FLT-1 RECEPTOR	Extracellular	GO annotation	
1FNGA	HISTOCOMPATIBILITY ANTIGEN	Extracellular	GO annotation	
1FONA	CRYSTAL STRUCTURE OF BOVINE PROCARBOXYPEPTIDASE A-S6 SUBUNIT III, A HIGHLY STRUCTURED TRUNCATED ZYMOGEN E	Extracellular	GO annotation	
1FP0A	HSC20 (HSCB), A J-TYPE CO-CHAPERONE FROM E. COLI	**Both Extracellular and cytoplasm**	Although many co-chaperones are soluble cytosolic proteins, co-chaperone domains are also found in modular adaptor proteins, which are often localized to intracellular membranes or elements of the cytoskeleton.	More than folding: localized functions of cytosolic chaperonesYOUNG Jason C. ; BARRAL José M. ; HARTL F. Ulrich ;Trends in biochemical sciences 2003, vol. 28, no10, pp.
1FSUA	4-SULFATASE (HUMAN)	cytoplasm	GO annotation	
1FW1A	GLUTATHIONE TRANSFERASE ZETA/MALEYLACETOACETATE ISOMERASE	cytoplasm	GO annotation	
1GMZA	CRYSTAL STRUCTURE OF THE D49 PHOSPHOLIPASE A2 PIRATOXIN III FROM BOTHROPS PIRAJAI.	Extracellular	GO annotation	
1GP1A	THE REFINED STRUCTURE OF THE SELENOENZYME GLUTATHIONE PEROXIDASE AT 0.2-NM RESOLUTION	cytoplasm	GO annotation	
1GV4A	MURINE APOPTOSIS-INDUCING FACTOR (AIF)	cytoplasm	GO annotation	

1GXRA	ALPHA-,1,3 GALACTOSYLTRANSFERASE- N-ACETYL LACTOSAMINE COMPLEX	Both Extracellular and cytoplasm	GO annotation	
1GZ6A	(3R)-HYDROXYACYL-COA DEHYDROGENASE FRAGMENT OF RAT PEROXISOMAL MULTIFUNCTIONAL ENZYME TYPE 2	cytoplasm	GO annotation	
1H3UA	CRYSTAL STRUCTURE OF THE HUMAN IGG1 FC-FRAGMENT,GLYCOFORM (M3N2F)2	Extracellular	GO annotation	
1HD2A	HUMAN PEROXIREDOXIN 5	cytoplasm	GO annotation	
1HDRA	THE CRYSTALLOGRAPHIC STRUCTURE OF A HUMAN DIHYDROPTERIDINE REDUCTASE NADH BINARY COMPLEX EXPRESSED IN ESCHERICHIA COLI BY A CDNA CONSTRUCTED FROM ITS RAT HOMOLOGUE	cytoplasm	GO annotation	
1HNFA	CRYSTAL STRUCTURE OF THE Extracellular REGION OF THE HUMAN CELL ADHESION MOLECULE CD2 AT 2.5 ANGSTROMS RESOLUTION	Extracellular	GO annotation	
1I1IP	NEUROLYSIN (ENDOPEPTIDASE 24.16) CRYSTAL STRUCTURE	Cytoplasm	GO annotation	
1I6ZA	BAG DOMAIN OF BAG1 COCHAPERONE	Both Extracellular and cytoplasm	Although many co-chaperones are soluble cytosolic proteins, co-chaperone domains are also found in modular adaptor proteins, which are often localized to intracellular membranes or elements of the cytoskeleton.	More than folding: localized functions of cytosolic chaperonesYOUNG Jason C. ; BARRAL José M. ; HARTL F. Ulrich ;Trends in biochemical sciences 2003, vol. 28, no10, pp.
1I7PA	CRYSTAL STRUCTURE OF RAT B5R IN COMPLEX WITH FAD	Both Extracellular and cytoplasm	GO annotation	
1IILE	SUBSTRATE BOUND PHOSPHOSERINE PHOSPHATASE COMPLEX STRUCTURE	Extracellular	GO annotation	
1JLIA	HUMAN INTERLEUKIN 3 (IL-3) MUTANT WITH TRUNCATION AT BOTH N-AND C-TERMINI AND 14 RESIDUE CHANGES, NMR, MINIMIZED AVERAGE STRUCTURE	Extracellular	GO annotation	
1JTVA	Crystal structure of 17beta-Hydroxysteroid Dehydrogenase Type 1 complexed with Testosterone	cytoplasm	GO annotation	
1JWOA	Crystal Structure Analysis of the SH2 Domain of the Csk Homologous Kinase CHK	cytoplasm	GO annotation	
1K8DA	crystal structure of the non-classical MHC class Ib Qa-2 complexed with a self peptide	Extracellular	GO annotation	
1KEQA	Crystal Structure of F65A/Y131C Carbonic Anhydrase V, covalently modified with 4-chloromethylimidazole	cytoplasm	GO annotation	
1KEXA	Crystal Structure of the b1 Domain of Human Neuropilin-1	Extracellular	GO annotation	
1KJSA	NMR SOLUTION STRUCTURE OF C5A AT PH 5.2, 303K, 20 STRUCTURES	Extracellular	GO annotation	

1L6XA	FC FRAGMENT OF RITUXIMAB BOUND TO A MINIMIZED VERSION OF THE B-DOMAIN FROM PROTEIN A CALLED Z34C	Extracellular	GO annotation	
1LCYA	Crystal Structure of the Mitochondrial Serine Protease HtrA2	Both Extracellular and cytoplasm	GO annotation	
1LVGA	Crystal structure of mouse guanylate kinase in complex with GMP and ADP			
1LWRA	Solution structure of the NCAM fibronectin type III module 2	Extracellular	GO annotation	
1LXIA	Refinement of BMP7 crystal structure	Extracellular	GO annotation	
1M39A	Solution structure of the C-terminal fragment (F86-I165) of the human centrin 2 in calcium saturated form	cytoplasm	GO annotation	
1M6IA	Crystal Structure of Apoptosis Inducing Factor (AIF)	Cytoplasm,Mitochondrial membrane	GO annotation	
1M9ZA	CRYSTAL STRUCTURE OF HUMAN TGF-BETA TYPE II RECEPTOR LIGAND BINDING DOMAIN	Extracellular	GO annotation	
1MH5B	The Structure Of The Complex Of The Fab Fragment Of The Esterolytic Antibody MS6-164 and A Transition-State Analog	Extracellular	Antibodies occur in two forms: a soluble form secreted into the blood and other fluids in the body, and a membrane-bound form that is attached to the surface of a B cell.	
1MHQA	Crystal Structure Of Human GGA2 VHS Domain	Both Extracellular and cytoplasm	GO annotation	
1MJ4A	Crystal Structure Analysis of the cytochrome b5 domain of human sulfite oxidase	cytosol	GO annotation	
1MLDA	REFINED STRUCTURE OF MITOCHONDRIAL MALATE DEHYDROGENASE FROM PORCINE HEART AND THE CONSENSUS STRUCTURE FOR DICARBOXYLIC ACID OXIDOREDUCTASES	Mitochondria	GO annotation	
1MM0A	Solution structure of termicin, an antimicrobial peptide from the termite Pseudacanthotermes spiniger	Extracellular	GO annotation	
1N5DA	CRYSTAL STRUCTURE OF PORCINE TESTICULAR CARBONYL REDUCTASE/ 20BETA-HYDROXYSTEROID DEHYDROGENASE	Cytoplasm	GO annotation	
1NCIA	STRUCTURAL BASIS OF CELL-CELL ADHESION BY CADHERINS	Extracellular	GO annotation	
1NN5A	Crystal structure of human thymidylate kinase with d4TMP + AppNHp	Cytoplasm	GO annotation	
1NOWA	Human lysosomal beta-hexosaminidase isoform B in complex with (2R,3R,4S,5R)-2-Acetamido-3,4-Dihydroxy-5-Hydroxymethyl-Piperidinium Chloride (GalNAc-isofagomine)	Both Extracellular and cytoplasm	GO annotation	

1NSTA	THE SULFOTRANSFERASE DOMAIN OF HUMAN HAPARIN SULFATE N-DEACETYLASE/N-SULFOTRANSFERASE	Both Extracellular and cytoplasm	GO annotation	
1O6SB	INTERNALIN (LISTERIA MONOCYTOGENES) / E-CADHERIN (HUMAN) RECOGNITION COMPLEX	Both Extracellular and cytoplasm	GO annotation	
1OP4A	Solution Structure of Neural Cadherin Prodomain	Extracellular	GO annotation	
1OZNA	1.5A Crystal Structure of the Nogo Receptor Ligand Binding Domain Reveals a Convergent Recognition Scaffold Mediating Inhibition of Myelination	Both Extracellular and cytoplasm	GO annotation	
1P15A	Crystal structure of the D2 domain of RPTPa	Extracellular	GO annotation	
1P8XA	The Calcium-Activated C-terminal half of gelsolin	Both Extracellular and cytoplasm	GO annotation	
1PLOA	TRANSFORMING GROWTH FACTOR-BETA TYPE II RECEPTOR Extracellular DOMAIN	Extracellular	GO annotation	
1PR9A	Human L-Xylulose Reductase Holoenzyme	Extracellular	GO annotation	
1PYTC	TERNARY COMPLEX OF PROCARBOXYPEPTIDASE A, PROPROTEINASE E, AND CHYMOTRYPSINOGEN C	Extracellular	GO annotation	
1Q0YH	Anti-Morphine Antibody 9B1 Complexed with Morphine	Extracellular	Antibodies occur in two forms: a soluble form secreted into the blood and other fluids in the body, and a membrane-bound form that is attached to the surface of a B cell.	
1Q1UA	Crystal structure of human FHF1b (FGF12b)	Both Extracellular and cytoplasm	GO annotation	
1Q41A	GSK-3 Beta complexed with Indirubin-3'-monoxime	cytoplam	GO annotation	
1Q72H	Anti-Cocaine Antibody M82G2 Complexed with Cocaine	Extracellular	Antibodies occur in two forms: a soluble form secreted into the blood and other fluids in the body, and a membrane-bound form that is attached to the surface of a B cell.	
1QO3A	COMPLEX BETWEEN NK CELL RECEPTOR LY49A AND ITS MHC CLASS I LIGAND H-2DD	Extracellular	GO annotation	
1QZ1A	Crystal Structure of the Ig 1-2-3 fragment of NCAM	Extracellular	GO annotation	
1QZOA	Three dimensional structure of a goat signalling protein secreted during involution	Extracellular	GO annotation	
1R0TB	Crystal Structure of Trypsin- Ovomucoid turkey egg white inhibitor complex			

1R5RA	Soft-SAD crystal structure of a pheromone binding protein from the honeybee Apis mellifera L.	Extracellular	Odorant binding<br>proteins are (1) small, water soluble, and Extracellular proteins<br>that (2) are located in the fluid surrounding the sensory dendrite and (3) bind odorants (Vogt and Riddiford, 198 lb;<br>Pelosi et al., 1982; Pevsner et al., 1985).	Expression of Pheromone Binding Proteins During Antenna1<br>Development in the Gypsy Moth Lymantria dispar<br>Ft. G. Vogt, A. C. Kiihne," J. T. Dubnau, and G. D. Prestwich,The Journal of Neuroscience, September 1989, g(9): 3332-3346
1REOA	L-amino acid oxidase from Agkistrodon halys pallas	Extracellular	GO annotation	
1REWC	Structural refinement of the complex of bone morphogenetic protein 2 and its type IA receptor	Extracellular	GO annotation	
1RIEA	STRUCTURE OF A WATER SOLUBLE FRAGMENT OF THE RIESKE IRON-SULFUR PROTEIN OF THE BOVINE HEART MITOCHONDRIAL CYTOCHROME BC1-	Both Extracellular and cytoplasm	GO annotation	
1RMIA	Crystal structure of recombinant murine interferon beta	Extracellular	GO annotation	
1S6CA	Crystal structure of the complex between KChIP1 and Kv4.2 N1-30	Extracellular	GO annotation	
1S9VB	Crystal structure of HLA-DQ2 complexed with deamidated gliadin peptide	Extracellular	GO annotation	
1SHUX	Crystal Structure of the von Willebrand factor A domain of human capillary morphogenesis protein 2: an anthrax toxin receptor	Extracellular	GO annotation	
1UENA	Solution Structure of The Third Fibronectin III Domain of Human KIAA0343 Protein	Extracellular	GO annotation	
1UEYA	Solution Structure of The First Fibronectin Type III Domain of Human KIAA0343 protein	Extracellular	GO annotation	
1UPTA	TRUCTURE OF A COMPLEX OF THE GOLGIN-245 GRIP DOMAIN WITH ARL1	Both Extracellular and cytoplasm	GO annotation	
1UT3A	SOLUTION STRUCTURE OF SPHENISCIN-2, A BETA-DEFENSIN FROM PENGUIN STOMACH PRESERVING FOOD	Extracellular	GO annotation	
1WWCA	NT3 BINDING DOMAIN OF HUMAN TRKC RECEPTOR	Extracellular	GO annotation	
1WWWX	NGF IN COMPLEX WITH DOMAIN 5 OF THE TRKA RECEPTOR	Both Extracellular and cytoplasm	GO annotation	
1YFOA	RECEPTOR PROTEIN TYROSINE PHOSPHATASE ALPHA, DOMAIN 1 FROM MOUSE	Extracellular	GO annotation	
2AK3A	THE THREE-DIMENSIONAL STRUCTURE OF THE COMPLEX BETWEEN MITOCHONDRIAL MATRIX ADENYLATE KINASE AND ITS SUBSTRATE AMP AT 1.85 ANGSTROMS	Cytoplasm	GO annotation	

2PGDA	THE STRUCTURE OF 6-PHOSPHOGLUCONATE DEHYDROGENASE REFINED AT 2 ANGSTROMS RESOLUTION	Cytoplasm	Adams, M.J., Gover, S., Leaback, R., Phillips, C., Somers, D.O. (1991)	
			he structure of 6-phosphogluconate dehydrogenase refined at 2.5 A resolution.	
			Acta Crystallogr., Sect.B 47: 817-820	
3GRSA	REFINED STRUCTURE OF GLUTATHIONE REDUCTASE AT 1.54 ANGSTROMS RESOLUTION	Cytoplasm	GO annotation	
3LYNA	STRUCTURE OF GREEN ABALONE LYSIN DIMER	Extracellular	Abalone sperm lysin is a non-enzymatic protein that creates a hole for sperm passage in the envelope surrounding the egg. Lysin exhibits species-specificity in making the hole and it evolves rapidly by positive selection. Our studies have focused on combining structural, biochemical, and evolutionary data to understand the mechanism of action and evolution of this remarkable protein.AP, acrosomal process	
4AIGA	ADAMALYSIN II WITH PHOSPHONATE INHIBITOR	Extracellular	Reprolysins, also known as adamalysins [1. W. Bode, F.X. Gomis-Rüth and W. Stöcker. FEBS Lett. 331 (1993), pp. 134–140. Abstract | PDF (790 K) | View Record in Scopus | Cited By in Scopus (295)1], are snake venom zinc-endopeptidases that hydrolyze basement membrane proteins involved in the adhesion among capillary endothelial cells, inducing extensive haemorrhages in the bitten preys [2]. Matrixins (matrix metalloproteinases, MMPs) are zinc-dependent enzymes that degrade the major components of the Extracellular matrix	
4MDHA	REFINED CRYSTAL STRUCTURE OF CYTOPLASMIC MALATE DEHYDROGENASE AT 2.5-ANGSTROMS RESOLUTION	Cytoplasm	GO annotation	
7AATA	X-RAY STRUCTURE REFINEMENT AND COMPARISON OF THREE FORMS OF MITOCHONDRIAL ASPARTATE AMINOTRANSFERASE	Both Extracellular and cytoplasm	GO annotation	
1AC5A	CRYSTAL STRUCTURE OF KEX1(DELTA)P, A PROHORMONE-PROCESSING CARBOXYPEPTIDASE FROM SACCHAROMYCES CEREVISIAE	Both Extracellular and cytoplasm	GO annotation	
1AG6A	PLASTOCYANIN FROM SPINACH	Both Extracellular and cytoplasm	GO annotation	
1AQZA	CRYSTAL STRUCTURE OF A HIGHLY SPECIFIC ASPERGILLUS RIBOTOXIN, RESTRICTOCIN	Extracellular	GO annotation	

1AUNA	PATHOGENESIS-RELATED PROTEIN 5D FROM NICOTIANA TABACUM	Extracellular	Four PR-5 protein isoforms (PR-5a to PR-5d) isolated from tobacco tissues accumulate differently in the Extracellular space or vacuole of plant cells [Van Loon 1976, Singh et al 1987 and Koiwa et al 1994. H. Koiwa, F. Sato and Y. Yamada, Characterization of accumulation of PR-5 proteins by IEF-immunoblot analysis. Plant Cell Physiol. 35 (1994), pp. 821–827. View Record in Scopus l Cited By in Scopus (28)Koiwa et al 1994]. Several seed proteins also have been classified in the PR-5 family [Vigers et al 1990]	
1AVAC	AMY2/BASI PROTEIN-PROTEIN COMPLEX FROM BARLEY SEED			
1AVBA	ARCELIN-1 FROM PHASEOLUS VULGARIS L	Extracellular	There are a>number of ways by which vacuolar lectins can interact with molecules within and outside the cell. First, when dry seeds imbibe water, vacuolar proteins and especially lectins are released into the imbibition water (Fountain et al., 1977). This results in the presence of lectins in the vicinity of the germinating seed, where they can interact with potential pathogens. Second, when seeds<br>or other plant organs are eaten by predators, lectins will be released from the disrupted cellular structures of the plant tissues. These lectins will then come in contact with<br>the glycoproteins that line the intestinal tracts of the predators,<br>possibly inhibiting absorption of nutrients. Third,<br>when funga1 hyphae grow into plant tissues, they may disrupt cellular compartmentation, causing the release of	
1AZ6A	THREE-DIMENSIONAL STRUCTURES OF THREE ENGINEERED CELLULOSE-BINDING DOMAINS OF CELLOBIOHYDROLASE I FROM TRICHODERMA REESEI, NMR, 23 STRUCTURES	Extracellular	GO annotation	
1AZJA	THREE-DIMENSIONAL STRUCTURES OF THREE ENGINEERED CELLULOSE-BINDING DOMAINS OF CELLOBIOHYDROLASE I FROM TRICHODERMA REESEI, NMR, 18 STRUCTURES	Extracellular	GO annotation	
1AZKA	THREE-DIMENSIONAL STRUCTURES OF THREE ENGINEERED CELLULOSE-BINDING DOMAINS OF CELLOBIOHYDROLASE I FROM TRICHODERMA REESEI, NMR, 19 STRUCTURES	Extracellular	GO annotation	

1B37A	A 30 ANGSTROM U-SHAPED CATALYTIC TUNNEL IN THE CRYSTAL STRUCTURE OF POLYAMINE OXIDASE	Extracellular	etried to isolate microorganism which could produce Extracellular polyamine oxidase and found a fungus which produced the enzyme in the mediumcontaining polyamine as a sole source of nitrogen.	Short Communication Production of Extracellular Polyamine Oxidase by Penicillium sp. No. PO-1 Yoshinori Kobayashi* and Koki HorikoshiAgric. Biol. Chem., 45 (12), 2943-2945, 1981
1B80A	REC. LIGNIN PEROXIDASE H8 OXIDATIVELY PROCESSED	Both Extracellular and cytoplasm	Intra- and Extracellular Localization of Lignin Peroxidase during the Degradation of Solid Wood and Wood Fragments by Phanerochaete chrysosporium by Using Transmission Electron Microscopy and Immuno-Gold Labeling Geoffrey Daniel,1* Thomas Nilsson,1 and Bert Pettersson2Appl Environ Microbiol. 1989 April; 55(4): 871–881	The enzyme was localized in the peripheral regions of the fungal cell cytoplasm in association with the cell membrane, fungal cell wall, and Extracellular slime materials.
1BOLA	THE CRYSTAL STRUCTURE OF RIBONUCLEASE RH FROM RHIZOPUS NIVEUS AT 2.0 A RESOLUTION			
1BYPA	E43K,D44K DOUBLE MUTANT PLASTOCYANIN FROM SILENE	**Both Extracellular and cytoplasm**	GO annotation	
1CEXA	STRUCTURE OF CUTINASE	Extracellular	GO annotation	
1CRLA	INSIGHTS INTO INTERFACIAL ACTIVATION FROM AN 'OPEN' STRUCTURE OF CANDIDA RUGOSA LIPASE			
1CYJA	CYTOCHROME C6	Cytoplasm	GO annotation	
1D2KA	C. IMMITIS CHITINASE 1 AT 2.2 ANGSTROMS RESOLUTION	Extracellular	Two chitinase genes (CTS1 and CTS2) of C. immitis have been cloned. Preliminary evidence has suggested that expression of CTS1 is markedly increased during endospore formation. The secreted CTS1 chitinase has also been shown to react with patient anti-Coccidioides complement-fixing (CF) antibody and is a valuable aid in the serodiagnosis of coccidioidomycosis.	Disruption of the Gene Which Encodes a Serodiagnostic Antigen and Chitinase of the Human Fungal Pathogen Coccidioides immitis Utz Reichard, Chiung-Yu Hung, Pei W. Thomas, and Garry T. Cole* Infection and Immunity, October 2000, p. 5830-5838, Vol. 68, No. 10
1DBYA	NMR STRUCTURES OF CHLOROPLAST THIOREDOXIN M CH2 FROM THE GREEN ALGA CHLAMYDOMONAS REINHARDTII	Chloroplast	GO annotation	
1DF9C	DENGUE VIRUS NS3-PROTEASE COMPLEXED WITH MUNG-BEAN BOWMAN-BIRK INHIBITOR	Extracellular	GO annotation	
1DL2A	CRYSTAL STRUCTURE OF CLASS I ALPHA-1,2-MANNOSIDASE FROM SACCHAROMYCES CEREVISIAE AT 1.54 ANGSTROM RESOLUTION	Both Extracellular and cytoplasm	GO annotation	
1DWMA	SOLUTION STRUCTURE OF LINUM USITATISSINUM TRYPSIN INHIBITOR (LUTI)			
1E6BA	CRYSTAL STRUCTURE OF A ZETA CLASS GLUTATHIONE S-TRANSFERASE FROM ARABIDOPSIS THALIANA	Cytoplasm	GO annotation	

1EAGA	SECRETED ASPARTIC PROTEINASE (SAP2) FROM CANDIDA ALBICANS COMPLEXED WITH A70450	Extracellular	GO annotation	
1EB6A	DEUTEROLYSIN FROM ASPERGILLUS ORYZAE			
1EDOA	THE X-RAY STRUCTURE OF BETA-KETO ACYL CARRIER PROTEIN REDUCTASE FROM BRASSICA NAPUS COMPLEXED WITH NADP+	Chloroplast	GO annotation	
1EK0A	GPPNHP-BOUND YPT51 AT 1.48 A RESOLUTION	Both Extracellular and cytoplasm	GO annotation	
1EKMA	CRYSTAL STRUCTURE AT 2.5 A RESOLUTION OF ZINC-SUBSTITUTED COPPER AMINE OXIDASE OF HANSENULA POLYMORPHA EXPRESSED IN ESCHERICHIA COLI	Cytoplasm	GO annotation	
1EN2A	UDA TETRASACCHARIDE COMPLEX. CRYSTAL STRUCTURE OF URTICA DIOICA AGGLUTININ, A SUPERANTIGEN PRESENTED BY MHC MOLECULES OF CLASS I AND CLASS II	Extracellular	Complex with MHC class I and II molecules	
1ENPA	BRASSICA NAPUS ENOYL ACP REDUCTASE/NADH BINARY COMPLEX AT PH 8.0 AND ROOM TEMPERATURE	Chloroplast	GO annotation	
1EQKA	SOLUTION STRUCTURE OF ORYZACYSTATIN-I, A CYSTEINE PROTEINASE INHIBITOR OF THE RICE, ORYZA SATIVA L. JAPONICA	Extracellular	GO annotation	
1ERJA	CRYSTAL STRUCTURE OF THE C-TERMINAL WD40 DOMAIN OF TUP1	Cytoplasm	GO annotation	
1EYLA	STRUCTURE OF A RECOMBINANT WINGED BEAN CHYMOTRYPSIN INHIBITOR			
1EZVE	STRUCTURE OF THE YEAST CYTOCHROME BC1 COMPLEX CO-CRYSTALLIZED WITH AN ANTIBODY FV-FRAGMENT	Both Extracellular and cytoplasm	GO annotation	
1F9MA	CRYSTAL STRUCTURE OF THIOREDOXIN F FROM SPINACH CHLOROPLAST (SHORT FORM)	Chloroplast	GO annotation	
1FB6A	CRYSTAL STRUCTURE OF THIOREDOXIN M FROM SPINACH CHLOROPLAST (OXIDIZED FORM)	Chloroplast	GO annotation	
1FNCA	REFINED CRYSTAL STRUCTURE OF SPINACH FERREDOXIN REDUCTASE AT 1.7 ANGSTROMS RESOLUTION: OXIDIZED, REDUCED, AND 2'-PHOSPHO-5'-AMP BOUND STATES	Both Extracellular and cytoplasm	GO annotation	
1G66A	ACETYLXYLAN ESTERASE AT 0.90 ANGSTROM RESOLUTION	Extracellular	GO annotation	

1GPSA	SOLUTION STRUCTURE OF GAMMA 1-H AND GAMMA 1-P THIONINS FROM BARLEY AND WHEAT ENDOSPERM DETERMINED BY 1H-NMR: A STRUCTURAL MOTIF COMMON TO TOXIC ARTHROPOD PROTEINS	Extracellular	GO annotation	
1GPTA	SOLUTION STRUCTURE OF GAMMA 1-H AND GAMMA 1-P THIONINS FROM BARLEY AND WHEAT ENDOSPERM DETERMINED BY 1H-NMR: A STRUCTURAL MOTIF COMMON TO TOXIC ARTHROPOD PROTEINS	Extracellular	GO annotation	
1GU7A	ENOYL THIOESTER REDUCTASE FROM CANDIDA TROPICALIS	Mitochondria	GO annotation	
1H49A	CRYSTAL STRUCTURE OF THE INACTIVE DOUBLE MUTANT OF THE MAIZE BETA-GLUCOSIDASE ZMGLU1-E191D-F198V IN COMPLEX WITH DIMBOA-GLUCOSIDE	Both Extracellular and cytoplasm	The subcellular compartmentation of beta-glucosidase was studied in rye, maize and wheat seedlings by immunocytochemical methods. For detection, we used a 10 nm gold-labeled secondary antibody, and results were observed using transmission electron microscopy. In all three species, beta-glucosidase was found in plastids, cytoplasm and cell walls	Physiol Plant. 2001 Apr ;111 (4):466-472 Subcellular localization of beta-glucosidase in rye, maize and wheat seedlings. [My paper] Jeanette Nikus, Geoffrey Daniel, Lisbeth M. V. Jonsson
1H4PA	CRYSTAL STRUCTURE OF EXO-1,3-BETA GLUCANSE FROM SACCHAROMYCES CEREVISIAE	Extracellular	GO annotation	
1H5QA	MANNITOL DEHYDROGENASE FROM AGARICUS BISPORUS	Extracellular	However, previous work has found that pathogen-secreted mannitol is extracellular, while MTD in uninfected plants is cytoplasmic. However, more recent immunolocalization studies, together with analysis of epitope-tagged MTD in cultured cells, suggest that MTD is secreted in response to the pathogen defense response inducer salicylic acid.	Pathogen-induced secretion of the normally cytoplasmic enzyme mannitol dehydrogenase (MTD) in plants.Ceng, Fang-yi (A) Zamski, Eli (B) Pharr, Mason (A) Williamson, John
1HG8A	ENDOPOLYGALACTURONASE FROM THE PHYTOPATHOGENIC FUNGUS FUSARIUM MONILIFORME Authors Federici, L., Caprari, C.,			
1HSSA	0.19 ALPHA-AMYLASE INHIBITOR FROM WHEAT	Extracellular	GO annotation	
1I24A	HIGH RESOLUTION CRYSTAL STRUCTURE OF THE WILD-TYPE PROTEIN SQD1, WITH NAD AND UDP-GLUCOSE	Chloroplast	GO annotation	
1IA5A	POLYGALACTURONASE FROM ASPERGILLUS ACULEATUS	Extracellular	GO annotation	
1IBQA	ASPERGILLOPEPSIN FROM ASPERGILLUS PHOENICIS	Extracellular	GO annotation	
1IOOA	CRYSTAL STRUCTURE OF NICOTIANA ALATA GEMETOPHYTIC SELF-INCOMPATIBILITY ASSOCIATED SF11-RNASE	Extracellular	GO annotation	

1JDRA	Crystal Structure of a Proximal Domain Potassium Binding Variant of Cytochrome c Peroxidase	Mitochondria	GO annotation	
1JEHA	CRYSTAL STRUCTURE OF YEAST E3, LIPOAMIDE DEHYDROGENASE	Cytoplasm,Mitochondrial	GO annotation	
1JMPA	Solution Structure of the Viscotoxin B	Extracellular	GO annotation	
1JR8A	Crystal Structure of Erv2p	Both Extracellular and cytoplasm	GO annotation	
1K0DA	Ure2p in Complex with Glutathione	Cytoplasm	GO annotation	
1K0DD	Ure2p in Complex with Glutathione	Cytoplasm	GO annotation	
1KBIA	Crystallographic Study of the Recombinant Flavin-binding Domain of Baker's Yeast Flavocytochrome b2: Comparison with the Intact Wild-type Enzyme	Mitochondria	GO annotation	
1KSIA	CRYSTAL STRUCTURE OF A EUKARYOTIC (PEA SEEDLING) COPPER-CONTAINING AMINE OXIDASE AT 2.2A RESOLUTION	Extracellular	Pea seedling copper amine oxidase has a 25 amino acid leader sequence characteristic of a secretion signal peptide as expected for an extracellular enzyme.Plant copper amine oxidases are generally found in the apoplast, loosely associated with the cell wall. Oxidation of di and polyamines present within the apoplast and the H2O2 formed by their oxidation may be important in lignosuberization and cross-linking of extracellular macromolecules such as extensisns	
1LGYA	LIPASE II FROM RHIZOPUS NIVEUS	Extracellular	GO annotation	
1LJPA	Crystal Structure of beta-Cinnamomin Elicitin	Extracellular	GO annotation	
1LLFA	Cholesterol Esterase (Candida Cylindracea) Crystal Structure at 1.4A resolution			
1LRHA	Crystal structure of auxin-binding protein 1 in complex with 1-naphthalene acetic acid	Cytoplasm	GO annotation	
1M2OB	Crystal Structure of the Sec23-Sar1 complex	Both Extracellular and cytoplasm	GO annotation	

1M2TA	Mistletoe Lectin I from Viscum album in Complex with Adenine Monophosphate. Crystal Structure at 1.9 A Resolution	Extracellular	There are a>number of ways by which vacuolar lectins can interact with molecules within and outside the cell. First, when dry seeds imbibe water, vacuolar proteins and especially lectins are released into the imbibition water (Fountain et al., 1977). This results in the presence of lectins in the vicinity of the germinating seed, where they can interact with potential pathogens. Second, when seeds<br>or other plant organs are eaten by predators, lectins will be released from the disrupted cellular structures of the plant tissues. These lectins will then come in contact with<br>the glycoproteins that line the intestinal tracts of the predators,<br>possibly inhibiting absorption of nutrients. Third,<br>when funga1 hyphae grow into plant tissues, they may disrupt cellular compartmentation, causing the release of	
1MPPA	X-RAY ANALYSES OF ASPARTIC PROTEINASES. V. STRUCTURE AND REFINEMENT AT 2.0 ANGSTROMS RESOLUTION OF THE ASPARTIC PROTEINASE FROM MUCOR PUSILLUS	Extracellular	Two closely related species of Zygomycete fungus, Mucor pusillus and Mucor miehei, secrete aspartate proteases, also known as mucor rennins, into the medium.	PRODUCTION OF EXTRACELLULAR PROTEASES BY MUCOR CIRCINELLOIDES USING D-GLUCOSE AS CARBON SOURCE / SUBSTRATE<br>Vânia Sousa Andrade1; Leonie Asfora Sarubbo2; Kasutaka Fukushima3; Makoto Miyaji3; Kazuko Nishimura3; Galba Maria de Campos-Takaki2*Braz. J. Microbiol. vol.33 no.2 São Paulo Apr / June 2002
1MRGA	STUDIES ON CRYSTAL STRUCTURES ACTIVE CENTER GEOMETRY AND DEPURINE MECHANISM OF TWO RIBOSOME-INACTIVATING PROTEINS	Both Extracellular and cytoplasm	s. In mature seeds luffin is accumulated within protein bodies in the storage tissue; vacuolar compartmentation in cells of the cotyledonary leaves is maintained during germination of the seedlings. In adult tissues, such as mature leaves and stems, the targeting of the protein is different, since luffin is found in the extracellular spaces. This localization outside the plasma membrane has been confirmed by enzymatic activity determination on the intercellular fluid present in the apoplastic space	Localization of the type I ribosome-inactivating protein, luffin, in adult and embryonic tissues of Luffa cylindrica L. Roem.DI COLA A. (1) ; MARCOZZI G. (1) ; BALESTRINI R. (2) ; SPANO L. (1) ;Journal of experimental botany1999, vol. 50, no334, pp. 573-579
1MVZA	NMR solution structure of a Bowman Birk inhibitor isolated from snail medic seeds (Medicago Scutellata)	Extracellular	GO annotation	

1NBLA	NMR Structure of Hellethionin D	Extracellular	Thionins belong to a rapidly growing family of biologically active peptides in the plant kingdom. Thionins are small (~5 kDA), cysteine-rich peptides with toxic and antimicrobial properties. They show a broad cellular toxicity against wide range of organisms and eukaryotic cell lines; while possessing some selectivity. Thionins are believed to be involved in protection against plant pathogens, including bacteria and fungi, by working directly at the membrane	Review<br>Plant thionins – the structural perspective<br>B. Stec,Cell. Mol. Life Sci. 63 (2006) 1370–1385
1NHCA	Structural insights into the processivity of endopolygalacturonase I from Aspergillus niger	Extracellular	GO annotation	
1NIOA	Crystal structure of beta-luffin, a ribosome inactivating protein at 2.0A resolution			
1NLSA	CONCANAVALIN A AND ITS BOUND SOLVENT AT 0.94A RESOLUTION	Extracellular	Bernhard and Avrameas (1971) have described a technique for the visualization of concanavalin binding sites on the cell membrane . By employing this technique, we have tried to localize concanavalin A receptor sites on the surface of hybrid cells and of the parent cells .	
1NM7A	Crystal Structure of the GluR2 Ligand Binding Core (S1S2J) in Complex with Quisqualate in a Zinc Crystal Form at 1.65 Angstroms Resolution	Both Extracellular and cytoplasm	GO annotation	
1NRJB	Signal Recognition Particle Receptor Beta-Subunit in Complex with the SRX Domain from the Alpha-Subunit	Both Extracellular and cytoplasm	GO annotation	
1OD5A	CRYSTAL STRUCTURE OF GLYCININ A3B4 SUBUNIT HOMOHEXAMER	Cytoplasm	GO annotation	
1OKHA	VISCOTOXIN A3 FROM VISCUM ALBUM L.	Extracellular	GO annotation	
1OM0A	crystal structure of xylanase inhibitor protein (XIP-I) from wheat	Extracellular	GO annotation	

1ONKA	Mistletoe lectin I from viscum album	Extracellular	There are number of ways by which vacuolar lectins can interact with molecules within and outside the cell. First, when dry seeds imbibe water, vacuolar proteins and especially lectins are released into the imbibition water (Fountain et al., 1977). This results in the presence of lectins in the vicinity of the germinating seed, where they can interact with potential pathogens. Second, when seeds or other plant organs are eaten by predators, lectins will be released from the disrupted cellular structures of the plant tissues. These lectins will then come in contact with the glycoproteins that line the intestinal tracts of the predators, possibly inhibiting absorption of nutrients. Third, when funga1 hyphae grow into plant tissues, they may disrupt cellular compartmentation, causing the release of	
1ONKB	Mistletoe lectin I from viscum album	Extracellular		
1PA2A	ARABIDOPSIS THALIANA PEROXIDASE A2	Extracellular	GO annotation	
1PLCA	ACCURACY AND PRECISION IN PROTEIN CRYSTAL STRUCTURE ANALYSIS: RESTRAINED LEAST-SQUARES REFINEMENT OF THE CRYSTAL STRUCTURE OF POPLAR PLASTOCYANIN AT 1.33 ANGSTROMS RESOLUTION	Both Extracellular and cytoplasm	GO annotation	
1Q2BA	CELLOBIOHYDROLASE CEL7A WITH DISULPHIDE BRIDGE ADDED ACROSS EXO-LOOP BY MUTATIONS D241C AND D249C	Extracellular	GO annotation	
1Q4MA	X-Ray Structure of Gene Product From Arabidopsis Thaliana AT3G16990			
1QFZA	PEA FNR Y308S MUTANT IN COMPLEX WITH NADPH	Both Extracellular and cytoplasm	GO annotation	
1QGJA	ARABIDOPSIS THALIANA PEROXIDASE N	Extracellular	GO annotation	
1QMGA	ACETOHYDROXYACID ISOMEROREDUCTASE COMPLEXED WITH ITS REACTION PRODUCT DIHYDROXY-METHYLVALERATE, MANGANESE AND ADP-RIBOSE.	Chloroplast	GO annotation	
1R64A	The 2.2 A crystal structure of Kex2 protease in complex with Ac-Arg-Glu-Lys-boroArg peptidyl boronic acid inhibitor	Both Extracellular and cytoplasm	GO annotation	
1RFSA	RIESKE SOLUBLE FRAGMENT FROM SPINACH	Both Extracellular and cytoplasm	GO annotation	
1RMGA	RHAMNOGALACTURONASE A FROM ASPERGILLUS ACULEATUS	Extracellular	GO annotation	

1S4VA	The 2.0 A crystal structure of the KDEL-tailed cysteine endopeptidase functioning in programmed cell death of Ricinus communis endosperm	Cytoplasm	GO annotation	
1SRDA	THREE-DIMENSIONAL STRUCTURE OF CU,ZN-SUPEROXIDE DISMUTASE FROM SPINACH AT 2.0 ANGSTROMS RESOLUTION	Chloroplast	GO annotation	
1TABI	STRUCTURE OF THE TRYPSIN-BINDING DOMAIN OF BOWMAN-BIRK TYPE PROTEASE INHIBITOR AND ITS INTERACTION WITH TRYPSIN			
1TMQB	STRUCTURE OF TENEBRIO MOLITOR LARVAL ALPHA-AMYLASE IN COMPLEX WITH RAGI BIFUNCTIONAL INHIBITOR	Extracellular	GO annotation	
1UCAA	Crystal structure of the Ribonuclease MC1 from bitter gourd seeds complexed with 2'-UMP			
1XYOA	STRUCTURAL COMPARISON OF TWO MAJOR ENDO-1,4-BETA-XYLANASES FROM TRICHODREMA REESEI	Extracellular	Endo-β-1,4-xylanases (EC 3.2.1.8) are key enzymes in the degradation of xylan, the predominant hemicellulose in the cell walls of plants and the second most abundant polysaccharide on earth. A number of endoxylanases are produced by microbial phytopathogens responsible for severe crop losses. These enzymes are considered to play an important role in phytopathogenesis, as they provide essential means to the attacking organism to break through the plant cell	Microbial Endoxylanases: Effective Weapons to Breach the Plant Cell-Wall Barrier or, Rather, Triggers of Plant Defense Systems? Tim Beliën,1 Steven Van Campenhout,1 Johan Robben,2 and Guido Volckaert1,Vol. 19, No. 10, 2006, pp. 1072–1081
2AAAA	CALCIUM BINDING IN ALPHA-AMYLASES: AN X-RAY DIFFRACTION STUDY AT 2.1 ANGSTROMS RESOLUTION OF TWO ENZYMES FROM ASPERGILLUS	Extracellular	A rapid induction system for synthesis of alpha-amylase by the funga Aspergillus oryzae M-13 was established. The mycelia were prepared from 20-h cultures grown on a peptone-glycerol medium and starved for 5 h; maltose was the optimum inducer tested. During h 1 of induction, formation of both intra- and Extracellular alpha-amylases occurred at an almost identical rate (70 to 80 microgram/g of cells-h) without a detectable lag period	Rapid induction of alpha-amylase by nongrowing mycelia of Aspergillus oryzae. M Yabuki, N Ono, K Hoshino and S Fukui
2ASIA	ASPARTIC PROTEINASE	Extracellular	Plant aspartic proteinases are generally either secreted or targeted to the vacuolar/protein storage body compartment.	Plant aspartic proteinases: enzymes on the way to a function
2HVMA	HEVAMINE A AT 1.8 ANGSTROM RESOLUTION	Intracellular	GO annotation	
2PLHA	STRUCTURE OF ALPHA-1-PUROTHIONIN AT ROOM TEMPERATURE AND 2.8 ANGSTROMS RESOLUTION	Extracellular	GO annotation	

2PLTA	STRUCTURE DETERMINATION OF PLASTOCYANIN FROM A CRYSTAL SPECIMEN WITH HEMIHEDRAL TWINNING FRACTION OF ONE-HALF	Both Extracellular and cytoplasm	GO annotation	
3GCBA	GAL6 (YEAST BLEOMYCIN HYDROLASE) MUTANT C73A/DELTAK454	Intracellular	GO annotation	
7MDHA	STRUCTURAL BASIS FOR LIGHT ACITVATION OF A CHLOROPLAST ENZYME. THE STRUCTURE OF SORGHUM NADP-MALATE DEHYDROGENASE IN ITS OXIDIZED FORM	Intracellular	GO annotation	
