# Supplementary material for: Analysis on conservation of disulphide bonds and their structural features in homologous protein domain families
Source: BMC Struct Biol. 2008 Dec 26;8:55. doi: 10.1186/1472-6807-8-55 (PMC2628669; doi:10.1186/1472-6807-8-55)
Supplement: Additional file 8 — Conservation of disulphide bonds in protein chains highly predicted as cytoplasmic. This table provides the results of examination of conservation (or otherwise) of distinct disulphides for proteins that have been highly predicted to be cytoplasmic. [file 1472-6807-8-55-S8.pdf]

Supplementary Table 3: Conservation of disulphide bonds in protein chains highly predicted as cytoplasmic

PDB ID	Name	Conclusion	Disulphide bond	Scop domain	Domain Name
1AOGA	TRYPANOSOMA CRUZI TRYPANOTHIONE REDUCTASE (OXIDIZED FORM)	Intracellular	SSBOND 1 CYS A 53 CYS A 58<br>SSBOND 2 CYS B 53 CYS B 58	c.3.1.5	Trypanothione reductase
1AQWA	GLUTATHIONE S-TRANSFERASE IN COMPLEX WITH GLUTATHIONE	Intracellular	No	c.47.1.5	Class pi GST
1CYDA	CARBONYL REDUCTASE COMPLEXED WITH NADPH AND 2-PROPANOL	Intracellular	No	c.2.1.2	Carbonyl reductase
1D2EA	CRYSTAL STRUCTURE OF IntracellularL EF-TU IN COMPLEX WITH GDP	Intracellular	No	c.37.1.8	Elongation factor Tu (EF-Tu), N-terminal (G) domain
1E6WA	RAT BRAIN 3-HYDROXYACYL-COA DEHYDROGENASE BINARY COMPLEX WITH NADH AND ESTRADIOL	Intracellular	No	c.2.1.2	Type II 3-hydroxyacyl-CoA dehydrogenase
1F14A	L-3-HYDROXYACYL-COA DEHYDROGENASE (APO)	Intracellular	No	c.2.1.6	Short chain L-3-hydroxyacyl CoA dehydrogenase
1F6BA	CRYSTAL STRUCTURE OF CHEMOKINE DOMAIN OF FRACTALKINE	Intracellular	No	c.37.1.8	RhoE (RND3)
1FSUA	4-SULFATASE (HUMAN)	Intracellular	SSBOND 1 CYS A 117 CYS A 521<br>SSBOND 2 CYS A 121 CYS A 155<br>SSBOND 3 CYS A 181 CYS A 192<br>SSBOND 4 CYS A 405 CYS A 447	c.76.1.2	Arylsulfatase B (4-sulfatase)
1FW1A	GLUTATHIONE TRANSFERASE ZETA/MALEYLACETOACETATE ISOMERASE	Intracellular	No	a.45.1.1	Class zeta GST
1GP1A	THE REFINED STRUCTURE OF THE SELENOENZYME GLUTATHIONE PEROXIDASE AT 0.2-NM RESOLUTION	Intracellular	No	c.47.1.10	Glutathione peroxidase
1GV4A	MURINE APOPTOSIS-INDUCING FACTOR (AIF)	Intracellular	No	d.87.1.1	Apoptosis-inducing factor (AIF)
1GZ6A	(3R)-HYDROXYACYL-COA DEHYDROGENASE FRAGMENT OF RAT PEROXISOMAL MULTIFUNCTIONAL ENZYME TYPE 2	Intracellular	No	c.2.1.2	(3R)-hydroxyacyl-CoA dehydrogenase domain of estradiol 17 beta-Dehydrogenase 4
1HD2A	HUMAN PEROXIREDOXIN 5	Intracellular	No	c.47.1.10	Peroxiredoxin 5
1HDRA	THE CRYSTALLOGRAPHIC STRUCTURE OF A HUMAN DIHYDROPTERIDINE REDUCTASE NADH BINARY COMPLEX EXPRESSED IN ESCHERICHIA COLI BY A CDNA CONSTRUCTED FROM ITS RAT HOMOLOGUE	Intracellular	No	c.2.1.2	Dihydropteridin reductase (pteridine reductase)
1I1IP	NEUROLYSIN (ENDOPEPTIDASE 24.16) CRYSTAL STRUCTURE	Intracellular	No	d.92.1.5	Neurolysin (endopeptidase 24.16)
1JTVA	Crystal structure of 17beta-Hydroxysteroid Dehydrogenase Type 1 complexed with Testosterone	Intracellular	No	c.2.1.2	Human estrogenic 17beta-hydroxysteroid dehydrogenase
1JWOA	Crystal Structure Analysis of the SH2 Domain of the Csk Homologous Kinase CHK	Intracellular	No	d.93.1.1	Csk homologous kinase Chk
1KEQA	Crystal Structure of F65A/Y131C Carbonic Anhydrase V, covalently modified with 4-chloromethylimidazole	Intracellular	No	b.74.1.1	Carbonic anhydrase
1M39A	Solution structure of the C-terminal fragment (F86-I165) of the human centrin 2 in calcium saturated form	Intracellular	No	a.39.1.5	Calmodulin-related protein NB-1 (CLP)
1M6IA	Crystal Structure of Apoptosis Inducing Factor (AIF)	Intracellular	No	c.3.1.5	Apoptosis-inducing factor (AIF)
1MJ4A	Crystal Structure Analysis of the cytochrome b5 domain of human sulfite oxidase	Intracellular	No	d.120.1.1	Sulfite oxidase, N-terminal domain
1MLDA	REFINED STRUCTURE OF IntracellularL MALATE DEHYDROGENASE FROM PORCINE HEART AND THE CONSENSUS STRUCTURE FOR DICARBOXYLIC ACID OXIDOREDUCTASES	Intracellular	No	d.162.1.1	Malate dehydrogenase

1N5DA	CRYSTAL STRUCTURE OF PORCINE TESTICULAR CARBONYL REDUCTASE/ 20BETA-HYDROXYSTEROID DEHYDROGENASE	Intracellular	No	c.2.1.2	Carbonyl reductase/20beta-hydroxysteroid dehydrogenase
1NN5A	Crystal structure of human thymidylate kinase with d4TMP + AppNHp	Intracellular	No	c.37.1.1	Thymidylate kinase
1Q41A	GSK-3 Beta complexed with Indirubin-3'-monoxime	Intracellular	No	d.144.1.7	Glycogen synthase kinase-3 beta (Gsk3b)
2AK3A	THE THREE-DIMENSIONAL STRUCTURE OF THE COMPLEX BETWEEN IntracellularL MATRIX ADENYLATE KINASE AND ITS SUBSTRATE AMP AT 1.85 ANGSTROMS RESOLUTION	Intracellular	No	g.41.2.1	Microbial and mitochondrial ADK, insert "zinc finger" domain
2PGDA	THE STRUCTURE OF 6-PHOSPHOGLUCONATE DEHYDROGENASE REFINED AT 2 ANGSTROMS RESOLUTION	Intracellular	No	a.100.1.1	6-phosphogluconate dehydrogenase (6PGD)
3GRSA	REFINED STRUCTURE OF GLUTATHIONE REDUCTASE AT 1.54 ANGSTROMS RESOLUTION	Intracellular	SSBOND 1 CYS A 58 CYS A 63	c.3.1.5	Glutathione reductase
4MDHA	REFINED CRYSTAL STRUCTURE OF IntracellularIC MALATE DEHYDROGENASE AT 2.5-ANGSTROMS RESOLUTION	Intracellular	No	d.162.1.1	Malate dehydrogenase
1CYJA	CYTOCHROME C6	Intracellular	No	b.1.18.2	Cyclomaltodextrin glycanotransferase, domain D
1DBYA	NMR STRUCTURES OF CHLOROPLAST THIOREDOXIN M CH2 FROM THE GREEN ALGA CHLAMYDOMONAS REINHARDTII	Intracellular	SSBOND 1 CYS A 31 CYS A 34	c.47.1.1	Thioredoxin
1E6BA	CRYSTAL STRUCTURE OF A ZETA CLASS GLUTATHIONE S-TRANSFERASE FROM ARABIDOPSIS THALIANA	Intracellular	No	c.47.1.5	Class sigma GST
1EDOA	THE X-RAY STRUCTURE OF BETA-KETO ACYL CARRIER PROTEIN REDUCTASE FROM BRASSICA NAPUS COMPLEXED WITH NADP+	Intracellular	No	c.2.1.2	beta-keto acyl carrier protein reductase
1EKMA	CRYSTAL STRUCTURE AT 2.5 A RESOLUTION OF ZINC-SUBSTITUTED COPPER AMINE OXIDASE OF HANSENULA POLYMORPHA EXPRESSED IN ESCHERICHIA COLI	Intracellular	SSBOND 1 CYS A 338 CYS A 364<br>SSBOND 2 CYS B 338 CYS B 364<br>SSBOND 3 CYS C 338 CYS C 364	d.17.2.1	Copper amine oxidase, domains 1 and 2
1ENPA	BRASSICA NAPUS ENOYL ACP REDUCTASE/NADH BINARY COMPLEX AT PH 8.0 AND ROOM TEMPERATURE	Intracellular	No	c.2.1.2	Enoyl-ACP reductase
1ERJA	CRYSTAL STRUCTURE OF THE C-TERMINAL WD40 DOMAIN OF TUP1	Intracellular	No	b.69.4.1	Tup1, C-terminal domain
1F9MA	CRYSTAL STRUCTURE OF THIOREDOXIN F FROM SPINACH CHLOROPLAST (SHORT FORM)	Intracellular	SSBOND 1 CYS A 46 CYS A 49<br>SSBOND 2 CYS B 46 CYS B 49	c.47.1.1	Thioredoxin
1FB6A	CRYSTAL STRUCTURE OF THIOREDOXIN M FROM SPINACH CHLOROPLAST (OXIDIZED FORM)	Intracellular	SSBOND 1 CYS A 37 CYS A 40<br>SSBOND 2 CYS B 37 CYS B 40	c.47.1.2	Thioredoxin
1GU7A	ENOYL THIOESTER REDUCTASE FROM CANDIDA TROPICALIS	Intracellular	No	b.35.1.2	2,4-dienoyl-CoA reductase
1I24A	HIGH RESOLUTION CRYSTAL STRUCTURE OF THE WILD-TYPE PROTEIN SQD1, WITH NAD AND UDP-GLUCOSE	Intracellular	No	c.2.1.2	c.2.1.2
1JDRA	Crystal Structure of a Proximal Domain Potassium Binding Variant of Cytochrome c Peroxidase	Intracellular	No	a.93.1.1	Cytochrome c peroxidase, CCP
1JEHA	CRYSTAL STRUCTURE OF YEAST E3, LIPOAMIDE DEHYDROGENASE	Intracellular	SSBOND 1 CYS A 44 CYS A 49<br>SSBOND 2 CYS B 44 CYS B 49	c.3.1.5	Dihydrolipoamide dehydrogenase
1K0DA	Ure2p in Complex with Glutathione	Intracellular	No	c.47.1.5	Yeast prion protein ure2p, nitrogen regulation fragment
1KBIA	Crystallographic Study of the Recombinant Flavin-binding Domain of Baker's Yeast Flavocytochrome b2: Comparison with the Intact Wild-type Enzyme	Intracellular	No	d.120.1.1	Flavocytochrome b2, N-terminal domain

1LRHA	Crystal structure of auxin-binding protein 1 in complex with 1-naphthalene acetic acid	Intracellular	SSBOND 1 CYS A 2 CYS A 155<br>SSBOND 2 CYS B 2 CYS B 155<br>SSBOND 3 CYS C 2 CYS C 155<br>SSBOND 4 CYS D 2 CYS D 155	b.82.1.2	Auxin binding protein
1OD5A	CRYSTAL STRUCTURE OF GLYCININ A3B4 SUBUNIT HOMOHEXAMER	Intracellular	SSBOND 1 CYS A 9 CYS A 42<br>SSBOND 2 CYS A 85 CYS A 327<br>SSBOND 3 CYS B 9 CYS B 42<br>SSBOND 4 CYS B 85 CYS B 327	b.82.1.2	Seed storage 7S protein
1QMG A	ACETOHYDROXYACID ISOMEROREDUCTASE COMPLEXED WITH ITS REACTION PRODUCT DIHYDROXY-METHYLVALERATE, MANGANESE AND ADP-RIBOSE.	Intracellular	No	c.2.1.6	Class II ketol-acid reductoisomerase (KARI)
1S4VA	The 2.0 A crystal structure of the KDEL-tailed cysteine endopeptidase functioning in programmed cell death of Ricinus communis endosperm	Intracellular	SSBOND 1 CYS A 23 CYS A 65<br>SSBOND 2 CYS A 57 CYS A 98<br>SSBOND 3 CYS A 156 CYS A 208<br>SSBOND 4 CYS B 23 CYS B 65<br>SSBOND 5 CYS B 57 CYS B 98<br>SSBOND 6 CYS B 156 CYS B 208	d.3.1.1	Vignain (bean endopeptidase)
1SRDA	THREE-DIMENSIONAL STRUCTURE OF CU,ZN-SUPEROXIDE DISMUTASE FROM SPINACH AT 2.0 ANGSTROMS RESOLUTION	Intracellular	SSBOND 1 CYS A 57 CYS A 146<br>SSBOND 2 CYS B 57 CYS B 146<br>SSBOND 3 CYS C 57 CYS C 146<br>SSBOND 4 CYS D 57 CYS D 146	b.1.8.1	Cu,Zn superoxide dismutase, SOD
2HVMA	HEVAMINE A AT 1.8 ANGSTROM RESOLUTION	Intracellular	SSBOND 1 CYS A 20 CYS A 67<br>SSBOND 2 CYS A 50 CYS A 57<br>SSBOND 3 CYS A 159 CYS A 188	c.1.8.5	Hevamine A (chitinase/lysozyme)
3GCBA	GAL6 (YEAST BLEOMYCIN HYDROLASE) MUTANT C73A/DELTAK454	Intracellular	No	d.3.1.1	Bleomycin hydrolase
7MDHA	STRUCTURAL BASIS FOR LIGHT ACITVATION OF A CHLOROPLAST ENZYME. THE STRUCTURE OF SORGHUM NADP-MALATE DEHYDROGENASE IN ITS OXIDIZED FORM	Intracellular	SSBOND 1 CYS A 24 CYS A 29<br>SSBOND 2 CYS A 365 CYS A 377<br>SSBOND 3 CYS B 24 CYS B 29<br>SSBOND 4 CYS B 365 CYS B 377<br>SSBOND 5 CYS C 24 CYS C 29<br>SSBOND 6 CYS C 365 CYS C 377<br>SSBOND 7 CYS D 24 CYS D 29<br>SSBOND 8 CYS D 365 CYS D 377	d.162.1.1	Malate dehydrogenase
